# Modulatory Effects of Probiotics During Pathogenic Infections With Emphasis on Immune Regulation

**DOI:** 10.3389/fimmu.2021.616713

**Published:** 2021-04-08

**Authors:** Abdul Raheem, Lin Liang, Guangzhi Zhang, Shangjin Cui

**Affiliations:** ^1^Institute of Animal Sciences, Chinese Academy of Agricultural Sciences, Beijing, China; ^2^Scientific Observation and Experiment Station of Veterinary Drugs and Diagnostic Technology of Beijing, Ministry of Agriculture, Beijing, China

**Keywords:** antibiotic resistant bacteria, antibiotics alternative, probiotics, pathogenic infections, immunomodulating

## Abstract

In order to inhibit pathogenic complications and to enhance animal and poultry growth, antibiotics have been extensively used for many years. Antibiotics applications not only affect target pathogens but also intestinal beneficially microbes, inducing long-lasting changes in intestinal microbiota associated with diseases. The application of antibiotics also has many other side effects like, intestinal barrier dysfunction, antibiotics residues in foodstuffs, nephropathy, allergy, bone marrow toxicity, mutagenicity, reproductive disorders, hepatotoxicity carcinogenicity, and antibiotic-resistant bacteria, which greatly compromise the efficacy of antibiotics. Thus, the development of new antibiotics is necessary, while the search for antibiotic alternatives continues. Probiotics are considered the ideal antibiotic substitute; in recent years, probiotic research concerning their application during pathogenic infections in humans, aquaculture, poultry, and livestock industry, with emphasis on modulating the immune system of the host, has been attracting considerable interest. Hence, the adverse effects of antibiotics and remedial effects of probiotics during infectious diseases have become central points of focus among researchers. Probiotics are live microorganisms, and when given in adequate quantities, confer good health effects to the host through different mechanisms. Among them, the regulation of host immune response during pathogenic infections is one of the most important mechanisms. A number of studies have investigated different aspects of probiotics. In this review, we mainly summarize recent discoveries and discuss two important aspects: (1) the application of probiotics during pathogenic infections; and (2) their modulatory effects on the immune response of the host during infectious and non-infectious diseases.

## Introduction

The term probiotic is derived from the Greek word (πρoβιoτ*ικ*ó: πρó and $\beta \overset{,}{\iota }$óς) meaning “for life” (1, 2). Probiotics have a very old history since their first description; the first clinical trial investigating the remedial effects of probiotics in constipation was started in 1930 (3). Probiotics have a wide range of applications in poultry, livestock, aquaculture, and also in humans for the prevention and treatment of disorders, ailments, and infectious and non-infectious diseases (e.g., bacterial, viral, parasitic, or fungal diseases, nervous system disorders, obesity, cancer, and allergic problems), as well as preoperative and postoperative processes. Nowadays, probiotics are an inevitable part of human nutrition with elevated consumption levels through naturally and microbially fermented products with enormous amounts of viable beneficial microbes, such as fermented animal products, fermented fruits and their juices, and various other food products (4). Different probiotics like *Lactobacillu*s, *Lactococcus, Leuconostoc, Pediococcus, Enterococcus, Vagococcus, Bacillus, Clostridium butyricum, Micrococcus, Rhodococcus, Brochothrix, Kocuria, Pseudomonas, Aeromonas, Shewanella, Enterobacter, Roseobacter, Vibrio, Zooshikella, Flavobacterium*, and some yeasts are commonly used probiotics to control infectious diseases as well as to improve health and quality of aquaculture production (5, 6). The application of specific probiotics culture in the poultry and livestock industry has become very common in recent days. Many economically important poultry diseases like Salmonellosis, Clostridial diseases, Coccidiosis etc., respond positively during probiotics treatment (7). Genus *Bacillus, Pediococcus, Lactobacillus, Enterococcus, Streptococcus, Aspergillus*, and *Saccharomyces* are usually used in poultry (1).

To increase meat production and inhibit pathogenic growth, antibiotics are usually supplemented in the feed of poultry and livestock leading to the emergence of antibiotic-resistant bacteria. Antibiotic-resistant bacteria are becoming very common, presenting difficulties to the treatment of clinical infections with current chemotherapeutics, thus effective and novel strategies which will enable the host immune system to combat the infections are urgently needed (8). Probiotics exert beneficial effects to their hosts by diverse mechanisms, e.g., antimicrobial peptide (AMP) production, fatty acids production, stabilization of disturbed intestinal microflora, competitive pathogen exclusion, and modulation of host innate and adaptive immune responses (9). Nowadays, strategies using probiotics as an immunomodulator to control infectious diseases have become popular. Antimicrobial effects of probiotics by modulating the innate and adaptive immune responses of hosts have been extensively reported in numerous *in vitro* and *in vivo* studies.

Immune cells or epithelial cells can express a series of pattern recognition receptors (PRRs). The typical PRRs consist of Toll-like receptors (TLRs), retinoic acid-inducible gene-I-like receptors (RLRs), nucleotide oligomerization domain (NOD)-like receptors (NLRs), and C-type lectin receptors (10). Pathogen-associated molecular patterns (PAMPs) of probiotics interact with PRRs to initiate appropriate signaling pathways for the expression of different genes and subsequent production of immune mediators, which help the hosts to counteract the pathogenic infections (11). Besides these immune remedial effects, probiotics also provide other health-promoting effects on hosts. Indigenous microbiota possess different biological activities extending from anabolism to catabolism of large molecules, resulting in beneficial effects on host health as well as microbiota themselves. Intestinal microflora can ferment endogenous mucus and indigestible diet residues and produce vitamins, such as vitamin K and B (12). The following sections of this review provide a brief introduction to probiotics and discuss the mechanism of probiotic functions and their application during pathogenic infections.

## History of Probiotics

In the early 1900s, Louis Pasteur asserted that microorganisms were responsible for food fermentation, while Élie Metchnikoff stated that the increased longevity of individuals living in the rural areas of Bulgaria was closely associated with the daily consumption of fermented dairy products, such as yogurt. He claimed that lactobacilli could mitigate the putrefactive effects of gastrointestinal metabolism, which contributed to diseases and aging. Approximately 2,000 years earlier, Hippocrates claimed that “death sits in the bowl” (13). Fermented foods have a long history; fermented milk can be traced back to the Neolithic age. The fermentation of milk was first reported around 10,000 BC in the Middle East and India, and around 7,000–5,000 BC in Egypt, Rome, Greece, and the rest of Europe. The first appearance of soy sauce is estimated around 4,000 BC and 3,000 BC in China, Japan, and Korea; fermented rice first appeared around 2,000 BC in Asia. Fish sauce originated from northern Africa and South East Asia around 1,000 BC. The use of wine possibly started in North Africa around 3,000 BC, and subsequently expanded in the Middle East, Greece, Egypt, and Rome. The use of beer may have started around 7,000 BC in China and probably around 5,000 BC in Mesopotamia (2, 14) (Table 1).

**Table 1:** Some fermented foods history and origin.

**Food origin**	**Aproximate appearance year**	**Region**
Fermented milk	10,000 BC	Middle East
Product of fermented milk	7,000–5,000 BC	Egypt, Italy, Greece
Mushroom	4,000 BC	China
Wine	3,000 BC	North Africa, Middle East, Europe
Soy sauce	3,000 BC	China, Korea, Japan
Fermented honey	2,000 BC	Middle East, North Africa
Fermented rice	2,000 BC	China, Asia
Fermented malted cereals: beer	2,000 BC	China, Middle East, North Africa
Chees	2,000 BC	China, Middle East
Fermented meats	1,500 BC	Middle East
Bread	1,500 BC	Egypt, Europe
Pickled vegetables	1,000 BC	China, Europe
Fish sauce	1,000 BC	Southesat Asia, North Africa
Sourdough bread	1000 BC	Europe
Tea	200 BC	China

## Selection Criteria and Health Benefits of Commonly Used Probiotics

A number of microbes have been used as probiotics. The number of microbial organisms with probiotic characteristics is remarkable. Among them, lactic acid bacteria (LAB) are a group of non-spore forming, Gram-positive rods or cocci with tolerability to markedly low pH; they are fermenters of carbohydrates and use carbon as final electron acceptors. LAB have a wide range of applications and are the most commonly used probiotics (15, 16). They are classified on the basis of their cellular morphology and glucose fermentation mode, into Phylum-Firmicutes, Class-Bacilli, and Order-Lactobacillales. Currently, the LAB genera include *Lactobacillus, Streptococcus, Leuconostoc, Carnobacterium, Lactococcus, Aerococcus, Enterococcus, Pediococcus, Oenococcus, Weissella, Alloiococcus, Tetragenococcus, Dolosigranulum*, and *Vagococcus* (17, 18). The most frequently utilized genera of bacteria used in probiotic formulations are *Lactobacillus, Enterococcus, Streptococcus, Bacillus*, and *Bifidobacterium*, as well as some fungal strains of the genus *Saccharomyces*, such as *Saccharomyces boulardii* (*S. boulardii*). Most of these are regarded as the intestinal commensal microbiota (2).

**Figure 1:**
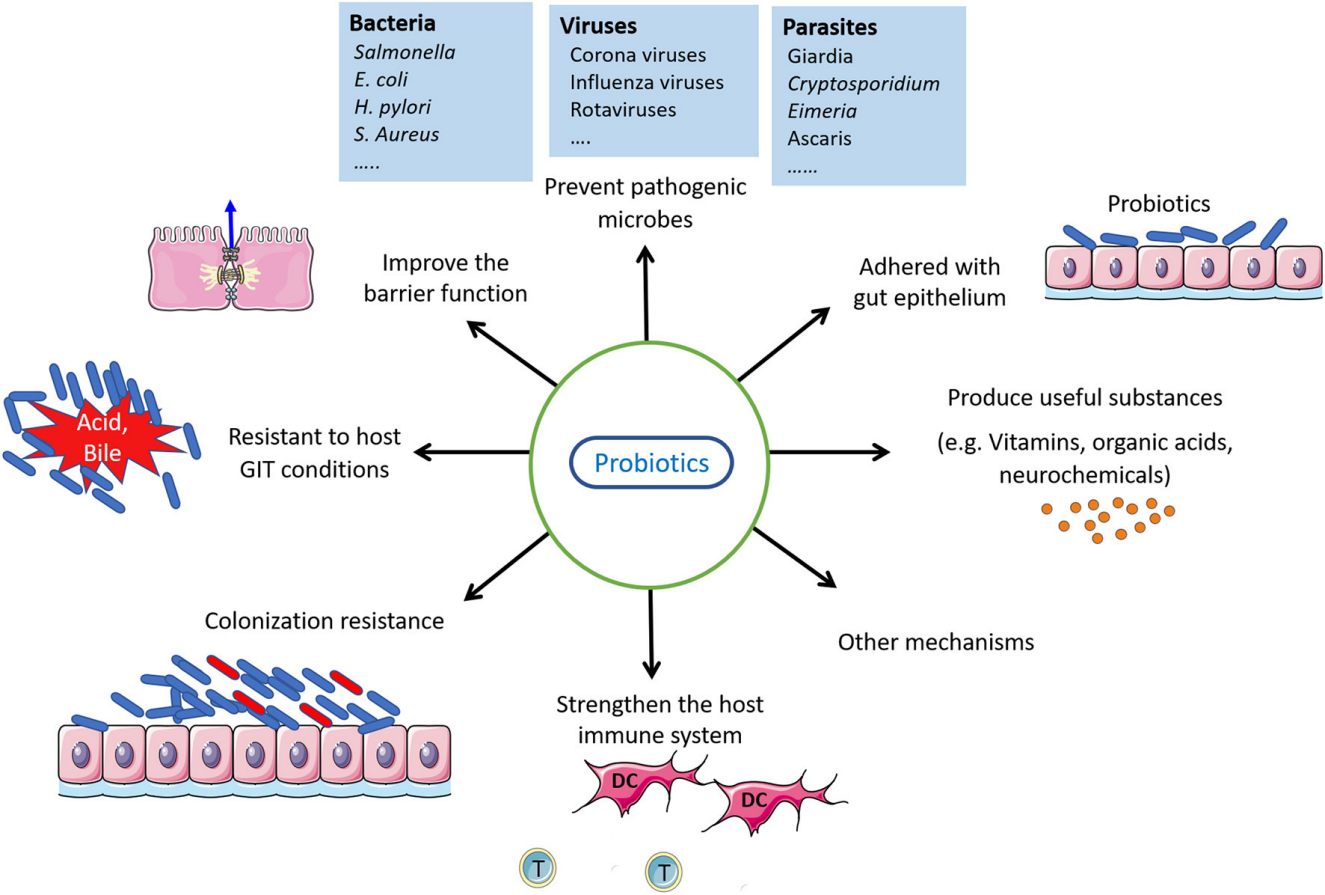
Probiotics properties and their action.

The process for the identification of newly-isolated probiotic candidates is the first problem that needs to be addressed. From isolation to market launching, knowledge needs to be collected on host health, adhesion properties, and resistance to host biochemical environments. Probiotics must be safe, adhere to the lining of intestinal cells with high survival potential, have an immunostimulatory function, have the ability to colonize the tract lumen, withstand exposure to low pH and bile salt, and should have antipathogenic characteristics (19, 20). Other probiotic properties may be considered for selecting probiotic strains with cognitive effects, such as their ability to lower cholesterol, antioxidant function, or cytotoxic impact on cancer cells. Of note, a prospective probiotic does not need to follow or meet all aforementioned selection criteria (21). Figure 1 shows some properties of good probiotics.

The microbiota inhabiting the animal body provide crucial services to the ecosystem, such as the production of important resources and bioconversion of different nutrients, which are beneficial for both the host and microbes. Microbiota can execute different crucial biological activities, ranging from anabolism to catabolism of large molecules. These biological activities can be beneficial for host health and the microbes. The metabolic functions of intestinal microflora reduce the energy costs of their host, as they ferment endogenous mucus and indigestible food residues, and also produce vitamins such as vitamin K and B (12). Therefore, due to their biological activities, probiotics have positive health effects on hosts, including reduction of the energy required during digestion and provision of beneficial nutrients. Different kinds of commercially available probiotics products are available to boost the health of adults and children (www.probioticchart.ca, www.usprobioticguide.come) (22).

## Probiotics Encapsulation

Because of the substantial decrease in their viability in the harsh gastrointestinal environment of the host (gastric pH, protease, lipase, and peristalsis) and during different food processing and storage conditions (high temperature, pH changes, oxygen, and hydrogen), the possible beneficial health effects of probiotics may not be recognized. A number of systems have been designed to improve orally administered probiotics viable number in gastrointestinal tract (GIT), including coating and embedding systems (23). Microencapsulation is an efficient technique that is used to increase the viability and resistance of probiotics against the harsh environmental conditions of GIT and during storage conditions. Microencapsulation is a physicochemical or mechanical process in which probiotics are usually inserted or coated with food-grade materials like lipids, biopolymers, or other hydrocolloidal materials, providing protection against adverse conditions such as heat shock, low pH, bile salts, cold shock, etc. (24). Several studies have been reported that microencapsulation increases the viability of probiotics Encapsulation of *Bifidobacterium longum* with milk increases its viability during storage time (25). *Lactococcus lactis* subsp. *cremoris* LM0230 encapsulation in alginate increases its stability and viability (26). Similarly, *Lactobacillus rhamnosus* GG encapsulation with pectin increases its viability in simulated GIT conditions. Muhammad et al. (27) reported *Lactobacillus plantarum* KLDS 1.0344 ability to alleviate chronic lead toxicity in mice increases when encapsulated with starch originated from tomatoes (27). The study of Riaz et al. (28) shows that the survival rate under simulated GIT conditions of zein-coated alginate *Bifidobacterium bifidum* significantly increases.

## Potential Mechanisms of the Probiotic Function

The mechanisms of probiotic function are complex, heterogeneous, and specific to probiotic strains. They include competitive exclusion of pathogens (29), ability to colonize the intestine (30), intestinal barrier function improvement by increasing the expression of tight junction proteins and mucin expression along with the interaction of PAMP to PRRs, AMP production (31), and immune system regulation. Some important mechanisms are briefly discussed below.

### Competitive Pathogen Exclusion

This refers to a condition in which one bacterial species has a greater potential to attach the epithelia, through a receptor, than other species (11). The known mechanisms of competitive exclusion mainly include lowering the pH in the lumen, contesting for nutrient utilization, and AMP production against competitors (32). Interaction between molecules distributed in the gut epithelia and the surface of bacterial cells mediates the adhesion and colonization of bacteria. Commensal or probiotic bacteria produce adhesive surface molecules (e.g., enolases, glyceraldehyde-3-phosphate, and pyruvate dehydrogenase) and adhere to the extracellular matrix of the host (33, 34). These adhesive surface molecules assist commensal bacteria and probiotics in contesting and preventing pathogenic bacterial attachment and colonization (35, 36). *Lactobacillus fermentum* (*L. fermentum*) competitively binds to collagen I of host epithelial cells by expressing its collagen-binding protein genes and inhibits the binding of *Campylobacter jejuni*. Similarly, *Lactobacillus gasseri* expresses aggregation-promoting factors on their cell surface, which helps in self-aggregation and its binding with the host extracellular matrix fibronectin component. This facilitates the colonization of probiotics and the exclusion of pathogens from the GIT (37). *L. gasseri* also inhibits the adhesion of *Helicobacter pylori* (*H. pylori*) to AGS gastric epithelial cell lines by expressing its Sortase A (srtA) gene, which produces surface molecules that facilitate *L. gasseri* aggregation, as well as binding and adhesion to AGS cell lines (38). Pretreatment with some probiotics impedes pathogenic bacterial attachment to host cell receptor sites by steric hindrance pose, and reduces the colonization of unwanted microbes by producing negative growth factors for pathogens (39). Seaweed *Bacillus* probiotics have good adhesion properties to shrimp intestinal mucosa with competitive exclusion ability and eliminate *Vibrio parahaemolyticus* strain 3HP (40).

Competitive exclusion of probiotics exerts the beneficial effects on the GIT and other organs of the host, increases the adhesion of probiotics, and performs protective actions against pathogens by competing for binding sites of the host. Furthermore, this adhesion of probiotics increases the opportunity for interaction with the host, which favors the immunostimulatory effects of probiotic surface molecules (ligands for receptors of the host) and their metabolites (41, 42). Therefore, the competitive exclusion properties of probiotics offer several benefits to host health, including the reduction of pathogenic attachment, colonization (many diseases arise because of pathogen colonization), further spread of the pathogen, and pathogenic load in hosts. Furthermore, this property of probiotics enables them to colonize the host GIT, which is necessary for the further beneficial action of probiotics to their hosts.

### Intestinal Colonization

The potential of probiotics to colonize the intestine is one of the most important properties recommended by WHO/The Food and Agriculture Organization of the United Nations (FAO). The positive characteristics of probiotics, such as antagonisms to harmful microbes or the modulation of the immune system, are linked to their intestinal colonization, which is investigated *in vitro* using simulated intestinal cells, as *in vivo* investigation is difficult (43). The adhesion of LAB with intestinal cells has been extensively reported. Interaction between molecules distributed on gut epithelia and the surface of bacterial cells mediates the adhesion and colonization of bacteria and is highly variable between different bacterial strains. García-Ruiz et al. (44) reported 0.37–12.2% adhesion of wine-isolated LAB (44) and Pisano et al. (45) reported 3–20% adhesion of LAB (45).

### Intestinal Barrier Function

As the intestinal epithelial barrier acts as a physical and biochemical barrier and is important for preventing systemic entry of toxins, bacteria, and other foreign unwanted compounds, so its integrity and full function are quite important. It has been reported in many studies that LAB can improve intestinal epithelial barrier damage induced by pathogenic infection (46–51). Probiotics possess a diverse mechanism of action to improve the intestinal barrier function and maintain homeostasis. “*Lactobacillus* contains a HSP27-inducible polyphosphate (poly P) fraction. Probiotic-derived polyphosphates, strengthen the epithelial barrier function and keep intestinal homeostasis through the integrin-p38 MAPK pathway” (52). *Lactobacillus casei* DN-114 001 and *Lactobacillus acidophilus* strain LB have the potential to improve intestinal epithelial barrier during *Escherichia coli* infection (53, 54). Strains of *Lactobacillus, Bifidobacterium*, and *Streptococcus* stimulate tight junction proteins (occludin, claudin-1) results in enhanced barrier stability (55). *L. plantarum* WCFS1 significantly increases occludin and ZO-1 in tight junction vicinity by TLR2 dependent pathway and protect tight junction disruption by toxins, pathogens, and cytokines (49). Qin et al., also showed that *L. plantarum* has protective effects on intestinal barrier by rearranging tight junction proteins (occludin, claudin-1, JAM-1 ZO-1) disturbed by *E. coli* and ameliorates barrier function (50). Another strain of *L. plantarum*, MB452 increases occludin expression and improves intestinal barrier integrity (46). *E. coli* Nissle 1917 (EcN) ameliorates *E. coli* induced intestinal epithelial barrier dysfunction by regulating the expression of occludin and claudin (56). *L. rhamnosus* (LR: MTCC-5897) and *L. fermentum* (LF: MTCC-5898) significantly improve the *E. coli* disturbed tight junction proteins (Occludin, ZO-1, cingulin-1, claudin-1) in Caco-2 cells (57).

Several other reports of Lactobacilli study have also been shown that *Lactobacilli* ameliorate the intestinal barrier damage and pro-inflammatory cytokines production induced by *Salmonella* (47, 58). Probiotics are also effective to improve malnutritional induced intestinal barrier damaged as indicated by the study of Garg et al. on a malnutritional mice model, in which they reported that *Lactobacillus reuteri* LR6 feeding significantly improves the intestinal morphology damaged during malnutrition (59).

### Antimicrobial Peptide Production

Different criteria are applied to AMP classification according to their source (animals, fungi, plants, and bacteria), mechanisms of action (AMP acting on cell surface molecules or intracellular components), structure (patterns of covalent bonding), and biosynthetic pathway (non-ribosomally synthesized or ribosomally synthesized) (60). Bacteriocins (AMP from prokaryotes) of LAB are classified into three classes: Class I, post-translationally modified (e.g., lantibiotics); Class II, non-modified, heat stable with size <10 kDa (e.g., pediocin PA1, leucocin A, plantaricin A, and enterocin X); and Class III, heat labile, large peptides with size >30 kDa (e.g., helveticin J) (16). Bacteriocins have low molecular weight and form pores in target cell membranes, leading to the death (61) of pathogenic bacteria, and also act as anti-cancerous agents. Furthermore, bacteriocins also possess immunomodulatory properties with pronounced anti-inflammatory effects during pathogenic infections. As bacteriocins are non-toxic, particularly those derived from LAB, they are used in food preservation. A number of studies showed that certain kinds of probiotics inhibit many types of pathogenic bacteria (*proteus* spp., *E. coli, Acinetobacter baumannii, Pseudomonas aeruginosa, Klebsiella pneumoniae, Listeria monocytogenes, Citrobacter freundii, H. pylori, Enterobacter aerogenes, Compylobacter jejuni, Micrococcus luteus, Salmonella* spp., *Shigella* spp., and some fungi) by the action of their bacteriocins (62). Bacteriocins from *Lactobacillus salivarius* inhibit foodborne and other medically important bacteria, such as *Listeria monocytogenes*, many genera of *staphylococcus, Neisseria gonorrhoeae, Bacillus*, and *Enterococcus*; the bacteriocins kill these bacteria by creating membrane pores and subsequent leakage of cellular material. Further, these bacteriocins also assist *L. salivarius* colonization in the intestine without showing any prominent adverse effects on other lactic acid bacteria (63) *L. plantarum* also exerts antimicrobial activities by producing many types of bacteriocins with antimicrobial effects against food spoilage bacteria, such as *Alicyclobacillus acidoterrestris* (64), *Salmonella* spp., *Listeria monocytogenes, Staphylococcus aureus*, and *E. coli*; thus, they may be used as preservatives for pork meat (65). Apart from bacteria, some bacteriocins from *L. plantarum* are also effective against yeast and molds, such as *Fusarium, Candida, Aspergillus*, and *Mucor* (66). Bacteriocins from other probiotic species markedly induce apoptosis and inhibit tumor formation, cancer cell proliferation, and membrane depolarization of cancer cells during treatment (61). There are different classification systems for AMP and, owing to their diverse mechanism of actions, they have a wide range of applications in humans and animals, as well as aquaculture fields (67). They inhibit growth and even kill diverse pathogens by creating pores in their cell membranes, as well as initiating appropriate immune responses.

### Immune Regulation

It is well-established that probiotic bacteria exert an immunomodulatory effect and have the potential to communicate and interact with a series of immune cells (e.g., DCs, lymphocytes, epithelial cells, monocytes, and macrophages). The immune response generally comprises the innate immune response and adaptive immune response. Innate immune response responds to PAMPs distributed on the majority of bacteria (11). The principle immune response to any pathogen is activated following the interaction of PRRs (i.e., TLRs, NLRs, and C-type lectin receptors) with PAMPs and initiates cell signaling. Intestinal epithelial cells are the host cells that most extensively come into contact with probiotics. However, probiotics may also interact with DCs, which play a significant role in the innate immune response and bridge the innate and adaptive immune responses. Through their PRRs, both intestinal epithelial cells and DCs can communicate and react to gut microorganisms (68, 69). Under the effects of probiotics/commensal microbiota, the activated DCs induce the appropriate immune response (e.g., naïve CD4 T cells to Treg cell differentiation), which generally inhibits Th1-, Th2-, and Th17-mediated inflammatory response. Furthermore, probiotics blunt intestinal inflammation (70) by downregulating the expression of TLRs *via* secretion of TNF-α inhibitory metabolites and inhibition of nuclear factor-κB (NF-κB) signaling in enterocytes (68). Probiotics also modulate the expression of various kinds of cytokine production.

#### Cytokines Mediated Immune Response and Probiotics

Probiotic benefits related to immunoregulation for the treatment of various diseases have been extensively studied. Immunomodulatory effects of probiotics are mainly due to the induction of the release of cytokines including interleukins, transforming growth factor (TGF), tumor necrosis factors (TNFs), interferons (INFs), and immune cells released chemokines, which further regulate the immune system (71, 72). Immunostimulatory and immunoregulatory actions of probiotics have been reported in various studies. Immunostimulatory probiotics are capable of acting against infection and cancer cells, inducing the release of IL-12, which stimulates the NK cells and produces the Th1 cells. By maintaining the balance between Th1 and Th2, these probiotics also work against allergies. Contrary to this finding, immunoregulatory probiotics are attributed to Treg cells and IL-10 production to blunt excessive inflammatory responses, inflammatory bowel disease, and autoimmune disorders (73, 74). So, probiotics immunomodulatory effects *via* cytokines are strain-specific as indicated by the *in vitro* study of Haller et al. (75) using Caco-2 cells in which they reported that *Lactobacillus sakei* is capable of inducing pro-inflammatory cytokines (IL-1β, TNFα, and IL-8) whereas *Lactobacillus johnsonii* induced anti-inflammatory cytokines (TGF-β) (75). A mixture of *Lactobacillus paracasei* and *L. reuteri* to *Helicobacter hepaticus* IL-10-defcient mice leads to reduced colitis and pro-inflammatory cytokines production (76). Kourelis et al. (77) study on Fisher-344 inbred rats and BALB/c, demonstrated that *L. acidophilus* NCFB 1748 and *L. paracasei* subsp. *Paracasei* DC412 induce specific immune markers and innate immune responses *via* recruiting polymorphonuclear cell and production of TNFα (77). Probiotics-induced cytokines expression for immune system modulation of the host has been briefly discussed in the relevant section.

#### Toll-Like Receptor-Mediated Immune Response and Probiotics

Toll-like receptors and single-pass membrane-spanning receptors are very important protein receptors expressed on several non-immune (epithelial, fibroblasts) and immune [macrophages, B cells, natural killer (NK) cells, DCs] cells. Activation of the TLR signaling pathway, except TLR3 (78), generally leads to the recruitment of MyD88, which results in activation of the NF-κB and mitogen-activated protein kinase (MAPK) pathway. TLR-induced signaling is also responsible for the maturation of DCs characterized by increased expression levels of DC markers (CD80, CD83, and CD86) and chemokines receptor C-C motif chemokine receptor 7 (CCR7). TLR9 is crucial for the mediation of the anti-inflammatory effects of probiotics, though many other receptors are also involved.

Lactobacilli ligands initiate cell signaling after binding to TLR2 in combination with TLR6, endorsing dimerization and NF-κB activation *via* recruitment of MyD88 (79). Engagement of a bacterial ligand with TLR2 results in cytokine production and increases the transepthelial resistance for conquering microbes (79, 80). Several *Lactobacillus* strains induce their immunomodulatory effects by binding to TLR2, which recognizes peptidogycan (a component of the cell wall of Gram-positive bacteria). An *in vitro* study showed that *L. plantarum* and *L. rhamnosus* increased TLR2 expression in human cells (Caco-2). *L. casei* showed similar effects in *Salmonella*-infected and healthy mice, and induced TLR expression, as well as interleukin-10 (IL-10), interferon-gamma (IFN-γ), and TNF-α production (81, 82).

Numerous other probiotics interact with TLR4 to induce an appropriate immune response. For example, during pre-and post-*Salmonella* challenges in mice, *L. casei* increased the production of IL10, IFN-γ, and IL6, and reduced the levels of TNF-α by interacting with TLR4 (82, 83). Likewise, *L. rhamnosus* GG (heat-inactivated) and *Lactobacillus delbrueckii* subsp. *Bulgaricus* (*L. delbrueckii*) reduce TLR4 expression in DCs (human monocyte-derived) (84). TLR9, another important TLR, identifies bacterial CpG DNA and CpG-ODN (engineered unmethylated oligonucleotide mimics). Unmethylated pieces of DNA comprising CpG patterns produced from probiotics also have the propensity to mediate anti-inflammatory activities *via* TLR9.

In the case of the differentiated epithelium, apical, and basolateral stimulation results in the activation of different signaling pathways. Basolateral TLR9 activation causes activation of the NF-κB cascade by the degradation of IκBα. Of note, apical activation of TLR9 results in the suppression of NF-κB by the aggregation of ubiquitinated IκB in the cytoplasm (85). Apical or basolateral stimulation of these receptors is important and involves different signaling cascades leading to various immune responses. The results from the study conducted by Ghadimi et al. show that polarized T84 and HT-29 cells increase TLR9 expression in a specific manner in response to apically applied natural commensal origin DNA. They reported that when LGG DNA is applied to these cells, it attenuates TNF-α enhanced NF-κB activity by reducing IκBα degradation and p38 phosphorylation (86).

*Lactobacillus plantarum-*purified DNA also modulates the immune response of host cells by interacting with TLRs, as reported by Kim, whose studies show that *L. plantarum-*purified DNA inhibits LPS induced TNF-α production in THP-1 cells. Furthermore, *L. plantarum-*purified DNA blunt the expression of TLR4, TLR2, and TLR9, which induce NF-κB activation through the LPS signaling pathway, leading to pro-inflammatory cytokines upregulation (87, 88). TLRs are important membrane receptors; most intracellular signaling pathways involve the activation of membrane receptors. Furthermore, TLRs play a key role in the induction of immune response by probiotics through the recruitment of specific intracellular signaling molecules. Depending on their interaction with specific TLRs, probiotics may decrease or increase TLR expression.

#### NLR-Mediated Immune Response and Probiotics

In tissues with blunt TLR expression, NLRs are important and present in the cytoplasm. Thus far, more than 20 NLRs have been recognized. Among them, NOD1 and NOD2 are the most studied and important NLRs (89). NOD1 is expressed in many cells and recognizes peptidoglycan moieties (comprising Gram-negative meso diaminopimelic acid). NOD2 is mainly expressed on DCs, lungs cells, macrophages, intestinal cells, buccal epithelium, and Paneth cells. It senses muramyl dipeptide motifs which are present in a wide range of bacteria (90). NOD1 and NOD2 undergo self-oligomerization following recognition by their agonist. This results in the recruitment and activation of receptor interacting serine/threonine kinase 2 (RICK; an adaptor protein, kinase responsible for the regulation of apoptosis *via* CD95), which is necessary for MAPKs and NF-κB activation and the subsequent production of inflammatory mediators such as cytokines and chemoattractants. Another important signaling factor that NLRs trigger is, apoptosis-associated speck-like protein with caspase induction to trigger caspase 1 (CASP1; an adaptor protein required for the functionally effective and mature forms of pro IL18 and pro IL1). NLRs are involved in the formation of the inflammasome that results in CASP1 activation. There are three major inflammasomes named according to the NLRs involved: NOD-like receptor family pyrin domain containing protein 1 (NLRP1), NLRP3, and NLRC4. Murymyl dipeptide, bacterial and viral RNA, and lipopolysaccharides are sensed by NLRP3 (91). Many *Lactobacillus* species exert their immune regulatory effects *via* NLRs. In galactose-1-phosphate uridylyltransferase (GALT) of swine, *L. gasseri* and *L. delbrueckii* increase the expression of NLRP3 *via* TLR and the NOD signaling cascade, leading to appropriate activation of NLRP3. Furthermore, NOD1, NOD2, TLR2, and TLR9 agonists also enhance NLRP3 expression. *L. salivarius* exerts its anti-inflammatory effect by producing IL10 *via* regulation of NOD2 (92, 93). Probiotics modulate systemic and local immune responses of the host in a strain-specific manner by the expression of PAMPS, such as flagellin, lipopolysaccharides, CpG-DNA, and other surface proteins. PAMPs are recognized by PRRs expressed on numerous immune and epithelial cells. TLRs, C-lectin type receptors, and NLRs are the best studied PRRs. PRRs have broad specificity and their limited number can recognize a wide range of PAMPs. Interaction between PAMPs and PRRs results in the activation of multiple molecular signaling cascades that generate a specific cellular response against the encountered microbes.

#### Probiotics and Regulation of the NF-κB Pathway

The NF-κB pathway is involved in many pathological conditions and controls the expression of many (~150) pro-inflammatory and anti-inflammatory cytokines genes. These genes are extensively involved in both adaptive and innate immune responses. NF-κB is found in nearly all types of cells (94, 95). Many probiotics regulate the activation of the NF-κB pathway. *L. casei* inhibits *Shigella fexneri*-induced activation of the NF-κB pathway (96). *L. rhamnosus* and *Lactobacillus helveticus* downregulate the Th1 pro-inflammatory response and improve Th2 response during *Citrobacter rodentium* infection (97). *Bifdobacterium lactis* inhibits IκBα degradation during colitis (98). Some researchers have claimed that dietary yeast downregulates TLR2, NF-κβ p65, MyD88, IL8, and IL1β (99). *L. reuteri, L. casei*, and *L. paracasei* show anti-inflammatory characteristics *via* NF-κB pathway regulation; for example, *L. reuteri* decreases the expression of inflammatory mRNA cytokines production and increases anti-inflammatory cytokines production, and also improves the production of apoptosis-inhibiting proteins to improve cell survival and its immune response. *L. reuteri* do this by interfering the ubiquitination of IκB and nuclear translocation of p65 (NF-κB subunit), respectively (100–102). *L. casei* and *L. paracasei* hinder the production of pro-inflammatory cytokines by inhibiting the phosphorylation of IκBα and nuclear translocation of p65, and also reverse the degradation of IκBα (103, 104). Similar inhibitory effects on the NF- κB pathway have been shown by *L. plantarum* and *L. brevis*. *L. plantarum* inhibits NF-κB-activating factors by decreasing the binding activity of NF-κB (105), while *L. brevis* prevents interleukin 1 receptor associated kinase 1 (IRAK1) and AKT phosphorylation (106). *Bifdobacterium infantis* and *Streptococcus salivarius* also reduce NF-κB activation (101).

Besides these probiotics have several other mechanisms of action related to antifungal, antibacterial, antiviral, antiparasitic, antiallergic, anti-cancerous, antidiabetic, amelioration of the cardiovascular system, the reproductive system, and the central nervous system which has been briefly discussed in the relevant section.

## Immune Regulation-Based Therapeutic Application of Probiotics During Infectious Diseases

Probiotics have a wide range of applications covering numerous non-infectious and infectious diseases, including bacterial, viral, parasitic, fungal, and many other non-infectious diseases. They exert anti-pathogenic effects by modulating both the innate and adaptive immune responses of the host.

### Bacterial Diseases

Due to the several disadvantages associated with the preventive use of antibiotics, strict controls have been introduced to prohibit or reduce their use during the treatment of bacterial diseases. In the last three decades, the dietary application of feed additives has been attracting attention as a replacement for antibiotics. Probiotics have been among the most effective feed additives for the control or treatment of bacterial diseases (5). Immune modulatory therapies with probiotics for some selected pathogens are briefly discussed below (Table 2).

**Table 2:** Probiotic therapies during bacterial diseases.

**Probiotics**	**Target bacteria**	**Study models**	**Mechanism of action**	**Effects**	**References**
*L. rhamnosus* S1K3	*S*. Typhimurium	Caco-2 cells, mice	↑ Claudin-1 ↑ sIgA, sIgA secreting cells Maintain IL-4, IL-12 protein level ↓ TGFβ	↑ Barrier integrity ↓*Salmonella* count Improves health status	(107)
Multistrain formula consisting of different *Lactobacilli*	*S*. Typhimurium	Chicken	↓ IFN-γ production	↓*Salmonella* complications ↑ Recovery rate	(47)
*L. plantarum* LPZ01	*S*. Typhimurium	Chicken	↓ IFN-γ production Regulate miRNA	↓*Salmonella* load and associated complications	(108)
*L. casei* DBN023	*S. pullorum*	Chicken	↓ TNF-α and IFN-γ ↑ IL10	↑ Villi height ↑ Muscle thickness ↑ Intestinal immune functions ↓ Mortality ↓ Pathological changes ↓ Inflammation	(58)
*L. casei* CRL 431	*S*. Typhimurium	Mice	↑ IL10	↓*Salmonella* associated complications	(82)
*S. cerevisiae* strain 905	*S*. Typhimurium	Mice	↑ IgA, IgM in serum ↑ Kupffer cells in liver ↓ IL-6, TNF-α, and IFN-γ	↓*Salmonella* load in Peyer's patches, spleen, mesenteric lymph nodes, liver ↓ Mortality	(109–111)
*S. boulardii*	*S*. Typhimurium	T84 cells	↓ NF-kB, MAPKs ERK1/2, p38, and JNK activation ↓ IL-8	↓*Salmonella* associated complications	(112)
*L. gasseri* Kx110A1	*H. pylori*	THP-1 cells	↓ TNF-α, IL6	↓*Salmonella* associated complications	(113)
*L. fermentum* UCO-979C	*H. pylori*	AGS cells	↓ IL8, IL1β, MCP-1	↓*H. pylori* induced gastric inflammation	(114)
*L. acidophilus* and *L. rhamnosus*	*H. pylori*	AGS cells	↓ NF-κB and MAPK activation ↓ IL8, IL6, MAP-2, IL1β, TNF-α.	↓*H. pylori* induced gastric inflammation	(115–117)
*L. bulgaricus* NQ2508	*H. pylori*	GES-1 cells	↓ TLR4 expression ↓ NF-κB activation ↓ IL8	↓*H. pylori* induced gastric inflammation	(118)
*L. rhamnosus* GG	*H. pylori*	AGS and Caco-2 cells	↓ Gastrin-17 ↓ IL8 and TNF-α	↓*H. pylori* induced gastric inflammation and ulceration	(119)
*L. paracasei* 06TCa19	*H. pylori*	MKN45 cells	↓ NF-κB and p38 MAPK activation ↓ IL-8 and RANTES	↓*H. pylori* induced gastric inflammation and ulceration	(120)
*S. boulardii*	Clostridial infection	BALB/c mice	↑ IgA, IgG, IgM	↓ Clostridial infection severity	(121)
*S. boulardii*	Clostridial infection	Mice	Inhibits the *Clostridium* toxins A-induced ERK1/2 and JNK/SAPK signaling pathways	↓ Clostridial infection severity	(122)
*S. boulardii*	Clostridial infection	Rat	Degrades Clostridial toxins by its protease action ↓ Binding of toxins to host cell	↓ Clostridial infection severity	(123)
*L. casei* BL23	*S. aureus*	Bovine mammary epithelial cells	↓ IL8, IL6, TNF-α, IL1β, and IL1α	↓ Inflammation of the mammary glands	(124)
*B. subtilis* DS991 EPS	*S. aureus*	C57BL/6J mice	↓ Pro-inflammatory cytokines, chemokines and T-cell activation	↓ Inflammation	(125)
*L. salivarius* BGHO1	*L. monocytogenes*	Rats	↑ CD14, TNF-α, IL1β ↓*Listeria* toxins	↑ Protection against *Listeria monocytogenes*	(126)
*L. delbrueckii* UFV-H2b20	*L. monocytogenes*	Mice	↑ TNF-α and IFN-γ Stimulates macrophages to increase bacterial killing	↑ Lifespan ↓ Bacterial load from liver and spleen ↓ Liver immunopathology	(127)
Heat-killed *Enterococcus faecium* BGPAS1-3 cell wall protein	*L. monocytogenes*	Caco-2 cells	↑ TGF-β and claudin production ↑ TLR4 expression ↓ TLR2 expression	↓*Listeria monocytogenes* infection	(128)
*Enterococcus faecium* JWS 833	*L. monocytogenes*	Mice and peritoneal mouse macrophages	↑ TNF-α, IL1β, Nitric oxide (NO)	↓*Listeria monocytogenes* complications	(129)
*L. fermentum* MTCC 5898	*E. coli*	Mice	↑ IFN-γ, TFNα, MCP-1 ↑ IgA, IgG1 ↑ Antioxidant enzymes activity ↓ IL-4 and IL-10	↓*E. coli* load in liver, spleen, intestine, and peritoneal fluids	(168)
*L. rhamnosus* MTCC 5897	*E. coli*	Mice	↑ IgA, IgG ↑ Antioxidant enzymes activity	↓*E. coli* load in liver, spleen	(167)
*L. rhamnosus* (LR: MTCC-5897)	*E. coli*	Caco-2 cells	↑ Claudin-1, Occludin, ZO-1, Cingulin	↓ Hyperpermeability Maintains barrier integrity	(170)
*L. fermentum* (LF: MTCC-5898)	*E. coli*	Caco-2 cells	↑ Claudin-1, Occludin, ZO-1, Cingulin	↓ Hyperpermeability Maintains barrier integrity	(57)
*L. rhamnosus* ACTT 7469	*E. coli*	pig	↓ TLR4 ↓ TNF-α, IL8	↓*E. coli* associated inflammation	(130)
*L. plantarum* B1	*E. coli*	chickens	↓ TLR4 expression ↓ IL2, IL4, IFN-γ ↑ Mucosal antibodies (IgA)	↓*E. coli* associated inflammation	(131, 132)
*L. jensenii* TL2937	*E. coli*	PIE cells	↓ IRAK-M, BCL3, TOLLIP, A20	↓*E. coli* associated inflammation	(133)
*L*. amylovorus DSM 1669	*E. coli*	Caco-2 cells and pig explant	Modulates Tollip and IRAK-M ↓ TLR4 expression ↓ phosphorylation of the IKKα, IKKβ, IκBα, and NF-κB subunit p65 ↓ IL-1β and IL8 production ↑ Hsp72 and Hsp90	↓*E. coli* associated inflammation	(134)
*L. delbrueckii* TUA 4408	*E. coli*	PIE cells	↓ MAPK and NF-κB activation	↓*E. coli* associated inflammation	(135)
*L. rhamnosus* ATCC 7469	*E. coli*	IPEC-J2 cell model	↑ ZO-1 and Occludin ↓*TLR4* and *NOD2* mRNA expression	Maintain epithelial barrier ↓*E. coli* associated deleterious effects	(136)

#### Salmonella Infection

Probiotics may be used as alternatives to the prophylactic use of drugs for the control and prevention of salmonellosis (137). *Salmonella* causes a foodborne disease in both animals and humans with high morbidity (93.8 million human infections) and mortality (155,000 deaths) worldwide annually (138–142). After attachment and internalization into the lamina propria, *Salmonella* induces an inflammatory response, e.g., release of pro-inflammatory cytokines, followed by inflammation, ulceration, diarrhea, and destruction of the mucosa (143). Persistent infection is established due to the ability of *Salmonella* to evade the host immune system (144). The persistence of infection is further aided by virulent factors of *Salmonella* that are responsible for the clonal deletion of CD^+^ T cells (145).

When administered in adequate amounts, probiotics have the ability to modulate the expression of immune-related cytokines, including interleukins IL4, IL6, IL12, IFN-γ, and IL1β in lymphoid cells during *Salmonella* infection (47, 107, 108, 142, 146). *L. rhamnosus* S1K3 maintains IL-4 and IL-12 protein levels and reduces TGFβ during the late stage of *Salmonella enterica* serovar Typhimurium (*S*. Typhimurium) NCDC infection in mice and also increases the level of IgA secreting cells in lamina propria, IgA in serum, and secretory IgA level in intestinal fluids during *S*. Typhimurium NCDC infection in mice. This probiotic also reduces the *S*. Typhimurium NCDC count in feces, prevents its further spread in the liver, spleen, and intestine of mice, and improves overall health. Furthermore, in an *in vitro* study on Caco-2 cells, *L. rhamnosus* S1K3 improves the tight junction proteins (occludin and claudin-1) (107). The production of IFN-γ, a pro-inflammatory cytokine, is induced by *Salmonella*. IFN-γ delays recovery from intestinal inflammation, boosts inflammatory mediators [TNF, ILβ, inducible nitric oxide synthase (iNOS)], and hampers IL22- and lectin REGIIIβ-mediated antimicrobial defense (147). Probiotics beneficially regulate the immune response of the host and suppress the expression of pro-inflammatory cytokines and subsequent inflammation. IFN-γ is suppressed by the anti-inflammatory action of probiotics, greatly reducing the severity of *Salmonella* infection. During salmonellosis, immune players, macrophages, and monocytes secrete IL6, which serves as a pro-inflammatory cytokine and its expression levels are reduced by *Lactobacillus* spp. for the effective and rapid prevention of *Salmonella* infection in broiler chickens (47). A study conducted by Chen et al. showed that *L. plantarum* (LPZ01) reduces *S*. Typhimurium load, IFN-γ expression, TNF-α level, and associated inflammation in broiler chickens by regulating the expression of certain miRNAs involved in immune regulation and inflammatory responses (108). Supplementations with some probiotics increase the activation of B cells and antibody production by increasing IL10 expression. The latter is an important immunoregulatory and anti-inflammatory cytokine involved in antibody production during *Salmonella* infection. *L. casei* (DBN023) improves, regulates, and enhances intestinal immune functions, while cytokines balance and reverse the detrimental effects of *Salmonella pullorum*, characterized by higher levels of anti-inflammatory cytokines (IL10) and lower levels of pro-inflammatory cytokines (TNF-α, IFN-γ, and IL17). During prophylactic feeding of probiotics in chicken infected by *Salmonella pullorum, L. casei* (DBN023) increases villi height and muscle thickness and reduces *Salmonella pullorum*-associated mortality and pathological changes in intestinal epithelial tissues (58). *L. casei* CRL 431 also increases the expression of IL10 to reduce the severity of *S*. Typhimurium infection in BALB/c mice (82). In this manner, probiotics improve the host immune response by hampering the overexpression of inflammatory cytokines, as well as increasing the expression of anti-inflammatory cytokines and production of anti-*Salmonella* antibodies to blunt the severity of *Salmonella* infection.

Some yeasts are also used as immunobiotics and are effective in reducing *Salmonella* infection. The study by Martins et al. shows that *Saccharomyces cerevisiae strain* 905 (S. *cerevisiae* 905) protects and reduces the mortality of mice, orally challenged by *Salmonella* Typhimurium (109), and also significantly reduces the translocation of *S*. Typhimurium to the liver of gnotobiotic mice, and to other organs (Peyer's patches, the spleen, the mesenteric lymph nodes, and the liver) of the conventional mice. The same author in another study shows that this strain increases the number of Kupffer cells in the liver and induces a higher level of secretory IgA in the intestinal contents and IgA and IgM in the serum of mice (110). Furthermore, this strain reduces pro-inflammatory cytokines (IL-6, TNF-α, and IFN-γ) levels and modulates activation of MAPK (p38 and JNK, but not ERK1/2), NF-κB and activator protein-1, signaling pathways which are involved in transcriptional activation of pro-inflammatory mediator during *Salmonella* infection (111). Another yeast strain *S. boulardii* reduces *S*. Typhimurium induced IL-8 production in T84 cells by exerting its inhibitory effects on *S*. Typhimurium induced activation of the MAPKs ERK1/2, p38, and JNK as well as on activation of NF-kB (112). *S. boulardii* possesses the capability to bind with *S*. Typhimurium leading to reduced organ translocation of this pathogen, which results in decreased activation of MAPK (p38, JNK, and ERK1/2), phospho-IkB, p65-RelA, phospho-jun, and c-fos in the colon and signal pathways, involved in the activation of inflammation, induced by *S*. Typhimurium kB (148). Therefore, yeast can survive in host GIT, colonize there, reduce the pathogenic load from the host, and can modulate the immune response of their hosts toward a beneficial pattern.

A series of studies show that short-chain fatty acids (SCFAs) exert diverse beneficial effects on the health of the host gut and body (e.g., anti-inflammatory effects, prevention of histone deacetylases, and suppression of NF-κB resulting in IL1β downregulation), and play a vital role in maintaining intestinal homeostasis. Many probiotics possess regulatory properties for SCFA and can directly or indirectly increase their production. *L. acidophilus* reduces *S*. Typhimurium-induced inflammation directly by increasing the production of SCFA and indirectly by increasing that of other SCFA-producing gut bacteria (149). Moreover, *L. acidophilus* balances *Salmonella*-induced dysbiosis in infected mice (150).

Other probiotics have also shown beneficial effects on the prevention of *Salmonella* infection and inhibit the pathogenesis of *Salmonella* at initial steps. *L. plantarum* (MTCC5690) improves the intestinal defense through modulation of TLR2 and TLR4, and prevents the colonization and further spread of *Salmonella* in mice (151). Similarly, *E. faecium* (PXN33) in combination with *L. salivarius* (59) also inhibits *Salmonella Enteritidis* colonization in the GIT of poultry (152). Supplementation of probiotics greatly reduced the severity of *Salmonella* infection by their immunomodulatory mechanisms of action. As probiotics decrease the expression of inflammatory cytokines and increase the antibody production and anti-inflammatory cytokine expression during salmonellosis, supplementation can improve the overall health of the host.

#### Helicobacter Pylori Infection

*Helicobacter pylori*, a Gram-negative and spiral-shaped pathogenic bacterium, resides in >50% of the population worldwide and causes different diseases characterized by prominent gastric inflammation which is associated with gastric ulcers. The mechanism of *H. pylori-*induced inflammation includes chemokine (IL8)-mediated infiltration of neutrophils, increased RANTES level, and *H pylori* urease-induced degradation of NF-κB inhibitor (IκBα) (115, 120, 153–155). *H. pylori* can survive inside macrophages, arrest phagocytosis, and induce their apoptosis by preventing nitric oxide (NO) production. Furthermore, *H. pylori* stimulates macrophages to secret TNF-α and IL6, which are associated with gastric inflammation, by expressing the TNF-α-converting enzyme17 (ADAM17). ADAM17 is a crucial enzyme for the maturation and functioning of TNF-α and IL6. *L. gasseri* Kx110A1 inhibits these pro-inflammatory cytokines from *H. pylori*-infected THP-1 cells by inhibiting the expression of the *H. pylori* ADAM17 enzyme (113). *L. fermentum* UCO-979C regulates the immune response of host macrophages (HumanTHP-1 cell line) and human gastric epithelial cells (AGS cell line) by stimulating them to secrete specific cytokines and chemokines. Moreover, it significantly increases the secretion of inflammatory cytokines (IL6, TNF-α, and IL1β) in both AGS and macrophages, and the secretion of IL10, IFN-γ, and IL12p70 only in macrophages prior to *H. pylori* challenge. In contrast, it decreases the levels of *H. pylori-*induced inflammatory cytokines [IL8, IL1β, monocyte chemoattractant protein-1 (MCP-1), and IL6] in AGS, and those of TNF-α in both AGS and macrophages. Thus, prior to infection, treatment with *L*. *fermentum* UCO-979C increases inflammatory cytokines to counter future infections. In contrast, during infection, *L*. *fermentum* UCO-979C treatment lessens the over-activated immune response of host cells, as also shown by Garcia-Castillo et al. (114). The study reported that *L. fermentum* has the ability to decrease *H. pylori-*associated inflammation by improving TGF-β production in the AGS cell line. TGF-β inhibits NF-κB activation by upregulating the levels of IκBα. Notably, *H. pylori* infection impedes this TGF-β-associated signaling pathway by inducing SMAD7 expression to promote inflammation.

Similar to *L*. *fermentum, L. acidophilus*, and *L. rhamnosus* also regulate the immune response of host cells and decrease their pro-inflammatory immune response against *H. pylori*. As shown by their anti-inflammatory effects in AGS cells, in which both probiotics greatly reduced the CagA-induced expression of IL8 by inhibiting its translocation into host cells. CagA is an *H. pylori* virulent factor responsible for inflammation by the degradation of cytoplasmic IκBα and increasing translocation of NF-κB into the nucleus (116, 156, 157). Moreover, *L. acidophilus* activates Th1 response to counter *H. pylori* infection, suppresses *H. pylori*-induced SMAD7 expression as well as the activation of the NF-κB and MAPK signaling pathways, and decreases subsequent inflammatory response (production of IL8, IL6, MAP-2, IL1β, TNF-α, and granulocyte-colony stimulating factor) during *H. pylori* infection (115, 117). *L. bulgaricus* NQ2508 also shows similar anti-inflammatory effects by reducing *H. pylori-*induced IκBα degradation and subsequent IL8 production in the human gastric epithelial cell line-1 (GES-1). It may also secrete some soluble proteins which exert inhibitory effects on TLR4 and inhibit its activation by *H. pylori*. Moreover, it blocks subsequent signaling pathways toward NF-κB activation and its delivery to the nucleus for the transcription of pro-inflammatory cytokines (118). As mentioned above, gastric ulcers and cancer are prominent complications of *H. pylori* infection. They mainly arise due to the over-immune response of host cells and the subsequent production of inflammatory cytokines, which are involved in gastric ulceration. Many probiotics reduce these complications by regulating the *H. pylori*-disrupted immune response. *L. rhamnosus* GG reduces gastric ulceration and cancer induced by *H. pylori via* the IL8/TNF-α/Gastrin-17 pathway. *H. pylori* upregulates Gastrin-17 by increasing the levels of IL8 and TNF-α, which in turn upregulate Gastrin-17. Gastrin-17 typically causes gastric cancer, whereas IL8 and TNF-α cause inflammation and apoptosis leading to ulceration of the stomach. *L. rhamnosus* GG shows significant immunobiotic properties with anti-inflammatory effects and attenuates Gastrin-17 levels by suppressing the expression of IL8 and TNF-α (119, 158–161). Similarly, *L. paracasei* may ameliorate *H. pylori*-induced gastric inflammation by regulating the immune response of host cells. *L. paracasei* 06TCa19 inhibits *H. pylori* CagA-induced p38 and IκBα phosphorylation and increases the levels of these NF-κB inhibitors in MKN45 cells. This results in inhibition of the transcription of the inflammatory chemokine genes (120). Numerous other probiotics are extensively used to ameliorate *H. pylori*-induced complications with the aim to regulate the immune system of the host (162, 163).

#### Escherichia Coli Infection

*Escherichia coli* causes different problems for humans and animals. Enterotoxigenic *E. coli* (ETEC) causes diarrhea in piglets and other species by secreting heat-labile and heat-stable toxins. Through a complex mechanism, these toxins activate the chloride channel (cystic fibrosis transmembrane channel) resulting in diarrhea. The *E. coli* causing postweaning diarrhea mostly carries F4 (K88) fimbriae (164). F4^+^ ETEC increases the expression of membrane and cytoplasmic-associated receptors (TLRs and NLRs), which are involved in the NF-κB signaling pathway and subsequent production of pro-inflammatory cytokines (IL8 and TNF-α) leading to inflammation (130, 164, 165).

Probiotics greatly reduce the expression of these pro-inflammatory cytokines by reducing the interaction of *E. coli* with membrane receptors. *L. rhamnosus* ACTT 7469 weakens the *E. coli*-induced expression of TLR4, TNF-α, and IL8 at the protein and mRNA levels in piglets. Furthermore, *L. rhamnosus* increases the expression of TLR2, TLR9, and NLR in the case of *E. coli* infection in piglets, which results in decreased intestinal inflammation (130). As mentioned above, TLR2 and TLR9 are involved in the anti-inflammatory effects of many probiotics.

Similar anti-inflammatory effects have also been shown by supplementation of *L. plantarum* B1, which reduces *E. coli*-induced inflammation in broiler chickens by decreasing the expression of TLR4 and the levels of cytokines (IL2, IL4, and IFN-γ) involved in inflammation. *L. plantarum* also increases the levels of mucosal antibodies (IgA) (131, 132). Hence, probiotics (mainly, the *Lactobacillus* species), regulate the immune response in a beneficial manner by decreasing the expression of membrane receptors (TLR4) involved in inflammation associated with pathogens. On the other hand, probiotics increase the expression of membrane receptors (TLR2, TLR9) involved in the reduction of pathogen-induced inflammation. Like, *Lactobacillus jensenii* TL2937 in porcine intestinal epithelial cells decreases the expression of TLRs by increasing the negative regulators [IRAK-M, BCL3, toll interacting protein (TOLLIP), and A20] of these receptors and reduces the *E. coli* induced inflammation (133). Another study also reported similar anti-inflammatory effects of other probiotics (*Lactobacillus amylovorus* DSM 1669 and *L. delbrueckii* TUA 4408), including inhibition of ETEC-induced activation of the NF-κB and MAPK pathways *via* negative regulation of TLRs, which results in a decrease of pro-inflammatory cytokines (IL1, IL6, IL-1β, and IL8) and an increase of anti-inflammatory cytokine (IL10) in pig explant, caco-2, and porcine intestinal epithelial cells (134, 135). Amdekar et al. also demonstrated that *Lactobacillus* species play a key protective role against *E. coli*-induced urinary tract infection, and clearance of pathogens by regulating the expression of TLRs (TLR2 and TLR4) and subsequent production of anti-inflammatory cytokines (166). Probiotics induce the expression of different kinds of cytokines involved in host immune response during pathogenic infection by regulating the expression of TLR and their intracellular signaling pathways. They increase the expression of anti-inflammatory cytokines and reduce the inflammatory response of host cells during infection. *L. amylovorus* shows protective and anti-inflammatory effects in pig explants and caco-2 cells against *E. coli* infection and decreases *E. coli-*mediated inflammation by increasing the levels of TLR4 negative regulators (IRAK-M and TOLLIP) and decreasing those of extracellular heat shock proteins (HSP90 and HSP72), which are crucial for TLR4 functioning. This effect leads to inhibition of the *E. coli*-induced increase in the levels of TLR4 and MyD88, phosphorylation of IκBα, IκB kinase α (IKKα), IKKβ, and NF-κB subunit p65, as well as the overproduction of inflammatory cytokines (IL8 and IL1β) (134). Treatment with *L. rhamnosus* ATCC 7469 decreases TLR4 and NOD2 mRNA expression during ETEC infection in IPEC-J2 cell model and reduces the associated inflammatory response of the host. Notably, ETEC induced higher mRNA expression of these membrane and cytoplasmic receptors that lead to the transcription of inflammatory genes *via* the NF-κB pathway (136).

Some probiotics improve the immune status of aging mice to increase their resistance against infection. The study of Sharma et al. on mice reported that *L. rhamnosus* MTCC 5897 feeding alleviates the imbalance of Th1/Th2 immune response and also increases the activity of antioxidant enzymes (catalase, glutathione peroxidase, and superoxide dismutase) and reduces *E. coli* load in the liver, spleen, and intestines by increasing the level of *E. coli* specific antibodies (IgA and IgG) (167). Similarly, *L. fermentum* MTCC 5898 feeding in aged mice increases their protection against *E. coli* infection by increasing the IgA and IgG1 levels and inflammatory proteins and reduces the *E. coli* load in the intestines, liver, spleen, and peritoneal fluids (168). Other lactobacilli improve the *E. coli* disturbed intestinal barrier function as, *E. coli* significantly decreases the intestinal permeability by decreasing the level of tight junction proteins (Occludin, ZO-1, cingulin-1, claudin-1, etc.) as observed by Bhat et al. in Caco-2 cells (169). *L. rhamnosus* (LR: MTCC-5897) improves these tight junction proteins and significantly reduces the *E. coli* induced hyperpermeability in Caco-2 cells (170). Similar effects were also observed by *L. fermentum* (LF: MTCC-5898) treatment during *E. coli* infection in Caco-2 cells in which *L. fermentum* (LF: MTCC-5898) improves the barrier integrity by reducing *E. coli* induced lower mRNA expression of Occludin, ZO-1, cingulin-1, and claudin-1 (57).

Thus, probiotics positively regulate the immune response of host cells at various steps through different mechanisms of action and protect the host from ETEC-induced deleterious effects.

#### Clostridial Infection

Clostridial species are rod-shaped, Gram-positive toxins and spore-producing bacteria. *Clostridium difficile* is linked to a wide range of clinical problems (171) and produces many toxins (e.g., cytotoxins and enterotoxins), which cause diarrhea (172). It mainly produces the exotoxins TcdA and TcdB with a size of ~300 kDa. When it binds apically with epithelial gut cells, TcdA causes tight junction interruption and also facilitates the binding of TcdB toxins to the basal lamina. TcdB causes an increase in vascular permeability, release of neurotensin, induction of pro-inflammatory cytokines, fluid secretion, and eventually diarrhea (173).

Probiotics may subside the detrimental effects of clostridial infection by modulating the innate (mucus, lysozymes, and alpha defensin production, and modulation of membrane receptors such as TLRs and NLRs) and adaptive (production of immunoglobulins, anti-inflammatory cytokines, antigen uptake, and modulation of antigen-presenting cells) immune responses and cell signaling pathways (NF-κB and MAPK) of the host (173, 174). *S. boulardii* is a type of yeast that may be used as a probiotic against clostridial toxins. It increases the production of antibodies (IgA, IgG, and IgM) acting as adjuvant in BALB/c mice (121) and has numerous other mechanisms of action associated with immune regulation. It inhibits the activation of the NF-κB and MAPK signaling pathways, and pro-inflammatory cytokine (IL8) production induced by *C. difficile* toxin A in human colonic epithelial cells (NCM460). This toxin activates the extracellular signal-regulated kinase 1/2 (ERK1/2) and stress-activated protein kinases (SAPK)/Jun amino-terminal kinases (JNK) (JNK/SAPK) pathways, resulting in the transcription of pro-inflammatory cytokine (IL8) genes and leading to inflammation. *S. boulardii* inhibits the *Clostridium* toxins A-induced ERK1/2 and JNK/SAPK signaling pathways in mice (122). Furthermore, it degrades *C. difficile* toxins by its protease action and decreases the binding of toxins to host cell (rat ileum) receptors (123).

#### Staphylococcus Infection

*Staphylococcus* is a major cause of bovine contagious mastitis and persistent infection in bovine mammary epithelial cells in animals. *Via* upregulation of TLR2 and TLR4, *Staphylococcus aureus* (*S. aureus*) increases the secretion of basic fibroblast growth factor and TGF-β1 through activation of the NF-κB pathway by inhibiting NF-κB inhibitors in bovine mammary epithelial cells (175). Many probiotics are used to treat and control *Staphylococcus* infection. Probiotic *L. casei* (BL23) significantly reduces inflammation of the mammary glands during *S. aureus* infection by suppressing the expression of *S. aureus*-induced pro-inflammatory cytokines (IL8, IL6, TNF-α, IL1β, and IL1α). This results in potent anti-inflammatory effects against *S. aureus* infection in bovine mammary epithelial cells (124). *Bacillus subtilis* has shown protective effects against *S. aureus* infection in mice, by activating macrophages, limiting systemic inflammation induced by *S. aureus*, and decreasing the pathogen load. *Bacillus subtilis*-secreted exopolysaccharides (EPS) have an immunomodulatory function, producing hybrid macrophages (having the functions of both M1 and M2) with anti-inflammatory and bactericidal phagocytic characteristics. These hybrid macrophages limit *S. aureus*—induced T-cell activation and kill *S. aureus* by increasing the levels of reactive oxygen species and decreasing the levels of pro-inflammatory cytokines and chemokines [chemokine (C-C motif) ligand 2 (CCL2), CCL3, CCL4, TNF] (125). Paynich et al. (176) study on mice showed that *Bacillus subtilis*-exopolysaccharides induces anti-inflammatory macrophages (M2), which inhibit T-cell (CD4^+^ and CD8^+^) activation by secreting TGF-β and PD-L1 molecules. These molecules have inhibitory effects on CD4^+^ and CD8^+^ cells, showing a significant anti-inflammatory property in T cell-dependent immune reaction (176). In this way, probiotics beneficially regulate the immune response of host cells; they activate immune cells to kill *S. aureus* and decrease pathogen-associated inflammation by limiting the overexpression of inflammatory cytokines from pathogen-activated immune cells.

#### Listeria Monocytogenes Infection

*Listeria monocytogenes* causes several infections, including maternal-fetal infection, septicemic pneumonia, pleural infection (177), foodborne diseases with a 20–30% mortality rate (178), and neurolisteriosis leading to meningitis and encephalitis (179). Several probiotics (mostly *Lactobacilli* species) are used to protect the host against *L. monocytogenes* infection. *L. salivarius* (BGHO1) therapies against *L. monocytogenes* exert protective effects by modulating the adaptive and innate immune responses during *L. monocytogenes* infection in rats. BGHO1 increases the mRNA expression of CD14, TNF-α, and IL1β and decreases listeriolysin (*Listeria* toxins) in the intestinal tissues. In mesenteric lymph nodes, BGHO1 co-administered with *L. monocytogenes* enhances CD69 and OX-62 mRNA expression (126). *L. delbrueckii* induces the production of TNF-α and IFN-γ, which stimulates the macrophages to kill *L. monocytogenes*. Mice infected with *L. monocytogenes* which received *L. delbrueckii* UFV-H2b20 have a longer lifespan, less liver immunopathology, and less bacterial load in the spleen and liver (127). These probiotics stimulate macrophages by inducing the expression of specific cytokines to increase their bactericidal activities and decrease the level of toxins, as well as assist the host in eliminating pathogens from their body and accelerate recovery.

Heat-killed *E. faecium* BGPAS1-3 cell wall protein, which is resistant to high temperature, has shown protective and strong anti-listeria activity. It stimulates Caco-2 cells to increase TGF-β production. TGF-β exerts protective effects on epithelial tight junctions by upregulating the expression of claudin (128). These innate immunomodulatory effects are achieved by modulation of the MyD88-dependent TLR2 and TLR4 pathways in intestinal cells against *Listeria* infection. *L. monocytogenes* induces TLR2 and suppresses the expression of TLR4 mRNA in Caco-2 cells. Heat-killed BGPAS1-3 decreases the expression of TLR2 mRNA in Caco-2 cells. In contrast, the expression of TLR4 mRNA in Caco-2 cells is increased by both heat-killed and live BGPAS1-3 before and after *L. monocytogenes* infection, respectively. Furthermore, heat-killed or live BGPAS1-3 has inhibitory effects on the expression of IL8 in uninfected and infected *L. monocytogenes* Caco-2 cells (180). Heat-killed and live probiotics, as well as their cellular components, can regulate the immune response of the host through interaction with TLRs, increase the protective innate immune response, and decrease the inflammatory response of host cells. Cho et al. showed the protective and immunomodulatory effects of heat-killed and live *E. faecium* JWS 833 using a *L. monocytogenes* mice model and peritoneal mouse macrophages, respectively. Both heat-killed and live JWS833 show immunomodulatory properties. When administered orally, live JWS833 increases the levels of serum cytokines (TNF-α and IL1β) and NO against *L. monocytogenes* in mice. Heat-killed JWS833 stimulates the macrophages to produce TNF-α, NO, and IL1β (129). Probiotics have diverse immunomodulatory functions, assisting the host to counter pathogenic infections.

### Viral Diseases and Probiotics

The threat of viral illness has recently increased significantly due to the changes in the environment (e.g., anthropogenic climate change and increased global movement of passengers and cargo). Viral infections cause variable morbidity and mortality with a detrimental effect on community well-being and cause widespread economic losses. Respiratory Syndrome Coronavirus 2 (SARS-CoV-2), which infected millions of people worldwide during the 2019–2020 pandemic is a good example of this global economic loss (181). Thus, finding alternative and effective strategies to prevent viral infections and reducing the morbidity and mortality of viral infections is critical (Table 3). Nevertheless, many vaccines and antiviral drugs aiming to be effective in infections are available, but a major challenge is the new viral strains that appeared after mutations, particularly in RNA viruses. It is wise to have some alternative strategies that could be used as supplemental or preventive remedies. To reduce the severity of viral infections and their numbers, a balanced diet including nutrients or food additives that boost and potentiate immune system response, is a beneficial alternative measure. The use of probiotics is one of the dietary approaches used in recent years to increase immunity and decrease the risk of infections (213). Many probiotics (mainly *Lactobacilli* species) are used for the prevention or treatment of viral illnesses. In addition, to alter the crosstalk between gut bacteria and the mucosal immune system, probiotics have many other immune modulatory and non-immune functions to combat viral incursion. The application of probiotics for the control and prevention of clinically important viral diseases is briefly discussed below.

**Table 3:** Probiotics therapies during viral diseases.

**Probiotics**	**Target viruses**	**Study models**	**Mechanism of action**	**Effects**	**References**
*Bifidobacterium infantis* (MCC12)	Rotavirus	PIE cells	↓ IL-8, ↓ A20, ↑ IRF3, ↑ IFN, ↑ ISGs	↓ Virus replication ↑ Infected cells apoptosis	(182, 183)
*Bifidobacterium breve* (MCC1274)	Rotavirus	PIE cells	↓ IL-8, ↓ A20, ↑ IRF3, ↑ IFN	↓ Virus replication ↑ Infected cells apoptosis	(182, 183)
*Bifidobacterium lactis* Bb12	Rotavirus	Pig rotavirus model	↑ T cells subset (CD3^+^, CD4^+^) ↑ Vaccine efficacy	↓ Virus load	(184)
*Bifidobacterium adolescentis* (DSM 20083)	Rotavirus	MA104 cells	Interact with virus protein (NSP4)	↓ Diarrhea	(185, 186)
*L. rhamnosus* GG (strain ATCC 53103)	Rotavirus	Pig rotavirus model	↑ T cells subset (CD3^+^, CD4^+^) ↑ Vaccine efficacy	↓ Virus load	(184)
*L. casei* (Lafti L26-DSL)	Rotavirus	MA104 cells	Interact with virus protein (NSP4)	↓ Diarrhea	(185, 186)
*L. acidophilus* and *L. reuteri*	Rotavirus	Pig model	↑ Intestinal IgM and IgG ↑ Serum IgM titers ↑ Total intestinal IgA secreting cell response	↓ Virus load	(187)
*Lactobacillus delbrueckii* ssp. bulgaricus OLL1073R-1 fermented yogurt	Influenza virus	96 volunteers	Affect IgA levels in saliva	Help to prevent influenza infection	(188)
*L. paracasei*	Influenza virus	Mice	↑ IL1α and IL1β before infection ↑ Recruite immune cells before infection ↑ IL10 after infection	↓ Viral load ↓ Morbidity ↓ Mortality	(189)
*L. casei* DK128	Influenza virus	Mice	↑ IgG1, IgG2a, ↓ IL6 and TNF-α ↑ Monocytes	↓ Inflammation ↑ Host survival rate	(190)
*L. plantarum* (O6CC2)	Influenza virus	Mice	↑ IFN-a and Th1 cytokines	↓ Infection severity	(191, 192)
*L. paracasei* CNCM I-1518	Influenza viruses	Mice	↑ Early recruitment of IL-1α, IL-1β Recruit immune cells before infection	↑ Protection against virus	(189)
*L. plantarum* (AYA)	Influenza virus	Mice	↑ IgA	↓ Infection severity	(193)
*L. GG* and *L. johnsonii* (NCC 533)	Influenza virus	Mice	↑ IgA, IFN-g	↓ Mortality ↓ Morbidity ↓ Virus titer ↓ Cell death	(194)
*Bifidobacterium longum* BB536	Influenza virus	Mice	↑ Activities of neutrophils and NK cells.	↓ Weight loss ↓ Virus replication ↓ Infection severity	(195, 196)
*L. plantarum* (137)	Influenza virus	Mice	↑ IFN-β	↓ Infection severity	(197)
*L. delbrueckii* ssp. bulgaricus OLL1073R-1 fermented yogurt	Influenza virus	96 volunteers	Affect IgA levels in saliva	Help to prevent influenza infection	(188)
*L. acidophilus* NCFM and *Bifidobacterium animalis* subsp. *lactis* Bi-07	Influenza virus like symptoms	326 children	–	↓ Fever incidence (53.0%) ↓ Coughing incidence (41.4%) ↓ Rhinorrhea incidence (28.2%)	(198)
Recombinant *L. plantarum*	Corona viruses (TGEV and PEDV)	IPEC-J2	↑ ISGs (OASL, ISG15, Mx1) ↑ B^+^IgA^+^, IgG ↑ IFN-γ	↓ Infection severity	(199, 200)
*L. casei* ATCC39392 vaccine	TGEV	Pig model	↑ Antibodies ↑ IL17	↓ Infection severity	(201)
*L. plantarum* Probio-38 and *L. salivarius* Probio-37	TGEV	ST cell line	Inhibit virus	↓ Infection severity	(202)
cell-free supernatants of *L. plantarum* 22F, 25F, and 31F, live *L. plantarum* (22F, 25F)	PEDV	Vero cells	Antiviral activity	↓ Infection severity	(203)
Mixture of different Lactobacilli and Bifidobacteria	HIV	Clinical trial on 8 human positive patients	↑ Serotonin in blood ↓ Tryptophan in plasma		(204)
*L. rhamnsosus* GR-1 and *L. reuteri* RC-14	HIV	Clinical trial of 65 confirmed women	–	Improved life quality of women	(205)
*L. plantarum* 299v	HIV	Clinical trial of 14 children	Stabilize CD4^+^ T cells numbers	↓ Inflammation	(206)
*S. boulardii* CNCM I-745	HSV-1	Mice	↑ Anti-inflammatory cytokines ↓ pro-inflammatory cytokines	↓ Gastrointestinal dysfunctioning	(207)
*L. rhamnosus* BMX 54	Human papillomavirus (HPV)	Clinical trial of 117 women	–	Favors recreation of vaginal balance, may be useful to control HPV infection	(208)
*Bifidobacterium bifidum*	HPV	Mice	↑ IL2 ↑ IFN-γ	↓ Virus complication, prevent tumor growth	(209)
*L. reuteri* RC-14 and *L. rhamnosus* GR-1	HPV	Clinical trial of 180 women	–	↓ Abnormal cervical smear rate, no effect on virus clearance	(210)
*L. rhamnosus* PTCC 1637 and *E. coli* PTCC 25923	Herpes simplex virus-1	African green monkey kidney cells	↑ Viability of macrophages Competitive adhesion with cells	↑ Virus elimination Antiviral effects	(211)
*Enterococcus faecalis* FK-23	Hepatitis C virus	*In vitro* trial of 39 positive patients	↓ Alanine transferase	Improve health	(212)
*Bifidobacterium bifidum* 2-2, *Bifidobacterium*. *bifidum* 3-9, *L. gasseri* TMC0356, *L. casei* TMC0409*, L. rhamnosus* LA-2 *L. rhamnosus* (LGG), Streptococcus thermophilus TMC1543	Enteric common infectious diseases	Bovine intestinal epithelial cell line	↑ TLR3 activation ↑ IFN β	↑ Protection against enteric viruses	(213)
*L. fermentum* PCC, *L. casei* 431 and *L. paracasei*	Upper respiratory tract viruses and influenza viruses	Clinical trial of 136 volunteers	↑ Serum IFN-γ ↑ Intestinal IgA	↓ Symptoms of flue and respiratory tract infection incidence	(214)
*L. plantarum* DR7	Upper respiratory tract virus's infection	Clinical trial of 209 adults	↑ IL-4, IL-10, CD44, CD117 ↓ IFN-γ, TNFα, CD4, CD8	↓ Nasal symptoms and frequency of URTI ↓ Oxidative stress ↓ Plasma peroxidation	(215)
*Bifidobacterium bifidum* G9-1 (BBG9-1)	Rotavirus	BALB/c mice	Induced mucosal protective factors	Improve lesion and diarrhea	(216)
*L. helveticus* R0052 and *L. rhamnosus* R0011	Rotavirus, Adenovirus, Norovirus	Clinical trial of children (816)	–	No beneficial effects	(217)
*L. paracasei* N1115	Upper respiratory tract viruses	274 clinical volunteers' trial	May stimulate T cell immunity	Protection against acute respiratory tract infection	(218)

#### Rotavirus

*Bifidobacterium infantis* (MCC12) and *Bifidobacterium breve* (MCC1274) modulate immune response during human rotavirus infection in the porcine intestinal epithelial cell line. Both species are able to blunt IL8 production and increase IFN production by increasing the activation of interferon regulatory factor 3 (IRF3) through the suppression of A20 (a zinc-finger protein with negative effects on IRF3 activation) (182). These probiotics activate various interferon-stimulated genes (ISGs), including RNase L (2′-5′ oligoadenuylate dependent endoribonulecase) and myxovirus resistance protein A (MxA) (183). MxA decreases virus replication by binding with virus nucleoproteins (219). RNase L has antiviral activity and lessens viral replication through the elimination of infected cells by inducing apoptosis and IFN amplification by activating RLRs (220, 221). RLRs are intracellular PRRs involved in virus recognition. *L. rhamnosus* GG (strain ATCC 53103) and *B. lactis* Bb12 enhance the efficacy of human attenuated rotavirus vaccine (AttHRV) during rotavirus infection in gnotobiotic human rotavirus pig model, by increasing T-cells subset (CD3^+^, CD4^+^) in intestinal tissues and T-cells subset (CD3^+^, CD8^+)^ in the blood and spleen. Further, the severity of diarrhea and virus load was also less in vaccinated pigs receiving ATCC 53103 and Bb12 as compared to only vaccinated pigs (184). Similarly, *S. boulardii* and several *Bifidobacterium* and *Lactobacillus* species have anti-rotaviral effects, mitigate the severity and duration of diarrhea, viral shedding, and incidence of infections associated with rotavirus, and modulate the immune response of the host (222–226) *Lactobacillus* species and *Bifidobacterium* in combination with some prebiotics (human milk oligosaccharide, short-chain galactooligosaccharides, and long-chain fructooligosaccharides) show antiviral response. *L. casei* (Lafti L26-DSL) and *Bifidobacterium adolescentis* (DSM 20083) reduced the infectivity of virus in MA104 cells (embryonic Rhesus monkey kidney cells) by interacting with virus protein (NSP4). NSP4 has been characterized as virus toxin and is associated with diarrhea in host (185, 186). *L. rhamnosus* (strain GG) and Gram-negative *E. coli* Nissle (*EcN*) decrease human rotaviral complications by modulating the immune response and interacting with rotavirus. In the pig rotavirus model, *EcN* and *L. rhamnosus* GG induced higher total IgA levels in the intestine and serum post- and pre-human rotavirus challenge, respectively, and reduced viral shedding. *EcN* can regulate the expression of cytokines (IL6 and IL10) and bind with rotavirus protein 4 to reduce rotavirus attachment to the host cells (227, 228). In the rotavirus gnotobiotic pig model, *Lactobacilli* species (*L. acidophilus* and *L. reuteri*) significantly increased total intestinal IgM and IgG and serum IgM titers and total intestinal IgA secreting cell responses (187). Furthermore, Azevedo et al. (229) demonstrated that these probiotics (*L. acidophilus* and *L. reuteri*) significantly increased Th1 and Th2 cytokines in human rotavirus infected pigs, and also help in maintaining immunological homeostasis during human rotavirus infection by regulating the production of TGF-β. Different probiotics have anti-rotavirus activities involving various immunomodulatory mechanisms. *Bifidobacterium* stimulates ISGs and lowers various pro-inflammatory cytokines, while *Lactobacillu*s increases anti-rotavirus antibodies and reduces rotavirus-associated complications.

#### Influenza Virus

A randomized controlled trial on 96 elderly people showed that a yogurt fermented with *L. delbrueckii ssp. bulgaricus* OLL1073R-1 (1073R-1-yogurt) affected the level of influenza A H3N2 bound IgA levels in saliva (188). *L. acidophilus* NCFM and *Bifidobacterium animalis* subsp. *lactis* Bi-07 reduce the incidence of coughing (41.4%), rhinorrhea (28.2%), and fever (53%) in a double blind placebo controlled study on 326 children during the winter season (198). Different clinical trial studies on children, elderly people, adults, and animals compiled by Lehtoranta et al. (230) shows that probiotic administration reduced the risk respiratory viruses including influenza viruses. In mice, *L. paracasei* showed anti-influenza effects and beneficially modulated the immune response against influenza infection, while reducing the viral load, morbidity, and mortality. *L. paracasei* increases the levels of pro-inflammatory cytokines (IL1α and IL1β) and recruitment of immune cells before infection. This accelerates viral clearance and reduces the levels of inflammatory cytokines [macrophage inflammatory protein-1α (MIP1α), IFN-γ, MCP-1, and MIP1β] after influenza infection. Moreover, *L. paracasei* has shown anti-inflammatory characteristics at the late stage of infection by increasing the levels of IL10 (189). Heat-killed *L. casei* DK128 shows similar anti-inflammatory effects against influenza infection by decreasing influenza virus-induced pro-inflammatory cytokines (IL6 and TNF-α), monocytes, and activated NK cells in the lungs of mice, thereby preventing pulmonary inflammation. Furthermore, DK128 increases the levels of antibodies (IgG1 and IgG2a) against the influenza virus at an earlier time point and provides cross-immunity against secondary heterosubtypic influenza infection with improved health and survival rate in mice (190). *L. plantarum* (O6CC2) beneficially modulates the host immune response during influenza infection in mice by increasing the production of IFN-α and Th1 cytokines (IL12 and IFN-γ) as well as the expression of Th1 cytokine receptors which potentiate NK cell activity at the early stage of influenza infection in mice. Of note, NK cells are an important line of defense during this early phase (191, 192). At the late stage of infection, *L. plantarum* (O6CC2) decreases IL6 and TNF-α production to control influenza-mediated inflammation. Furthermore, O6CC2 decreases neutrophil and macrophage infiltration to overcome the inflammatory response to influenza infection (231). *L. plantarum* (AYA) has shown protective immunological effects against influenza virus infection by increasing production of mouse mucosal IgA (193). *L. GG* and *L. johnsonii* (NCC 533) are also associated with increased IgA production (232, 233). *B. longum* (MM-2) has shown anti-influenza activity by enhancing the innate immunity through increases in the expression of NK cell activator genes (IFN-g, IL2, IL12, IL18) activities. This probiotic reduces mortality, morbidity, virus titer, cell death, virus-induced inflammation, and the expression of mRNA for pro-inflammatory cytokines (IL6, TNF-α, IL1β, MIP2, and MCP-1) in mice infected with influenza virus (194). Similar immune regulatory and anti-influenza effects of *Bifidobacterium* have been observed by other researchers. *B. longum* BB536 enhances the activities of neutrophils and NK cells, reduces fever in human beings (195), reduces IL6 and IFN-γ at the late stage of infection, and prevents body weight loss and virus replication in the lungs of mice infected with the influenza virus (196). *L. plantarum* (137) induces higher type-1 interferon (IFN-β) levels in the serum of mice at the early stage of influenza infection (197). Notably, innate immunity of type-1 interferon is involved in countering viral infection at the early stage (234). In the case of the influenza virus infection, gut microbiota have preventive effects and modulate type I IFNs (235). These IFNs are involved in innate immunity during viral infection with antiviral activities, as well as the degradation and inhibition of viral nucleic acids and viral gene expression, respectively (236, 237). These studies showed that various probiotics show anti-influenza activities along with immunoregulatory effects during infection.

#### Coronavirus

Coronavirus disease 2019 (COVID-19) was officially declared as a pandemic by WHO on March 11, 2020 (238). SARS-CoV-2 was first identified in Wuhan city (China) in December 2019 (239) in patients with pneumonia and rapidly spread to 216 countries (240, 241). Coronaviruses (CoVs) belong to the family Coronaviridae and genus coronavirus order Nidovirales and subfamilies: Alphacoronavirus, Betacoronavirus, Gammacoronavirus, and Deltacoronavirus (242). Subfamilies alphacoronavirus and Betacoronavirus originate from mammals mainly bats, and Gammacoronavirus and Deltacoronavirus subfamilies originate from pigs and birds (243). In SARS-CoVs virion envelop, there are three main structural proteins—protein S (Spike), protein M (membrane), and protein E (envelop). Protein S (Spike) facilitates the SARS-CoVs adherence and fusion (52). All CoVs are positive sense, single stranded RNA, and pleomorphic viruses with typical crown shape peplomers of 27–32 kb and 80–160 nM size (239, 244). Genomic structure analysis showed that the viruses belong to β-coronavirus including MERS-CoV and SARS-CoV with high mutation rates because of RNA dependent DNA polymerase transcription error (242), which is also the main target of drug discovery (245). Pathogenesis of SARS-CoV-2 includes binding of its spikes proteins (S) to Angiotensin-Converting-Enzyme-2, which are highly expressed in lungs as well as in esophagus and enterocytes in the colon and ileum, to get entry into the cells for infection (246). TMPRSS2 is a protein, which helps the “S” proteins of SARS-CoV-2 to get entry into cells, is also highly expressed in absorbent enterocytes (247). Clinical signs of COVID-19 disease are different ranging from asymptomatic to non-specific flu and severe pneumonia, Middle Eastern respiratory syndrome (MERS) (248), and life-threatening consequences like acute respiratory distress syndrome and different organ failure. It can also affect neurological, gastrointestinal, and hepatic systems (249). According to data from Wuhan city in China, 14% of the infected cases were severe, 4% died, and 5% needed intensive care (250).

In spite of the different measurements including hygienic improvement, screening, and social distancing, COVID-19 is rapidly spreading and progressing worldwide (22, 240), while the search for effective drugs and vaccine therapies is underway. Scientists are battling against the time needed to develop a vaccine, but it is hard to make an efficient and safe product as rapidly as the virus is spreading (251). Thus far, there are no effective drugs available for SARS-CoV-2. However, according to genomic structure analysis and its similarity with SARS and MERS, certain drugs (e.g., lopinavir, ritonavir, and nitazoxanide) may be applicable (252). At the same time, several studies have compiled alternative data related to general viruses management and treatment (253–258) including nutritional supplements like vitamins and some other immune boosts medicine (259). Some *in silico* data are in favor of probiotics use for the treatment of COVID 19 as data indicate that probiotics derived molecules like lactococcin Gb (*L. lactis*), subtilisin (Bacillus amyloliquefaciens), sakacin P (*L. sakei*) may inactivate “S” glycoprotein and its receptors molecules i.e., Angiotensin-Converting-Enzyme-2 (260). Similarly, several other studies have published their data regarding the use of probiotics for the general management of viral diseases as it is indicated by some clinical evidence that some kinds of probiotics are helpful in preventing bacterial and viral infections like respiratory tract infections, sepsis, and gastroenteritis. Viruses account for over 90% of upper RTIs as etiological agents. Many studies have recorded the positive effect of probiotics on the protection of upper respiratory tract infections. Reduced risk of getting upper respiratory tract infections in probiotic supplementations was recorded in a meta-analysis study of 12 randomized control trials involving 3,720 children and adults. It was observed in 479 adults of a randomized, double-blind, placebo-controlled intervention study that *B. bifidum* MF 20/5, *L. gasseri* PA 16/8, and *B. longum* SP 07/3 along with mineral and vitamins reduced the duration of fever and common cold (22). *Streptococcus salivarius* strain K12 may possibly reduce the severity of COVID-19 complications by its ability to maintain stable upper respiratory tract microbiota. As advanced studies have shown that lung microbiota have an important role in the homeostasis of immune responses (261), and its dysbiosis makes the patient more vulnerable to viral infections. In the case of COVID-19, a significant difference in lung microbiota has been observed in patients with COVID-19 and normal persons (262). Probiotic consumption triggers pro-and anti-inflammatory cytokines production to clear the viral infection, reduce the cell damage in the lungs, and improve the levels of T cells, B cells, NK cells, and type 1 interferons in the immune system of the lungs, and it may help to prevent COVID-19 complications (261).

Probiotics and recombinant probiotics with antiviral effects are effectively used to combat and minimize the detrimental effects of other coronaviruses, such as alphacoronaviruses—particularly transmissible gastroenteritis virus (TGEV) and porcine epidemic diarrhea virus (PEDV)—which cause substantial economic losses in the pork meat industry. Recombinant *L. plantarum* inhibits TGEV and PEDV infections in the IPEC-J2 cell line by enhancing ISGs (OASL, ISG15, and Mx1) which have strong antiviral effects (199). Recombinant *L. plantarum* (containing the surface S antigen of TGEV) elicits an immune response characterized by higher numbers of activated DC cells, B^+^IgA^+^ cells, secretory IgA (sIgA), serum IgG, IFN-γ, and IL4 which help the host to combat TGEV (200). Similar effects were observed by Jiang and colleague who reported that a recombinant *L. casei* ATCC39392 vaccine modulates the immune response against TGEV infection, induces IL4, mucosal (IgA), and systemic (Ghosh and Higgins) antibodies, and polarized Th2 immune response with enhanced the expression of IL17 against TGEV in a pig model (201). Similarly, immune protective effects with the elicitation of sIgA and IgG production against PEDV have also been shown by a *L. casei*-based vaccine, consisting of a DC-targeting peptide attached to the PEDV core antigen (263). Antibiotics and porcine bile-resistant *L. plantarum* Probio-38 and *L. salivarius* Probio-37 have shown antiviral effects *in vitro* ST cell line and inhibit TGE coronavirus without cytotoxic effects (202). Another study shows that cell-free supernatants of different LAB (*L. plantarum* 22F, 25F, and 31F) and live *L. plantarum* (22F, 25F) have anti PEDV activity with any cytotoxic effects on Vero cells (203). *E. faecium* has protective effects against enteropathogenic coronavirus TGEV and hinders the virus entry into cells by interacting with cell surface molecules, reducing viral structural proteins, and inducing antiviral NO (264, 265). Furthermore, *E. faecium* stimulates an antiviral response by increasing the expression of IL8 and IL6 mRNA (266), which contribute to the immune regulation against many other enteric pathogens (267). Studies show that *E. faecium* (probio-63) and *E. faecalis* (KCTC 10700BP) suppress coronavirus growth, responsible for porcine epidemic diarrhea (268, 269). These findings indicate that probiotics have antiviral effects, and stimulate the immune response of the host against viruses. Many probiotics enhance vaccine efficacy; some probiotics inhibit virus entry into cells and also stimulate the production of different cytokines during viral infection.

### Probiotics and Parasitic Diseases

Probiotics are widely applicable to the treatment and prevention of parasitic infections (Table 4). Oral administration of *L. rhamnosus* MTCC 1423 during Giardia infection in mice modulates both cellular and humoral immune responses, enhances sIgA, IgA^+^ cells, CD4^+^ T lymphocytes, and anti-inflammatory cytokine IL10, and decreases pro-inflammatory cytokine IFN-γ (277). *E. faecium* SF 68 stimulates an anti-giardia immune response, increases CD4^+^ T cells and the production of anti-giardia antibodies (intestinal IgA and serum IgG), and reduces the parasitic load (278). *Lactobacillus* and *S. boulardii* also have positive effects in the treatment of giardiasis, minimizing interaction between the host and pathogen, reducing parasite load, and modulating the immune response of the host. *L. johnsonii* La1 (NCC533) reduces active trophozoite of Giardia intestinalis strain WB and infection duration in *Meriones unguiculatus* (286). Recombinant *L. plantarum* NC8 (containing *Eimeria tenella* protein) induced a higher percentage of a T-cell subset (CD3^+^, CD4^+^, and CD8^+^) and antibody levels, provided protection against *E. tenella* infection in chickens, and reduced lesion, cecum damage, and oocyst shedding (270). *L. salivarius, L. johnsonii*, and *S. cerevisiae* provided protection against *Eimeria* infection in chickens; reduced oocyst count, improved weight gain and FCR, and stimulated the immune response with higher antibodies (IgM and IgG) titer and lymphoproliferative response (271). Pender et al. revealed that chickens receiving supplementation of commercially available probiotics; Primalac W/S (*L. acidophilus, L. casei, E. faecium*, and *B. bifidium*) showed lower mortality, higher body weight, and fewer *Eimeria maxima*-, *Eimeria tenella*-, and *Eimeria acervulina*-induced lesions; however, there was no effect on the immune response (272). Lactic acid from *L. acidophillus* stimulates the host immune response during *Cryptosporidium* infection, increasing the number of lymphocytes, levels of complement proteins (C3, C4), and antibodies (IgM, IgG), as well as reducing oocyst shedding from infected rabbits (273). *L. casei*, Bifidium bacteria, and *E. faecalis* exert protective effects during *Cryptosporidium parvum* infection and greatly reduce parasite load and oocyst shedding from the intestine of infected mice (287–289). In contrast, Oliveira and Widmer demonstrated that some commercially available probiotics enhanced the severity of cryptosporidia infection by altering the intestinal environment in favor of *C. parvum* proliferation (290). *Bifidobacterium animalis subspecies lactis* strain Bb12 stimulates local immune response during *Ascaris suum* infection in juvenile pigs and production of anti-parasite antibodies (IgA in serum and IgG1 and IgG2 in ileal fluid) and glucose uptake (274). Similarly, *L. rhamnosus* modulates the expression of TNF-α, TLR9, IFN-γ, and IL10 gene, which results in decrease in eosinophil action and allergic skin reaction induced by *Ascaris suum* in the pig model (275, 276). Many probiotics are effective against schistosomiasis; *Zymomonas mobilis* stimulates immune response and provides 61% protection during schistosomiasis (291). *L. plantarum, L. reuteri, L. casei*, and *L. acidophilus* stimulate IgM antibodies against *Schistosoma mansoni* infection in mice (279). *L. sporogenes* reduces schistosomiasis cytokine-induced chromosomal aberration in mice (280). During trichinellosis (*Trichinella spiralis* infection in mice), *L. plantarum* increases the levels of IFN-γ and reduces larval count (281). *L. fermentum, E. faecium*, and *Enterococcus durans* enhance the activity of phagocytes during *Trichinella spiralis* infection in mice (282). *L. casei* induces IgA and IgG during *T. spiralis* infection in mice (283, 292). In trichuriasis mice model, *L. rhamnosus* (JB-1) increases IL10 and mucus-secreting goblet cells, resulting in the faster removal of larvae (284). *E. faecalis* CECT7121 (*Ef* 7121) and *S. boulardii* are associated with larvicidal activity and high production of IL12 and IFN-γ, respectively, during *Toxocara canis* infection in mice (285, 293). Different probiotics have different mechanisms of action during parasitic infections. They reduce complications, regulate cytokine production, and facilitate the production of anti-parasitic antibodies. However, it has been shown that some probiotics enhance the parasitic infection as indicated in the study by Dea-Ayuela and colleague on mice in which they reported that *L. casei* decreases cytokines (IFN-γ, TNF-α, IL-4, and Il-13) and antibodies (fecal IgA) against *Trichuris muris*, increasing the susceptibility of *T. muris* infection. This *L. casei* associated increased susceptibility to infection may be related to deactivation of TNF-α dependent Th2 effector responses against *T. muris* due to the strong inhibitory effect of *L. casei* on this cytokine (294).

**Table 4:** Probiotics therapies during parasitic diseases.

**Probiotics**	**Parasites**	**Study models**	**Mechanism of action**	**Effects**	**References**
Recombinant *L. plantarum* NC8	*Eimeria tenella*	Chicken	↑ CD3^+^, CD4^+^, CD8^+^ ↑ IgA, IgM and IgG	↓ Lesion ↓ Cecum damage ↓ Oocyst shedding ↓ Inflammation	(270)
*L. salivarius, L. johnsonii*, and *S. cerevisiae*	*Eimeria tenella, Eimeria maxima, Eimeria necatrix*	Chicken	–	↓ Oocyst count ↑ Weight gain ↑ FCR	(271)
Primalac W/S (*L. acidophilus, L. casei, Enterococcus faecium*, and *Bifidobacterium bifidium*)	*Eimeria maxima, Eimeria tenella*, and *Eimeria acervulina*	Chicken	–	↓ Lesion	(272)
*L. acidophillus* lactic acid	*Cryptosporidium parvum* oocysts	Rabbit	↑ Complement proteins (C3, C4) ↑ Lymphocytes ↑ IgM and IgG	↓ Parasitic load	(273)
*Bifidobacterium animalis*	*Ascaris suum*	Juvenile pigs	↑ IgA in serum ↑ IgG1 and IgG2 in ileal fluid	↓ Parasitic complications	(274)
*L. rhamnosus*	*Ascaris suum*	Pigs	↑ TLR9 expression ↑ TNF-α, IFN-γ, and IL10	↓ Parasitic allergic complications	(275, 276)
*L. rhamnosus*	*Giardia intestinalis* (Portland strain I)	BALB/c mice	↑ sIgA, IgA^+^ cells, CD4^+^ ↑ T lymphocytes ↑ IL10 ↓ IFN-γ	↓ Giardia infection severityRestore intestinal morphology	(277)
*Enterococcus faecium* SF68	*Giardia intestinalis* H7 (ATCC 50581)	Mice	↑ Intestinal IgA ↑ Serum IgG ↑ CD4^+^ T cells	↓ Parasitic load	(278)
*L. plantarum, L. reuteri, L. casei*, and *L. acidophilus*	*Schistosoma mansoni*	Mice	↑ IgM ↓ AST, LDH, and gGT	↓ Parasitic complications ↓ Spleen and liver weight	(279)
*L. sporogenes*	*Schistosoma mansoni*	Mice	↓ Schistosomiasis cytokine-induced chromosomal aberration	↓ Chromosomal aberration	(280)
*L. plantarum*	*Trichinella spiralis*	Mice	↑ Serum IFN-γ	↓ Larval count ↓ Inflammation	(281)
*L. fermentum, Enterococcus faecium, Enterococcus durans*	*Trichinella spiralis*	Mice	↑ Phagocytic activity of leukocytes	↑ Protection	(282)
*L. casei*	*Trichinella spiralis*	Mice	↑ IgA and IgG	↑ Protection	(283)
*L. rhamnosus* (JB-1)	*Trichuris muris*	Mice	↑ IL10 ↑ Mucus-secreting goblet cells	↑ Larval removal	(284)
*S. boulardii*	*Toxocara canis*	Mice	↑ IL12 and IFN-γ	↑ Protection	(285)

## Probiotics Therapies in Non-infectious Disorders

Probiotics improve the central nervous system and mental function with beneficial effects reported in anxiety, Alzheimer's disease, depression, schizophrenia, and autism (295). In an autism spectrum disorder mice model, an *L. reuteri* diet led to a behavioral improvement in an oxytocin-dependent manner (296). Probiotics can alter the composition of gut microbiota (297), which in turn acts on the gut–brain axis by secreting neuroactive substances (298) and significantly influences and regulates cerebrovascular diseases, neurodegeneration, and mental dysfunction (299). *B. infantis* reduces stress by increasing the levels of tryptophan in plasma, decreasing the levels of serotonin in the frontal cortex, and regulating the hypothalamic–pituitary–adrenal axis. *L. rhamnosus* JB-1 decreases the expression of gamma aminobutyric acid receptor and corticosterone levels in mice, which are induced during stress (300–302). Moreover, *B. longum, L. helveticus*, and *L. plantarum* reduce anxiety (303). *L. fermentum* NCIMB can produce ferulic acid, which is a strong antioxidant that can stimulate the proliferation of the nervous system stem cells and be used to treat neurodegenerative disorder, diabetes, and obesity. In mice, feeding ferulic acid ameliorates Alzheimer's disease symptoms, oxidative stress, and neuroinflammation (65). Thus, probiotics have positive effects on brain function, by affecting the functions of the nervous system as well as some related hormones and their receptors (Table 5). However, a detailed study of the effects of probiotics on the nervous system is needed to support the currently available evidence.

**Table 5:** Probiotics therapies in non-infectious diseases.

**Probiotics**	**Disease**	**Study models**	**Major finding**	**References**
*L. rhamnosus* (MTCC5897) fermented milk (PFM)	Allergy	Mice	↑ IgA^+^ cells in small intestine ↑ Goblet cells number ↓ Ovalbumin-specific antibodies (IgE, IgG, IgG1) ↓ Ratio of IgE/IgG2a and IgG1/IgG2a ↓ Allergic symptoms	(304)
*L. plantarum* 06CC2	Allergy	Mice	↓ Ovalbumin-specific IgE ↓ Total IgE ↑ Antiallergic IL-4 and IFN-γ ↓ Allergic symptoms	(305)
*Bifidobacterium infantis* CGMCC313-2	Allergy	Mice	↓ IL4, IL13 ↓ IgE, IgG1 ↓ Allergic symptoms	(306)
*Enterococcus faecalis* FK-23	Allergy	Mice	↓ IL-17 ↓ CD4^+^ cells ↓ TH17 development ↓ Allergic symptoms	(307)
*Staphylococcus succinus* 14BME20	Allergy	Mice	↓ IgE level in serum ↓ Inflammatory cells flux into lungs ↑ CD4^+^CD25^+^Foxp3^+^ regulatory T (Treg) ↑ DCs ↑ IL-10	(308)
*Clostridium butyricum* CGMCC0313	Allergy	Mice	↓β-lactoglobulin-mediated intestinal anaphylaxis Inverts the imbalance between Th1/Th2 and Th17/Treg cells ↑ forkhead box P3 (FOXP3) Treg cells ↑ TGF-β and IL10	(309)
*L. acidophilus* KLDS 1.0738	Allergy	Mice	↓ Inflammatory cells ↓ IgE production ↓ IL6 levels ↓ Th17 response ↑ Treg cells, CD25, FOXP3 ↓ TGF-β	(310)
*L. fermentum* MTCC: 5898-fermented milk	Cardiovascular	Mice	↓ TNF-α and IL-6 ↓ Coronary artery risk index ↓ Atherogenic index ↓ Triacylglycerols, low-density lipoprotein cholesterol, hepatic lipids ↓ Lipid peroxidation	(311)
*L. rhamnosus* MTCC: 5957 and *L. rhamnosus* MTCC: 5897	Cardiovascular	Wistar rat	↓ TNF-α and IL-6 ↓ hyperlipidemia ↓ Hepatic lipids ↓ Lipid peroxidation ↑ Antioxidant activities	(312)
*L. plantarum*	Cardiovascular	Meta-analysis of randomized controlled trials of 653 participants	↓ Diastolic and systolic blood pressure ↓ Total serum cholesterol ↓ Low-density lipoprotein cholesterol levels ↓ Atherosclerosis index ↓ Hepatocyte steatosis risk	(313, 314)
*L. fermentum* CECT5716 and *Bifidobacterium breve* CECT7263	Cardiovascular	Wistar Kyoto rats	↓ Hypertensions ↓ Endothelial dysfunctioning ↓ Increased blood pressure	(315)
*L. rhamnosus* GR-1 *L. plantarum* 299v	Cardiovascular	rats	↓ Risk of myocardial infarction Improve ventricular function ↓ Infarct size ↓ levels of leptin	(316, 317)
*L. rhamnosus* MTCC: 5957, *L. rhamnosus* MTCC: 5897, and *L. fermentum* MTCC: 5898	Diabetes	Wistar rat	Improve glucose metabolism (fasting blood glucose, glycated hemoglobin, serum insulin) Improve serum inflammation status (TNF-α and IL-6) Improve serum lipid profile	(318)
*L. plantarum, L. helveticus, L. lactis, L. pentosus, L. paracasei, L. paracasei sbusp.tolerans, L. mucosae, L. rhamnosus, L. harbinensis, L. hilgardii, Issatchenkia orientalis, Candida ethanolica, Kluyveromyces marxianus*, and *Pichia membranifaciens*	Diabetes	db/db mice and C57BL/KS	Prevent pancreatic cell apoptosis *via* upregulation of the PI3K/AKT pathway and increase GATA like protein 1 (GLP1) production. GLP1 induces insulin secretion by upregulating the G protein-coupled receptor 43/41 (GPR43/41), proconvertase 1/3 and proglucagon activity	(319)
*L. fermentum* NCIMB	CNS	Mice	↑ Ferulic acid ↓ Alzheimer's disease symptoms ↓ Oxidative stress and neuroinflammation	(65)
*L. reuteri*	CNS	Mice	Behavioral improvement	(296)
*L. rhamnosus* JB-1	CNS	Mice	↓ Gamma aminobutyric acid receptor and corticosterone levels	(300)
*L. rhamnosus, E. faecium, L. acidophilus, Bifidobacterium bifidum*, and *Bifidobacterium longum*	Obesity	*In vivo* human trial	↓ Low density lipoprotein cholesterol ↓ Total cholesterol ↓ Oxidative stress	(320)
*Bifidobacterium bifidum* W23, *Bifidobacterium lactis* W51&W52, *L. lactis* W19&W58, *L. brevis* W63, *L. casei* W56, *L. acidophilus* W37, and *L. salivarius* W24	Obesity	*In vivo* human trial	↓ Homocysteine ↓ Triglyceride ↓ Total cholesterol ↓ TNF-α	(321)
*L. plantarum* CBT LP3, *Bifidobacterium breve* CBT BR3	Obesity	*In vivo* human trial	Reduced obesity marker	(320)
*L. fermentum* NCIMB 5221	Obesity	–	↑ Ferulic acid ↓ Obesity	(65)
*L. fermentum* NCIMB	Cancer	AGS, HeLa, MCF-7, and HT-29 cells	↓ Risk of cancer	(65)
*L. casei*	Cancer	Colonic epithelial cells and HT29 cells	↓ Adenoma atypia ↓ DNA damage	(322)
*L. acidophilus*	Cancer	Breast cancer mouse model	↓ Tumor growth	(323)

Different probiotics regulate obesity (323), which predisposes individuals to different diseases, such as non-alcoholic fatty liver diseases, cardiovascular diseases, diabetes, cancers, and some disorders related to the immune system (324). *L. plantarum* CBT LP3 and *B. breve* CBT BR3 reduce obesity related marker, and *L. rhamnosus, E. faecium, L. acidophilus, B. bifidum*, and *B. longum* decrease low-density lipoprotein cholesterol, total cholesterol and oxidative stress level in an *in vivo* human trial (320). *B. bifidum* W23, *B. lactis* W51&W52, *L. lactis* W19&W58, *L. brevis* W63, *L. casei* W56, *L. acidophilus* W37, and *L. salivarius* W24 regulate the obesity by decreasing triglyceride, total cholesterol, homocysteine, and TNF-α level in a randomized double-blind placebo-controlled trial on 50 women who were obese (321). Indigenous microbiota play a key role in obesity by harvesting energy for the host through different metabolic pathways. Probiotics change the composition of gut microbiota, thereby influencing obesity (12). Gut microbiota contribute to obesity *via* several potential mechanisms, such as lipogenesis, carbohydrate fermentation, and energy storage, and through numerous pathways (e.g., different hormones, metabolites, and neurotranmitters), which regulate energy balance and food intake (Table 5).

Probiotics also reduce the risk of cancer by different mechanisms of action, which include the exclusion of oncogenic bacteria, improvement of epithelial barrier function, increase of tumor cell death by apoptosis, production of immune-modulating metabolites (acetate, butyrate, propionate, conjugated linoleic acids, etc.), increase of cytokine production with an antitumor response, and TLR modulation. Butyrate regulates cell proliferation, differentiation, and apoptosis (325), it can stimulate anti-inflammatory cytokines and IL10 production and decrease the production of inflammatory cytokines *via* inhibition of NF-κB. Furthermore, butyrate regulates apoptosis-regulating proteins [CASP7, CASP3, BCL2 antagonist/killer (BAK), and BCL2], suppresses COX2 activity, stimulates the production of AMPs, and increases glutathione-S-transferase. These effects lead to downregulation or upregulation of genes related to the apoptosis, proliferation, and differentiation of cells (326, 327). Propionic acids and acetic acid have also shown anti-inflammatory activities by suppressing NF-κB activation and modulating the expression of pro-inflammatory genes (328). Some probiotics (*Lactobacilli, bifidobacteria*, and *streptococcus*) can produce conjugated linoleic acid, which has pro-apoptotic and anti-proliferative activities. This is achieved by increasing the expression of peroxisome proliferator-activated gamma receptor (PPARγ), which is involved in immune function and apoptosis. Some probiotics show their anti-cancerous activities *via* cation exchange between their peptidoglycan and the carcinogenic compound. Furthermore, probiotics decrease the COX2 enzyme-mediated production of prostaglandins, which increases the risk of colorectal cancer (329, 330). Probiotics can increase the production of immunoglobulins, such as IgA, generating an anti-inflammatory environment. IgA does not provoke activation of the complement system and acts as a barrier to reduce contact between the carcinogenic compound in the lumen and colonocytes, thereby reducing the risk of cancer (331). A prospective study involving 82,220 individuals showed that individuals who consume yogurt and sour milk are less susceptible to bladder cancer. An Italian cohort study on 45,000 volunteers of a 12-year follow up without comparative group, reports that yogurt consumption decrease in colorectal cancer (332). *L. casei* administration in humans for 4 years showed less recurrence of adenoma atypia, and probiotics with oligofructose-enriched inulin preparation reduce DNA damage in colonic epithelial cells and HT29 cells (322). Animal studies supported the beneficial effects of yogurt against genotoxic amines and cancer of the bladder and colon. In a breast cancer mice model, *L. acidophilus* isolated from yogurt promoted the proliferation of lymphocytes and decreases tumor growth (323) (Table 5). Hence, probiotics reduce the risk of cancer by different mechanisms. Some probiotics assist in excluding the oncogenic bacteria, while others inhibit inflammatory pathways and increase apoptosis of tumor cells. Furthermore, probiotics stimulate the production of immune-modulating metabolites involved in cell growth, proliferation, and apoptosis.

Many probiotics have beneficial effects on allergies (Table 5). *L. rhamnosus* (MTCC5897) fermented milk (PFM) feeding in newborn mice alleviates allergic symptoms by shifting Th2 to Th1 pathway by decreasing albumin specific antibodies (IgE, IgG, and IgG1), ratio of IgE/IgG2a and IgG1/IgG2a and IL-4, and by increasing IFN-γ, IgA^+^ cells, and goblet cells (304, 333). *Bifidobacteriales, Bacteroidales*, and *Lactobacillale*s in the gut affect the activities of inhaled allergens. *Bifidobacteriales* and *Lactobacillales* suppress allergen sensitization and are effective against allergic rhinitis (334). *B. infantis* CGMCC313-2 represses allergen-mediated inflammatory cells, IL4, IL13, IgE, IgG1, and blunt inflammation during allergy in mice model (306). *E. faecalis* FK-23 inhibits the development of Th17 cells in the intestine, spleen, and lungs of infected mice by inhibiting the expression of TGF-β and IL6 mRNA, thereby facilitating to reduce ovalbumin-induced allergic complication (307). *Staphylococcus succinus* 14BME20 has also shown antiallergic potential; it significantly decreases the influx of inflammatory cells into the lungs, suppresses airway hyperresponsiveness, and reduces the serum IgE and Th2 cells cytokines production in an ovalbumin mice model (308). *Clostridium butyricum* CGMCC0313 increases forkhead box P3 (FOXP3) Treg cells, TGF-β, and IL10, inverts the imbalance between Th1/Th2 and Th17/Treg cells, and reduces β-lactoglobulin-mediated intestinal anaphylaxis, thereby contributing to the reduction of the risk of allergy in mice (309). Orally administered *L. acidophilus* KLDS 1.0738 ameliorates allergic symptoms by increasing Treg cells, CD25, FOXP3, and TGF-β mRNA expression, and inhibiting inflammatory cells, IgE production, IL6 levels, and Th17 response in mice (310).

Many probiotics are used for the prevention and treatment of diabetes (Table 5). *L. rhamnosus* MTCC: 5957, *L. rhamnosus* MTCC: 5897 and *L. fermentum* MTCC: 5898 feeding Improves glucose metabolism (fasting blood glucose, serum insulin, and glycated hemoglobin), oxidative stress (glutathione peroxidase, superoxide dismutase, catalase activity, and thiobarbituric acid reactive substances,) serum inflammation status (TNF-α and IL-6) and serum lipid profile in diabetic rats, and also significantly reduces mRNA expression of gluconeogenesis related genes (pepck and g6pase) (318). *L. acidophilus* KLDS 1.0901 shows antidiabetic characteristics by reducing glycosylated hemoglobin, fasting blood glucose level, and increasing the level of glucagon-like peptide 1 in the serum of mice. Further, *L. acidophilus* KLDS 1.0901 increases glutathione peroxidase and superoxide dismutase activities and also increases the level of glutathione with the reduction of malondialdehyde level in mice serum (335). Similarly, *L. paracasei* 1F-20, *L. fermentum* F40-4, *Bifidobacterium animalis* subsp. *lactis* F1-7 also exhibit the potential to manage the diabetic problem as shown by the *in vitro* study of Zhang et al. (241) using CACO-2, STC-, RAW246.7, and HepG2 cells in which these probiotics increase glucagon-like peptide 1 and peptide YY hormones and decrease IL-6 and TNF-α levels (336).

Different species of other *Lactobacilli* and yeast strains act also as antidiabetic, preventing pancreatic cell apoptosis *via* upregulation of the PI3K/AKT pathway and increased GATA-like protein 1 (GLP1) production. GLP1-induced insulin secretion by upregulating the G protein-coupled receptor 43/41 (GPR43/41), proconvertase 1/3, and proglucagon activity in mice (319). GLP1 is an antidiabetic hormone involved in glucose homeostasis, and reduction of glucagon secretion and appetite (337–339). Many probiotics improve glucose metabolism (340) and inhibit NF-κB pathway overactivation. NF-κB is associated with diabetes and its inhibition leads to improvement in insulin sensitivity (94, 309). Probiotics reduce the risk of diabetes by regulating different cellular signaling pathways and the expression of sugar metabolism hormones.

Some probiotics improve sperm maturation; *L. casei* and *B. lactis* enhanced the maturation of sperm in diabetic rats and decreased their glucose levels (341). *L. rhamnosus* increased the mRNA expression of androgen receptors α and β, activin and progesterone receptor 1, serum follicle-stimulating hormone, luteinizing hormone, and testosterone. These effects were associated with improvement in spermatogenesis, sperm motility, and sperm production, along with a decrease in the percentage of immotile sperm (342, 343). *Bacillus amyloliquefaciens* has shown similar beneficial effects on semen density, live sperm, and overall quality in breeder chicken (344).

Probiotics are also widely applied to cardiovascular diseases; they significantly decrease hypertension, oxidative stress, blood pressure, inflammatory mediators, and cholesterol levels (311, 345–348). It is observed that cholesterol-enriched fed mice show significantly higher levels of serum triacylglycerols, total cholesterol, low-density lipoprotein cholesterol, atherogenic index, lipid peroxidation, coronary artery risk index, and IL-6 and TNF-α in the liver whereas significantly lower levels of catalase, anti-oxidative enzymes activities, glutathione peroxidase, and superoxide dismutase in the kidney and liver. Whereas, *L. fermentum* MTCC: 5898-fermented milk improves these adverse physiological conditions (311). Similarly, feeding of *L. rhamnosus* MTCC: 5957 and *L. rhamnosus* MTCC: 5897 maintains healthy liver and kidney conditions of Wistar rats by increasing antioxidant activities and by decreasing lipid peroxidation, diet-induced hypercholesterolemia in the feces, kidney, liver, and blood of the rats. These probiotics also reduce the expression of mRNA of the TNF α and IL-6 inflammatory markers (312).

*Lactobacillus plantarum* has shown beneficial effects during the meta-analysis of a randomized controlled trial of 653 participants having cardiovascular diseases, lower diastolic and systolic blood pressure (313), total serum cholesterol, low-density lipoprotein cholesterol levels, atherosclerosis index, and hepatocyte steatosis risk. Furthermore, *L. plantarum* decreases liver triglyceride and cholesterol, whereas it increases cholesterol in feces and excretion of bile acid (314). *In vivo* study of Robles-Vera et al. (315) showed that *L. fermentum* CECT5716 and *Bifidobacterium breve* CECT7263 feeding prevent the development of hypertension, endothelial dysfunctioning, and increase in blood pressure in rats (315). *L. rhamnosus* GR-1 and *L. plantarum* 299v reduce the risk of myocardial infarction, improve ventricular function, and reduce the infarct size by decreasing the levels of leptin in rats (316, 317). Probiotics also decreased the levels of toxic circulating metabolites (indoxyl-sulfate and p-cresyl sulfate) associated with cardiovascular diseases and reduced mortality in patients undergoing dialysis (349). Probiotics exert beneficial effects on cardiovascular diseases through different mechanisms of action (i.e., improving the ratio of low-density and high-density lipids, lowering cholesterol levels, improving endothelial function, and regulating the immune cells) (Table 5).

## Conclusions and Future Prospects

Due to increasing antibiotic-resistant bacteria and antibiotic side effects, the use of antibiotics as a feed supplementation is prohibited in many countries. China also bans the supplementation of growth-promoting antibiotics in animal feed since January 1, 2021. Probiotics are considered as a good alternative for antibiotics, providing an alternative treatment option. Probiotics are widely used in human aquaculture, livestock, and poultry to promote health and counteract enteric pathogens. Probiotics are widely used for the management and treatment of bacterial, viral, parasitic infections as well as non-infectious disorders like mental disorders, cancer, allergies, and metabolic disorders. Concerning their mechanisms of action, probiotics have immunomodulatory and many other mechanisms of action, and work in diverse ways to exert beneficial effects on their hosts, if applied properly. However, concerning the safety and efficacy of probiotics, recent screening techniques rely on the capacity of microbes to elicit cytokine production mostly through cell lines or *ex vivo* isolated residual immune cells, even though they do not reflect the phenotype of intestinal cells. Awareness of the capability and usage of probiotics to improve the microbiota equilibrium in the host gut, to serve as immunomodulators, growth promoters, and to inhibit pathogenic infections is crucial from a practical point of view. It will help to make more progress by investigating more expertise, knowledge, and research on the understanding of probiotics, their specific mechanism of action, and their complete applicability for the safety of the host. More importantly, the safety of probiotics during application should also be carefully considered and strictly evaluated in the future in case of the emergence and spread of antibiotic-resistant bacteria between hosts. Thus, high-throughput validation approaches, as well as comprehensive and credible clinical, *in vivo*, and *in vitro* research on probiotic administration are warranted to clearly illustrate the advantages and adverse effects of probiotics.

## Author Contributions

SC and LL designed, modified, and reviewed the manuscript. AR and GZ wrote the manuscript. All authors contributed to the article and approved the submitted version.

## Conflict of Interest

The authors declare that the research was conducted in the absence of any commercial or financial relationships that could be construed as a potential conflict of interest.

## References

[1] Dhama K Verma V Sawant P Tiwari R Vaid R Chauhan R. Applications of probiotics in poultry: enhancing immunity and beneficial effects on production performances and health: a review. J Immunol Immunopathol. (2011) 13:1–19.

[2] Santacroce L Charitos IA Bottalico L. A successful history: probiotics and their potential as antimicrobials. Expert Rev Anti Infect Ther. (2019) 17:635–45. 10.1080/14787210.2019.1645597 31318576

[3] Kopp-Hoolihan L. Prophylactic and therapeutic uses of probiotics: a review. J Am Diet Assoc. (2001) 101:229–41. 10.1016/S0002-8223(01)00060-8 11271697

[4] Zommiti M Feuilloley MG Connil N. Update of probiotics in human world: a nonstop source of benefactions till the end of time. Microorganisms. (2020) 8:1907. 10.3390/microorganisms8121907 33266303 PMC7760123

[5] Hoseinifar SH Sun Y-Z Wang A Zhou Z. Probiotics as means of diseases control in aquaculture, a review of current knowledge and future perspectives. Front Microbiol. (2018) 9:2429. 10.3389/fmicb.2018.02429 30369918 PMC6194580

[6] Zorriehzahra MJ Delshad ST Adel M Tiwari R Karthik K Dhama K. Probiotics as beneficial microbes in aquaculture: an update on their multiple modes of action: a review. Vet Q. (2016) 36:228–41. 10.1080/01652176.2016.1172132 27075688

[7] Dhama K Mahendran M Tomar S Chauhan R. Beneficial effects of probiotics and prebiotics in livestock and poultry: the current perspectives. Intas Polivet. (2008) 9:1–12.

[8] Cutino-Moguel MT Eades C Rezvani K Armstrong-James D. Immunotherapy for infectious diseases in haematological immunocompromise. Br J Haematol. (2017) 177:348–56. 10.1111/bjh.14595 28369798

[9] Plaza-Diaz J Ruiz-Ojeda FJ Gil-Campos M Gil A. Mechanisms of action of probiotics. Adv Nutr. (2019) 10:S49–66. 10.1093/advances/nmy063 30721959 PMC6363529

[10] Takeuchi O Akira S. Pattern recognition receptors and inflammation. Cell. (2010) 140:805–20. 10.1016/j.cell.2010.01.022 20303872

[11] Bermudez-Brito M Plaza-Díaz J Muñoz-Quezada S Gómez-Llorente C Gil A. Probiotic mechanisms of action. Ann Nutr Metab. (2012) 61:160–74. 10.1159/000342079 23037511

[12] Mazloom K Siddiqi I Covasa M. Probiotics: how effective are they in the fight against obesity? Nutrients. (2019) 11:258. 10.3390/nu11020258 30678355 PMC6412733

[13] Gasbarrini G Bonvicini F Gramenzi A. Probiotics history. J Clin Gastroenterol. (2016) 50:S116–9. 10.1097/MCG.0000000000000697 27741152

[14] Gogineni VK Morrow LE Gregory PJ Malesker MA. Probiotics: history and evolution. J Anc Dis Prev Rem. (2013) 1:1–7. 10.4172/2329-8731.1000107

[15] König H Fröhlich J. Lactic acid bacteria. In: König H Unden G Fröhlich J editors. Biology of Microorganisms on Grapes Must and in Wine. Cham: Springer (2017). p. 3–41. 10.1007/978-3-319-60021-5_1

[16] Mokoena MP. Lactic acid bacteria and their bacteriocins: classification, biosynthesis and applications against uropathogens: a mini-review. Molecules. (2017) 22:1255. 10.3390/molecules22081255 28933759 PMC6152299

[17] Quinto EJ Jiménez P Caro I Tejero J Mateo J Girbés T. Probiotic lactic acid bacteria: a review. Food Nutr Sci. (2014) 5:1765. 10.4236/fns.2014.518190

[18] Vinderola G Ouwehand A Salminen S von Wright A (editors). Lactic Acid Bacteria: Microbiological and Functional Aspects. CRC Press (2019).

[19] Sagheddu V Guidesi E Galletti S Elli M. Original paper selection and characterization criteria of probiotics intended for human use from the past to the future. Food Sci Nutr. (2019) 3. 10.22158/fsns.v3n2p73

[20] Thakur N Rokana N Panwar H. Probiotics: selection criteria, safety and role in health and disease. J Innov Biol. (2016) 3:259–70.

[21] Shokryazdan P Faseleh Jahromi M Liang JB Ho YW. Probiotics: from isolation to application. J Am Coll Nutr. (2017) 36:666–76. 10.1080/07315724.2017.1337529 28937854

[22] Baud D Dimopoulou Agri V Gibson GR Reid G Giannoni E. Using probiotics to flatten the curve of coronavirus disease COVID-2019 pandemic. Front Public Health. (2020) 8:186. 10.3389/fpubh.2020.00186 32574290 PMC7227397

[23] Yao M Xie J Du H McClements DJ Xiao H Li L. Progress in microencapsulation of probiotics: a review. Comprehens Rev Food Sci Food Safety. (2020) 19:857–74. 10.1111/1541-4337.12532 33325164

[24] Iravani S Korbekandi H Mirmohammadi SV. Technology and potential applications of probiotic encapsulation in fermented milk products. J Food Sci Technol. (2015) 52:4679–96. 10.1007/s13197-014-1516-2 26243890 PMC4519473

[25] Hansen LT Allan-Wojtas P Jin Y-L Paulson A. Survival of Ca-alginate microencapsulated *Bifidobacterium* spp. in milk and simulated gastrointestinal conditions. Food Microbiol. (2002) 19:35–45. 10.1006/fmic.2001.0452

[26] Yeung TW Arroyo-Maya IJ McClements DJ Sela DA. Microencapsulation of probiotics in hydrogel particles: enhancing *Lactococcus lactis* subsp. cremoris LM0230 viability using calcium alginate beads. Food Funct. (2016) 7:1797–804. 10.1039/C5FO00801H 26611443

[27] Muhammad Z Ramzan R Zhang S Hu H Hameed A Bakry AM. Comparative assessment of the bioremedial potentials of potato resistant starch-based microencapsulated and non-encapsulated *Lactobacillus plantarum* to alleviate the effects of chronic lead toxicity. Front Microbiol. (2018) 9:1306. 10.3389/fmicb.2018.01306 29971052 PMC6018469

[28] Riaz T Iqbal MW Saeed M Yasmin I Hassanin HA Mahmood S. *In vitro* survival of *Bifidobacterium bifidum* microencapsulated in zein-coated alginate hydrogel microbeads. J Microencapsul. (2019) 36:192–203. 10.1080/02652048.2019.1618403 31076009

[29] Yan F Polk DB. Probiotics and probiotic-derived functional factors-mechanistic insights into applications for intestinal homeostasis. Front Immunol. (2020) 11:1428. 10.3389/fimmu.2020.01428 32719681 PMC7348054

[30] Wang J Zeng Y Wang S Liu H Zhang D Zhang W. Swine-derived probiotic *Lactobacillus plantarum* inhibits growth and adhesion of enterotoxigenic *Escherichia coli* and mediates host defense. Front Microbiol. (2018) 9:1364. 10.3389/fmicb.2018.01364 29997590 PMC6028558

[31] Gaspar C Donders G Palmeira-de-Oliveira R Queiroz J Tomaz C Martinez-de-Oliveira J. Bacteriocin production of the probiotic *Lactobacillus acidophilus* KS400. AMB Express. (2018) 8:153. 10.1186/s13568-018-0679-z 30264211 PMC6160374

[32] Collado MC Gueimonde M Salminen S. Chapter 23 - probiotics in adhesion of pathogens: mechanisms of action. In: Watson RR Preedy VR editors. Bioactive Foods in Promoting Health. Elsevier (2010). p. 353–70. 10.1016/B978-0-12-374938-3.00023-2

[33] Fukuda K. Is it feasible to control pathogen infection by competitive binding of probiotics to the host? Virulence. (2017) 8:1502–5. 10.1080/21505594.2017.1382798 28934003 PMC5810465

[34] Kline KA Fälker S Dahlberg S Normark S Henriques-Normark B. Bacterial adhesins in host-microbe interactions. Cell Host Microbe. (2009) 5:580–92. 10.1016/j.chom.2009.05.011 19527885

[35] Glenting J Beck HC Vrang A Riemann H Ravn P Hansen AM. Anchorless surface associated glycolytic enzymes from *Lactobacillus plantarum* 299v bind to epithelial cells and extracellular matrix proteins. Microbiol Res. (2013) 168:245–53. 10.1016/j.micres.2013.01.003 23395591

[36] Vastano V Salzillo M Siciliano RA Muscariello L Sacco M Marasco R. The E1 beta-subunit of pyruvate dehydrogenase is surface-expressed in *Lactobacillus plantarum* and binds fibronectin. Microbiol Res. (2014) 169:121–7. 10.1016/j.micres.2013.07.013 24054819

[37] Nishiyama K Nakazato A Ueno S Seto Y Kakuda T Takai S. Cell surface-associated aggregation-promoting factor from *Lactobacillus gasseri* SBT 2055 facilitates host colonization and competitive exclusion of *Campylobacter jejuni*. Mol Microbiol1. (2015) 98:712–26. 10.1111/mmi.13153 26239091

[38] Zuo F Appaswamy A Gebreegziabher H Jonsson A-B. Role of sortase A in *Lactobacillus gasseri* Kx110A1 adhesion to gastric epithelial cells and competitive exclusion of *Helicobacter pylori*. Front Microbiol. (2019) 10:2770. 10.3389/fmicb.2019.02770 31849907 PMC6902081

[39] Sharma K Pooranachithra M Balamurugan K Goel G. Probiotic mediated colonization resistance against E. coli infection in experimentally challenged Caenorhabditis elegans. Microb Pathog. (2019) 127:39–47. 10.1016/j.micpath.2018.11.041 30500408

[40] Lim S-Y Loo KW Wong W-L. Synergistic antimicrobial effect of a seaweed-probiotic blend against acute hepatopancreatic necrosis disease (AHPND)-causing *Vibrio parahaemolyticus*. Probiotics Antimicrob Proteins. (2019) 12:906–17. 10.1007/s12602-019-09616-8 31773414

[41] Celebioglu HU Olesen SV Prehn K Lahtinen SJ Brix S Hachem MA. Mucin-and carbohydrate-stimulated adhesion and subproteome changes of the probiotic bacterium *Lactobacillus acidophilus* NCFM. J Proteomics. (2017) 163:102–10. 10.1016/j.jprot.2017.05.015 28533178

[42] Monteagudo-Mera A Rastall RA Gibson GR Charalampopoulos D Chatzifragkou A. Adhesion mechanisms mediated by probiotics and prebiotics and their potential impact on human health. Appl Microbiol Biotechnol. (2019) 103:6463–72. 10.1007/s00253-019-09978-7 31267231 PMC6667406

[43] Falah F Vasiee A Behbahani BA Yazdi FT Moradi S Mortazavi SA. Evaluation of adherence and anti-infective properties of probiotic *Lactobacillus fermentum* strain 4-17 against *Escherichia coli* causing urinary tract infection in humans. Microb Pathog. (2019) 131:246–53. 10.1016/j.micpath.2019.04.006 30974159

[44] García-Ruiz A de Llano DG Esteban-Fernández A Requena T Bartolomé B Moreno-Arribas MV. Assessment of probiotic properties in lactic acid bacteria isolated from wine. Food Microbiol. (2014) 44:220–5. 10.1016/j.fm.2014.06.015 25084666

[45] Pisano MB Viale S Conti S Fadda ME Deplano M Melis MP. Preliminary evaluation of probiotic properties of *Lactobacillus* strains isolated from Sardinian dairy products. Biomed Res Int. (2014) 2014:286390. 10.1155/2014/286390 25054135 PMC4099116

[46] Anderson RC Cookson AL McNabb WC Park Z McCann MJ Kelly WJ. *Lactobacillus plantarum* MB452 enhances the function of the intestinal barrier by increasing the expression levels of genes involved in tight junction formation. BMC Microbiol. (2010) 10:316. 10.1186/1471-2180-10-316 21143932 PMC3004893

[47] Chen C-Y Tsen H-Y Lin C-L Yu B Chen C-S. Oral administration of a combination of select lactic acid bacteria strains to reduce the Salmonella invasion and inflammation of broiler chicks. Poult Sci. (2012) 91:2139–47. 10.3382/ps.2012-02237 22912447

[48] Heeney DD Zhai Z Bendiks Z Barouei J Martinic A Slupsky C. *Lactobacillus plantarum* bacteriocin is associated with intestinal and systemic improvements in diet-induced obese mice and maintains epithelial barrier integrity *in vitro*. Gut Microbes. (2019) 10:382–97. 10.1080/19490976.2018.1534513 30409105 PMC6546331

[49] Karczewski J Troost FJ Konings I Dekker J Kleerebezem M Brummer R-JM. Regulation of human epithelial tight junction proteins by *Lactobacillus plantarum in vivo* and protective effects on the epithelial barrier. Am J Physiol Gastrointestinal Liver Physiol. (2010) 298:G851–9. 10.1152/ajpgi.00327.2009 20224007

[50] Qin H Zhang Z Hang X Jiang Y. *L. plantarum* prevents enteroinvasive *Escherichia coli*-induced tight junction proteins changes in intestinal epithelial cells. BMC Microbiol. (2009) 9:63. 10.1186/1471-2180-9-63 19331693 PMC2674056

[51] Zhang Y-g Wu S Xia Y Sun J. *Salmonella* infection upregulates the leaky protein claudin-2 in intestinal epithelial cells. PLoS ONE. (2013) 8:e58606. 10.1371/journal.pone.0058606 23505542 PMC3594366

[52] Stavropoulou E Bezirtzoglou E. Probiotics as a weapon in the fight against COVID-19. Front Nutr. (2020) 7:614986. 10.3389/fnut.2020.614986 33385008 PMC7769760

[53] Lievin-Le Moal V Amsellem R Servin A Coconnier M. *Lactobacillus acidophilus* (strain LB) from the resident adult human gastrointestinal microflora exerts activity against brush border damage promoted by a diarrhoeagenic *Escherichia coli* in human enterocyte-like cells. Gut. (2002) 50:803–11. 10.1136/gut.50.6.803 12010882 PMC1773224

[54] Parassol N Freitas M Thoreux K Dalmasso G Bourdet-Sicard R Rampal P. *Lactobacillus casei* DN-114 001 inhibits the increase in paracellular permeability of enteropathogenic *Escherichia coli*-infected T84 cells. Res Microbiol. (2005) 156:256–62. 10.1016/j.resmic.2004.09.013 15748992

[55] Wang Y Kasper LH. The role of microbiome in central nervous system disorders. Brain Behav Immun. (2014) 38:1–12. 10.1016/j.bbi.2013.12.015 24370461 PMC4062078

[56] Alvarez C-S Giménez R Cañas M-A Vera R Díaz-Garrido N Badia J. Extracellular vesicles and soluble factors secreted by *Escherichia coli* Nissle 1917 and ECOR63 protect against enteropathogenic *E. coli*-induced intestinal epithelial barrier dysfunction. BMC Microbiol. (2019) 19:166. 10.1186/s12866-019-1534-3 31315566 PMC6637528

[57] Bhat MI Kapila S Kapila R. *Lactobacillus fermentum* (MTCC-5898) supplementation renders prophylactic action against *Escherichia coli* impaired intestinal barrier function through tight junction modulation. LWT. (2020) 123:109118. 10.1016/j.lwt.2020.109118

[58] Wang Y Yan X Han D Liu Y Song W Tong T. *Lactobacillus casei* DBN023 protects against jejunal mucosal injury in chicks infected with *Salmonella pullorum* CMCC-533. Res Vet Sci. (2019) 127:33–41. 10.1016/j.rvsc.2019.09.010 31677414

[59] Garg S Singh T Reddi S Malik R Kapila S. Intervention of probiotic *L. reuteri* fermented milk as an adjuvant to combat protein energy malnourishment induced gut disturbances in albino mice. J Funct Foods. (2017) 36:467–79. 10.1016/j.jff.2017.07.017

[60] Yang X Yousef AE. Antimicrobial peptides produced by *Brevibacillus* spp.: structure, classification and bioactivity: a mini review. World J Microbiol Biotechnol. (2018) 34:57. 10.1007/s11274-018-2437-4 29594558

[61] Baindara P Korpole S Grover V. Bacteriocins: perspective for the development of novel anticancer drugs. Appl Microbiol Biotechnol. (2018) 102:10393–408. 10.1007/s00253-018-9420-8 30338356

[62] Mandal SM Pati BR Chakraborty R Franco OL. New insights into the bioactivity of peptides from probiotics. Front Biosci. (2016) 8:450–9. 10.2741/e779 27100351

[63] Messaoudi S Manai M Kergourlay G Prévost H Connil N Chobert J-M. *Lactobacillus salivarius*: bacteriocin and probiotic activity. Food Microbiol. (2013) 36:296–304. 10.1016/j.fm.2013.05.010 24010610

[64] Borrero J Kelly E O'Connor PM Kelleher P Scully C Cotter PD. Plantaricyclin A, a novel circular bacteriocin produced by *Lactobacillus plantarum* NI326: purification, characterization, and heterologous production. Appl Environ Microbiol. (2018) 84:e01801–17. 10.1128/AEM.01801-17 29030449 PMC5734033

[65] Oleskin AV Shenderov BA. Probiotics and psychobiotics: the role of microbial neurochemicals. Probiotics Antimicrob Proteins. (2019) 11:1071–85. 10.1007/s12602-019-09583-0 31493127

[66] Dinev T Beev G Tzanova M Denev S Dermendzhieva D Stoyanova A. Antimicrobial activity of *Lactobacillus plantarum* against pathogenic and food spoilage microorganisms: a review. Bulgarian J Vet Med. (2018) 21:253–68. 10.15547/bjvm.1084

[67] Sahoo TK Jena PK Patel AK Seshadri S. Bacteriocins and their applications for the treatment of bacterial diseases in aquaculture: a review. Aquac Res. (2016) 47:1013–27. 10.1111/are.12556

[68] Gómez-Llorente C Munoz S Gil A. Role of Toll-like receptors in the development of immunotolerance mediated by probiotics. Proc Nutr Soc. (2010) 69:381–9. 10.1017/S0029665110001527 20416121

[69] Lebeer S Vanderleyden J De Keersmaecker SC. Host interactions of probiotic bacterial surface molecules: comparison with commensals and pathogens. Nat Rev Microbiol. (2010) 8:171–84. 10.1038/nrmicro2297 20157338

[70] Rhayat L Maresca M Nicoletti C Perrier J Brinch KS Christian S. Effect of *Bacillus subtilis* strains on intestinal barrier function and inflammatory response. Front Immunol. (2019) 10:564. 10.3389/fimmu.2019.00564 30984172 PMC6449611

[71] Foligné B Dewulf J Breton J Claisse O Lonvaud-Funel A Pot B. Probiotic properties of non-conventional lactic acid bacteria: immunomodulation by *Oenococcus oeni*. Int J Food Microbiol. (2010) 140:136–45. 10.1016/j.ijfoodmicro.2010.04.007 20452078

[72] Savan R Sakai M. Genomics of fish cytokines. Compar Biochem Physiol Part D Genomics Proteomics. (2006) 1:89–101. 10.1016/j.cbd.2005.08.005 20483237

[73] Azad M Kalam A Sarker M Wan D. Immunomodulatory effects of probiotics on cytokine profiles. Biomed Res Int. (2018) 2018:8063647. 10.1155/2018/8063647 30426014 PMC6218795

[74] Chiba Y Shida K Nagata S Wada M Bian L Wang C. Well-controlled proinflammatory cytokine responses of Peyer's patch cells to probiotic *Lactobacillus casei*. Immunology. (2010) 130:352–62. 10.1111/j.1365-2567.2009.03204.x 20636824 PMC2913215

[75] Haller D Bode C Hammes W Pfeifer A Schiffrin E Blum S. Non-pathogenic bacteria elicit a differential cytokine response by intestinal epithelial cell/leucocyte co-cultures. Gut. (2000) 47:79–87. 10.1136/gut.47.1.79 10861268 PMC1727962

[76] Peña JA Rogers AB Ge Z Ng V Li SY Fox JG. Probiotic *Lactobacillus* spp. diminish *Helicobacter hepaticus*-induced inflammatory bowel disease in interleukin-10-deficient mice. Infect Immun. (2005) 73:912–20. 10.1128/IAI.73.2.912-920.2005 15664933 PMC547020

[77] Kourelis A Zinonos I Kakagianni M Christidou A Christoglou N Yiannaki E. Validation of the dorsal air pouch model to predict and examine immunostimulatory responses in the gut. J Appl Microbiol. (2010) 108:274–84. 10.1111/j.1365-2672.2009.04421.x 20002910

[78] Wells JM. Immunomodulatory mechanisms of lactobacilli. Paper presented at the Microbial Cell Factories. Wageningen: University of Wageningen, Animal Sciences (2011). 10.1186/1475-2859-10-S1-S17 PMC3231924 21995674

[79] Kwon H-K Lee C-G So J-S Chae C-S Hwang J-S Sahoo A. Generation of regulatory dendritic cells and CD4+ Foxp3+ T cells by probiotics administration suppresses immune disorders. Proc Natl Acad Sci USA. (2010) 107:2159–64. 10.1073/pnas.0904055107 20080669 PMC2836639

[80] Pinto MGV Gómez MR Seifert S Watzl B Holzapfel WH Franz CM. Lactobacilli stimulate the innate immune response and modulate the TLR expression of HT29 intestinal epithelial cells *in vitro*. Int J Food Microbiol. (2009) 133:86–93. 10.1016/j.ijfoodmicro.2009.05.013 19523707

[81] Abreu MT Fukata M Arditi M. TLR signaling in the gut in health and disease. J Immunol. (2005) 174:4453–60. 10.4049/jimmunol.174.8.4453 15814663

[82] Castillo NA Perdigón G de LeBlanc AdM. Oral administration of a probiotic Lactobacillus modulates cytokine production and TLR expression improving the immune response against *Salmonella enterica* serovar Typhimurium infection in mice. BMC Microbiol. (2011) 11:177. 10.1186/1471-2180-11-177 21813005 PMC3173335

[83] Weiss DS Raupach B Takeda K Akira S Zychlinsky A. Toll-like receptors are temporally involved in host defense. J Immunol. (2004) 172:4463–9. 10.4049/jimmunol.172.7.4463 15034062

[84] Giahi L Aumueller E Elmadfa I Haslberger A. Regulation of TLR4, p38 MAPkinase, IκB and miRNAs by inactivated strains of lactobacilli in human dendritic cells. Benef Microbes. (2012) 3:91–8. 10.3920/BM2011.0052 22476320

[85] Lee J Mo J-H Katakura K Alkalay I Rucker AN Liu Y-T. Maintenance of colonic homeostasis by distinctive apical TLR9 signalling in intestinal epithelial cells. Nat Cell Biol. (2006) 8:1327–36. 10.1038/ncb1500 17128265

[86] Ghadimi D Vrese Md Heller KJ Schrezenmeir J. Effect of natural commensal-origin DNA on toll-like receptor 9 (TLR9) signaling cascade, chemokine IL-8 expression, and barrier integritiy of polarized intestinal epithelial cells. Inflamm Bowel Dis. (2010) 16:410–27. 10.1002/ibd.21057 19714766

[87] Verstrepen L Bekaert T Chau T-L Tavernier J Chariot A Beyaert R. TLR-4, IL-1R and TNF-R signaling to NF-κB: variations on a common theme. Cell Mol Life Sci. (2008) 65:2964–78. 10.1007/s00018-008-8064-8 18535784 PMC11131687

[88] Hee Kim C Geun Kim H Yun Kim J Ra Kim N Jun Jung B Hye Jeong J. Probiotic genomic DNA reduces the production of pro-inflammatory cytokine tumor necrosis factor-alpha. FEMS Microbiol Lett. (2012) 328:13–9. 10.1111/j.1574-6968.2011.02470.x 22126103

[89] Hakansson A Molin G. Gut microbiota and inflammation. Nutrients. (2011) 3:637–82. 10.3390/nu3060637 22254115 PMC3257638

[90] Biswas A Petnicki-Ocwieja T Kobayashi KS. Nod2: a key regulator linking microbiota to intestinal mucosal immunity. J Mol Med. (2012) 90:15–24. 10.1007/s00109-011-0802-y 21861185 PMC3263373

[91] Chen G Shaw MH Kim Y-G Nuñez G. NOD-like receptors: role in innate immunity and inflammatory disease. Annu Rev Pathol Mech Dis. (2009) 4:365–98. 10.1146/annurev.pathol.4.110807.092239 18928408

[92] Fernandez EM Valenti V Rockel C Hermann C Pot B Boneca IG. Anti-inflammatory capacity of selected lactobacilli in experimental colitis is driven by NOD2-mediated recognition of a specific peptidoglycan-derived muropeptide. Gut. (2011) 60:1050–9. 10.1136/gut.2010.232918 21471573

[93] Tohno M Shimosato T Aso H Kitazawa H. Immunobiotic *Lactobacillus* strains augment NLRP3 expression in newborn and adult porcine gut-associated lymphoid tissues. Vet Immunol Immunopathol. (2011) 144:410–6. 10.1016/j.vetimm.2011.09.010 22024502

[94] Bhardwaj R Singh BP Sandhu N Singh N Kaur R Rokana N. Probiotic mediated NF-κB regulation for prospective management of type 2 diabetes. Mol Biol Rep. (2020) 47:2301–13. 10.1007/s11033-020-05254-4 31919753

[95] Tang C Zhu G. Classic and novel signaling pathways involved in cancer: targeting the NF-κB and Syk signaling pathways. Curr Stem Cell Res Ther. (2019) 14:219–25. 10.2174/1574888X13666180723104340 30033874

[96] Tien M-T Girardin SE Regnault B Le Bourhis L Dillies M-A Coppée J-Y. Anti-inflammatory effect of *Lactobacillus casei* on Shigella-infected human intestinal epithelial cells. J Immunol. (2006) 176:1228–37. 10.4049/jimmunol.176.2.1228 16394013

[97] Johnson-Henry KC Nadjafi M Avitzur Y Mitchell DJ Ngan B-Y Galindo-Mata E. Amelioration of the effects of *Citrobacter rodentium* infection in mice by pretreatment with probiotics. J Infect Dis. (2005) 191:2106–17. 10.1086/430318 15897997

[98] Kim SW Kim HM Yang KM Kim S-A Kim S-K An MJ. Bifidobacterium lactis inhibits NF-κB in intestinal epithelial cells and prevents acute colitis and colitis-associated colon cancer in mice. Inflamm Bowel Dis. (2010) 16:1514–25. 10.1002/ibd.21262 20310012

[99] Bu X Lian X Wang Y Luo C Tao S Liao Y. Dietary yeast culture modulates immune response related to TLR2-MyD88-NF-kβ signaling pathway, antioxidant capability and disease resistance against Aeromonas hydrophila for Ussuri catfish (*Pseudobagrus ussuriensis*). Fish Shellfish Immunol. (2019) 84:711–8. 10.1016/j.fsi.2018.10.049 30359752

[100] Iyer C Kosters A Sethi G Kunnumakkara AB Aggarwal BB Versalovic J. Probiotic *Lactobacillus reuteri* promotes TNF-induced apoptosis in human myeloid leukemia-derived cells by modulation of NF-κB and MAPK signalling. Cell Microbiol. (2008) 10:1442–52. 10.1111/j.1462-5822.2008.01137.x 18331465

[101] Kaci G Lakhdari O Doré J Ehrlich SD Renault P Blottière HM. Inhibition of the NF-κB pathway in human intestinal epithelial cells by commensal Streptococcus salivarius. Appl Environ Microbiol. (2011) 77:4681–4. 10.1128/AEM.03021-10 21602373 PMC3127691

[102] Liu Y Fatheree NY Mangalat N Rhoads JM. *Lactobacillus reuteri* strains reduce incidence and severity of experimental necrotizing enterocolitis via modulation of TLR4 and NF-κB signaling in the intestine. Am J Physiol Gastrointestinal Liver Physiol. (2012) 302:G608–17. 10.1152/ajpgi.00266.2011 22207578 PMC3311308

[103] Lee J-M Hwang K-T Jun W-J Park C-S Lee M-Y. Antiinflammatory effect of lactic acid bacteria: inhibition of cyclooxygenase-2 by suppressing nuclear factor-kappaB in Raw264. 7 macrophage cells. J Microbiol Biotechnol. (2008) 18:1683–8. 18955820

[104] Sun K-Y Xu D-H Xie C Plummer S Tang J Yang XF. *Lactobacillus paracasei* modulates LPS-induced inflammatory cytokine release by monocyte-macrophages via the up-regulation of negative regulators of NF-kappaB signaling in a TLR2-dependent manner. Cytokine. (2017) 92:1–11. 10.1016/j.cyto.2017.01.003 28088611

[105] Petrof EO Claud EC Sun J Abramova T Guo Y Waypa TS. Bacteria-free solution derived from *Lactobacillus plantarum* inhibits multiple NF-kappaB pathways and inhibits proteasome function. Inflamm Bowel Dis. (2009) 15:1537–47. 10.1002/ibd.20930 19373789 PMC2748164

[106] Jang SE Hyam S Han M Kim SY Lee BG Kim DH. *Lactobacillus brevis* G-101 ameliorates colitis in mice by inhibiting NF-κB, MAPK and AKT pathways and by polarizing M1 macrophages to M2-like macrophages. J Appl Microbiol. (2013) 115:888–96. 10.1111/jam.12273 23742179

[107] Kemgang TS Kapila S Shanmugam VP Reddi S Kapila R. Fermented milk with probiotic *Lactobacillus rhamnosus* S1K3 (MTCC5957) protects mice from salmonella by enhancing immune and nonimmune protection mechanisms at intestinal mucosal level. J Nutr Biochem. (2016) 30:62–73. 10.1016/j.jnutbio.2015.11.018 27012622

[108] Chen Q Tong C Ma S Zhou L Zhao L Zhao X. Involvement of microRNAs in probiotics-induced reduction of the cecal inflammation by *Salmonella* Typhimurium. Front Immunol. (2017) 8:704. 10.3389/fimmu.2017.00704 28659929 PMC5468434

[109] Martins FS Nardi RM Arantes RM Rosa CA Neves MJ Nicoli JR. Screening of yeasts as probiotic based on capacities to colonize the gastrointestinal tract and to protect against enteropathogen challenge in mice. J Gen Appl Microbiol. (2005) 51:83–92. 10.2323/jgam.51.83 15942869

[110] Martins FS Rodrigues ACP Tiago FC Penna FJ Rosa CA Arantes RM. *Saccharomyces cerevisiae* strain 905 reduces the translocation of *Salmonella enterica* serotype Typhimurium and stimulates the immune system in gnotobiotic and conventional mice. J Med Microbiol. (2007) 56:352–9. 10.1099/jmm.0.46525-0 17314366

[111] Martins FS Elian SD Vieira AT Tiago FC Martins AK Silva FC. Oral treatment with *Saccharomyces cerevisiae* strain UFMG 905 modulates immune responses and interferes with signal pathways involved in the activation of inflammation in a murine model of typhoid fever. Int J Med Microbiol. (2011) 301:359–64. 10.1016/j.ijmm.2010.11.002 21236729

[112] Martins FS Dalmasso G Arantes RM Doye A Lemichez E Lagadec P. Interaction of *Saccharomyces boulardii* with *Salmonella enterica* serovar Typhimurium protects mice and modifies T84 cell response to the infection. PLoS ONE. (2010) 5:e8925. 10.1371/journal.pone.0008925 20111723 PMC2811747

[113] Gebremariam HG Qazi RK Somiah T Psthak SK Sjölinder H Sverremark-Ekström E. *Lactobacillus gasseri* suppresses the production of the proinflammatory cytokines in *Helicobacter pylori*-infected macrophages by inhibiting the expression of ADAM17. Front Immunol. (2019) 10:2326. 10.3389/fimmu.2019.02326 31636639 PMC6788455

[114] Garcia-Castillo V Zelaya H Ilabaca A Espinoza-Monje M Komatsu R Albarracín L. Lactobacillus fermentum UCO-979C beneficially modulates the innate immune response triggered by Helicobacter pylori infection in vitro. Benef Microbes. (2018) 9:829–41. 10.3920/bm2018.0019 29798705

[115] Yu H-J Liu W Chang Z Shen H He L-J Wang S-S. Probiotic BIFICO cocktail ameliorates *Helicobacter pylori* induced gastritis. World J Gastroenterol. (2015) 21:6561. 10.3748/wjg.v21.i21.6561 26074694 PMC4458766

[116] Chen Y-H Tsai W-H Wu H-Y Chen C-Y Yeh W-L Chen Y-H. Probiotic *Lactobacillus* spp. act against Helicobacter pylori-induced inflammation. J Clin Med. (2019) 8:90. 10.3390/jcm8010090 30646625 PMC6352136

[117] Boltin D. Probiotics in *Helicobacter pylori*-induced peptic ulcer disease. Best Prac Res Clin Gastroenterol. (2016) 30:99–109. 10.1016/j.bpg.2015.12.003 27048901

[118] Song H Zhou L Liu D Ge L Li Y. Probiotic effect on Helicobacterápylori attachment and inhibition of inflammation in human gastric epithelial cells. Exp Ther Med. (2019) 18:1551–62. 10.3892/etm.2019.7742 31410109 PMC6676116

[119] Westerik N Reid G Sybesma W Kort R. The probiotic *Lactobacillus rhamnosus* for alleviation of *Helicobacter pylori*-associated gastric pathology in East Africa. Front Microbiol. (2018) 9:1873. 10.3389/fmicb.2018.01873 30154777 PMC6102400

[120] Takeda S Igoshi K Tsend-Ayush C Oyunsuren T Sakata R Koga Y. *Lactobacillus paracasei* strain 06TCa19 suppresses inflammatory chemokine induced by *Helicobacter pylori* in human gastric epithelial cells. Hum Cell. (2017) 30:258–66. 10.1007/s13577-017-0172-z 28434172

[121] Qamar A Aboudola S Warny M Michetti P Pothoulakis C LaMont JT. Microbial immunity and vaccines- *Saccharomyces boulardii* stimulates intestinal immunoglobulin A immune response to *Clostridium difficile* toxin A in mice. Infect Immun. (2001) 69:2762–5. 10.1128/IAI.69.4.2762-2765.2001 11254650 PMC98222

[122] Chen X Kokkotou EG Mustafa N Bhaskar KR Sougioultzis S O'Brien M. *Saccharomyces boulardii* inhibits ERK1/2 mitogen-activated protein kinase activation both *in vitro* and *in vivo* and protects against *Clostridium difficile* toxin A-induced enteritis. J Biol Chem. (2006) 281:24449–54. 10.1074/jbc.M605200200 16816386

[123] Na X Kelly C. Probiotics in *Clostridium difficile* infection. J Clin Gastroenterol. (2011) 45:S154. 10.1097/MCG.0b013e31822ec787 21992956 PMC5322762

[124] Souza RFS Rault L Seyffert N Azevedo V Le Loir Y Even S. *Lactobacillus casei* BL23 modulates the innate immune response in *Staphylococcus aureus*-stimulated bovine mammary epithelial cells. Benef Microbes. (2018) 9:985–95. 10.3920/BM2018.0010 30041534

[125] Paik W Alonzo F Knight KL. Probiotic exopolysaccharide protects against systemic *Staphylococcus aureus* infection, inducing dual-functioning macrophages that restrict bacterial growth and limit inflammation. Infect Immun. (2019) 87:e00791–18. 10.1128/IAI.00791-18 30396894 PMC6300633

[126] Lukic J Jancic I Mirkovic N Bufan B Djokic J Milenkovic M. *Lactococcus lactis* and *Lactobacillus salivarius* differently modulate early immunological response of Wistar rats co-administered with *Listeria monocytogenes*. Benef Microbes. (2017) 8:809–22. 10.3920/BM2017.0007 28856909

[127] Dos Santos LM Santos MM de Souza Silva HP Arantes RME Nicoli JR Vieira LQ. Monoassociation with probiotic *Lactobacillus delbrueckii* UFV-H2b20 stimulates the immune system and protects germfree mice against *Listeria monocytogenes* infection. Med Microbiol Immunol. (2011) 200:29–38. 10.1007/s00430-010-0170-1 20838807

[128] Rachakonda G Vu T Jin L Samanta D Datta PK. Role of TGF-β-induced Claudin-4 expression through c-Jun signaling in non-small cell lung cancer. Cell Signal. (2016) 28:1537–44. 10.1016/j.cellsig.2016.07.006 27424491

[129] Choi HJ Shin MS Lee SM Lee WK. Immunomodulatory properties of *Enterococcus faecium* JWS 833 isolated from duck intestinal tract and suppression of *Listeria monocytogenes* infection. Microbiol Immunol. (2012) 56:613–20. 10.1111/j.1348-0421.2012.00486.x 22709265

[130] Li X-Q Zhu Y-H Zhang H-F Yue Y Cai Z-X Lu Q-P. Risks associated with high-dose *Lactobacillus rhamnosus* in an *Escherichia coli* model of piglet diarrhoea: intestinal microbiota and immune imbalances. PLoS ONE. (2012) 7:e40666. 10.1371/journal.pone.0040666 22848393 PMC3407149

[131] Wang S Peng Q Jia H Zeng X Zhu J Hou C. Prevention of *Escherichia coli* infection in broiler chickens with *Lactobacillus plantarum* B1. Poult Sci. (2017) 96:2576–86. 10.3382/ps/pex061 28482103

[132] Zhang F Zeng X Yang F Huang Z Liu H Ma X. Dietary N-carbamylglutamate supplementation boosts intestinal mucosal immunity in *Escherichia coli* challenged piglets. PLoS ONE. (2013) 8:e66280. 10.1371/journal.pone.0066280 23840434 PMC3686801

[133] Shimazu T Villena J Tohno M Fujie H Hosoya S Shimosato T. Immunobiotic *Lactobacillus jensenii* elicits anti-inflammatory activity in porcine intestinal epithelial cells by modulating negative regulators of the Toll-like receptor signaling pathway. Infect Immun. (2012) 80:276–88. 10.1128/IAI.05729-11 22083706 PMC3255675

[134] Finamore A Roselli M Imbinto A Seeboth J Oswald IP Mengheri E. *Lactobacillus amylovorus* inhibits the TLR4 inflammatory signaling triggered by enterotoxigenic *Escherichia coli* via modulation of the negative regulators and involvement of TLR2 in intestinal Caco-2 cells and pig explants. PLoS ONE. (2014) 9:e94891. 10.1371/journal.pone.0094891 24733511 PMC3986366

[135] Wachi S Kanmani P Tomosada Y Kobayashi H Yuri T Egusa S. *Lactobacillus delbrueckii* TUA 4408 L and its extracellular polysaccharides attenuate enterotoxigenic *Escherichia coli*-induced inflammatory response in porcine intestinal epitheliocytes via T oll-like receptor-2 and 4. Mol Nutr Food Res. (2014) 58:2080–93. 10.1002/mnfr.201400218 24995380

[136] Zhang W Zhu Y-H Yang J-C Yang G-Y Zhou D Wang J-F. A selected *Lactobacillus rhamnosus* strain promotes EGFR-independent Akt activation in an enterotoxigenic *Escherichia coli* K88-infected IPEC-J2 cell model. PLoS ONE. (2015) 10:e125717. 10.1371/journal.pone.0125717 25915861 PMC4411159

[137] Zhen W Shao Y Gong X Wu Y Geng Y Wang Z. Effect of dietary *Bacillus coagulans* supplementation on growth performance and immune responses of broiler chickens challenged by *Salmonella enteritidis*. Poult Sci. (2018) 97:2654–66. 10.3382/ps/pey119 29660095

[138] Gallati C Stephan R Hächler H Malorny B Schroeter A Nüesch-Inderbinen M. Characterization of *Salmonella enterica* subsp. enterica serovar 4,[5], 12: i:-clones isolated from human and other sources in Switzerland between 2007 and 2011. Foodborne Pathog Dis. (2013) 10:549–54. 10.1089/fpd.2012.1407 23614800

[139] Gilchrist JJ MacLennan CA Hill AV. Genetic susceptibility to invasive Salmonella disease. Nat Rev Immunol. (2015) 15:452–63. 10.1038/nri3858 26109132

[140] Majowicz S Musto J Scallan E Angulo F Kir KO Brien SJ. The global burden of collibacillosis. Clin Infect Dis. (2010) 50:882–9. 10.1086/650733 20158401

[141] Mascaro V Pileggi C Crinò M Proroga YTR Carullo MR Graziani C. Non-typhoidal Salmonella in Calabria, Italy: a laboratory and patient-based survey. BMJ Open. (2017) 7:e017037. 10.1136/bmjopen-2017-017037 28893751 PMC5595191

[142] Zhang W Wu Q Zhu Y-H Yang G Yu J Wang J. Probiotic *Lactobacillus rhamnosus* GG induces alterations in ileal microbiota with associated CD3– CD19– T-bet+ IFNγ+/– cell subset homeostasis in pigs challenged with Salmonella enterica serovar 4,[5], 12: i. Front Microbiol. (2019) 10:977. 10.3389/fmicb.2019.00977 31134022 PMC6516042

[143] Gut AM Vasiljevic T Yeager T Donkor O. *Salmonella* infection-prevention and treatment by antibiotics and probiotic yeasts: a review. Microbiology. (2018) 164:1327–44. 10.1099/mic.0.000709 30136920

[144] Ruby T McLaughlin L Gopinath S Monack D. Salmonella's long-term relationship with its host. FEMS Microbiol Rev. (2012) 36:600–15. 10.1111/j.1574-6976.2012.00332.x 22335190

[145] Ertelt JM Johanns TM Mysz MA Nanton MR Rowe JH Aguilera MN. Selective culling of high avidity antigen-specific CD4+ T cells after virulent *Salmonella* infection. Immunology. (2011) 134:487–97. 10.1111/j.1365-2567.2011.03510.x 22044420 PMC3230801

[146] Pontier-Bres R Munro P Boyer L Anty R Imbert V Terciolo C. *Saccharomyces boulardii* modifies *Salmonella* typhimurium traffic and host immune responses along the intestinal tract. PLoS ONE. (2014) 9:e103069. 10.1371/journal.pone.0103069 25118595 PMC4145484

[147] Dolowschiak T Mueller AA Pisan LJ Feigelman R Felmy B Sellin ME. IFN-γ hinders recovery from mucosal inflammation during antibiotic therapy for Salmonella gut infection. Cell Host Microbe. (2016) 20:238–49. 10.1016/j.chom.2016.06.008 27453483

[148] Martins FS Vieira AT Elian SD Arantes RM Tiago FC Sousa LP. Inhibition of tissue inflammation and bacterial translocation as one of the protective mechanisms of *Saccharomyces boulardii* against *Salmonella* infection in mice. Microbes Infect. (2013) 15:270–9. 10.1016/j.micinf.2012.12.007 23376166

[149] Rooks MG Garrett WS. Gut microbiota, metabolites and host immunity. Nat Rev Immunol. (2016) 16:341–52. 10.1038/nri.2016.42 27231050 PMC5541232

[150] Pradhan B Guha D Naik AK Banerjee A Tambat S Chawla S. Probiotics *L. acidophilus* and *B. clausii* modulate gut microbiota in Th1-and Th2-biased mice to ameliorate salmonella typhimurium-induced diarrhea. Probiotics Antimicrob Proteins. (2019) 11:887–904. 10.1007/s12602-018-9436-5 29909486

[151] Rokana N Singh R Mallappa RH Batish VK Grover S. Modulation of intestinal barrier function to ameliorate *Salmonella* infection in mice by oral administration of fermented milks produced with *Lactobacillus plantarum* MTCC 5690–a probiotic strain of Indian gut origin. J Med Microbiol. (2016) 65:1482–93. 10.1099/jmm.0.000366 27902414

[152] Carter A Adams M La Ragione RM Woodward MJ. Colonisation of poultry by *Salmonella Enteritidis* S1400 is reduced by combined administration of *Lactobacillus salivarius* 59 and *Enterococcus faecium* PXN-33. Vet Microbiol. (2017) 199:100–7. 10.1016/j.vetmic.2016.12.029 28110775

[153] de Jesus Souza M de Moraes JA Da Silva VN Helal-Neto E Uberti AF Scopel-Guerra A. *Helicobacter pylori* urease induces pro-inflammatory effects and differentiation of human endothelial cells: cellular and molecular mechanism. Helicobacter. (2019) 24:e12573. 10.1111/hel.12573 30907046

[154] De Vries AC Van Driel HF Richardus JH Ouwendijk M Van Vuuren AJ De Man RA. Migrant communities constitute a possible target population for primary prevention of *Helicobacter pylori*-related complications in low incidence countries. Scand J Gastroenterol. (2008) 43:403–9. 10.1080/00365520701814077 18365904

[155] Mori N Wada A Hirayama T Parks TP Stratowa C Yamamoto N. Activation of intercellular adhesion molecule 1 expression by *Helicobacter pylori* is regulated by NF-κB in gastric epithelial cancer cells. Infect Immun. (2000) 68:1806–14. 10.1128/IAI.68.4.1806-1814.2000 10722567 PMC97351

[156] Kim S-H Woo H Park M Rhee K-J Moon C Lee D. Cyanidin 3-O-glucoside reduces *Helicobacter pylori* VacA-induced cell death of gastric KATO III cells through inhibition of the SecA pathway. Int J Med Sci. (2014) 11:742. 10.7150/ijms.7167 24904230 PMC4045794

[157] Tegtmeyer N Neddermann M Asche CI Backert S. Subversion of host kinases: a key network in cellular signaling hijacked by *Helicobacter pylori* CagA. Mol Microbiol. (2017) 105:358–72. 10.1111/mmi.13707 28508421

[158] Kim SO Sheikh HI Ha SD Martins A Reid G. G-CSF-mediated inhibition of JNK is a key mechanism for *Lactobacillus rhamnosus*-induced suppression of TNF production in macrophages. Cell Microbiol. (2006) 8:1958–71. 10.1111/j.1462-5822.2006.00763.x 16889627

[159] Myllyluoma E Ahonen A-M Korpela R Vapaatalo H Kankuri E. Effects of multispecies probiotic combination on *Helicobacter pylori* infection *in vitro*. Clin Vaccine Immunol. (2008) 15:1472–82. 10.1128/CVI.00080-08 18579692 PMC2546684

[160] Myllyluoma E Kajander K Mikkola H Kyrönpalo S Rasmussen M Kankuri E. Probiotic intervention decreases serum gastrin-17 in *Helicobacter pylori* infection. Digestive Liver Dis. (2007) 39:516–23. 10.1016/j.dld.2007.02.015 17433799

[161] Rokka S Myllykangas S Joutsjoki V. Effect of specific colostral antibodies and selected lactobacilli on the adhesion of *Helicobacter pylori* on AGS cells and the Helicobacter-induced IL-8 production. Scand J Immunol. (2008) 68:280–6. 10.1111/j.1365-3083.2008.02138.x 18627549

[162] Kim J-E Kim M-S Yoon Y-S Chung M-J Yum D-Y. Use of selected lactic acid bacteria in the eradication of *Helicobacter pylori* infection. J Microbiol. (2014) 52:955–62. 10.1007/s12275-014-4355-y 25277407

[163] Tamura A Kumai H Nakamichi N Sugiyama T Deguchi R Takagi A. Suppression of *Helicobacter pylori*-induced interleukin-8 production *in vitro* and within the gastric mucosa by a live *Lactobacillus* strain. J Gastroenterol Hepatol. (2006) 21:1399–406. 10.1111/j.1440-1746.2006.04318.x 16911683

[164] Fleckenstein JM Hardwidge PR Munson GP Rasko DA Sommerfelt H Steinsland H. Molecular mechanisms of enterotoxigenic *Escherichia coli* infection. Microbes Infect. (2010) 12:89–98. 10.1016/j.micinf.2009.10.002 19883790 PMC10647112

[165] Fairbrother JM Nadeau É Gyles CL. *Escherichia coli* in postweaning diarrhea in pigs: an update on bacterial types, pathogenesis, and prevention strategies. Anim Health Res Rev. (2005) 6:17. 10.1079/AHR2005105 16164007

[166] Amdekar S Singh V Singh DD. Probiotic therapy: immunomodulating approach toward urinary tract infection. Curr Microbiol. (2011) 63:484. 10.1007/s00284-011-0006-2 21901556

[167] Sharma R Kapila R Dass G Kapila S. Improvement in Th1/Th2 immune homeostasis, antioxidative status and resistance to pathogenic *E. coli* on consumption of probiotic *Lactobacillus rhamnosus* fermented milk in aging mice. Age. (2014) 36:1–17. 10.1007/s11357-014-9686-4 25037247 PMC4150900

[168] Sharma R Kapila R Kapasiya M Saliganti V Dass G Kapila S. Dietary supplementation of milk fermented with probiotic *Lactobacillus fermentum* enhances systemic immune response and antioxidant capacity in aging mice. Nutr Res. (2014) 34:968–81. 10.1016/j.nutres.2014.09.006 25311611

[169] Bhat MI Sowmya K Kapila S Kapila R. *Escherichia coli* K12: an evolving opportunistic commensal gut microbe distorts barrier integrity in human intestinal cells. Microb Pathog. (2019) 133:103545. 10.1016/j.micpath.2019.103545 31112772

[170] Bhat MI Sowmya K Kapila S Kapila R. Potential probiotic *Lactobacillus rhamnosus* (MTCC-5897) inhibits *Escherichia coli* impaired intestinal barrier function by modulating the host tight junction gene response. Probiotics Antimicrob Proteins. (2019) 12:1149–60. 10.1007/s12602-019-09608-8 31732863

[171] Ma GK Brensinger CM Wu Q Lewis JD. Increasing incidence of multiply recurrent *Clostridium difficile* infection in the United States: a cohort study. Ann Intern Med. (2017) 167:152–8. 10.7326/M16-2733 28672282

[172] Sharma SK Nakajima K Shukla PJ. *Clostridium difficile* infection. N Engl J Med. (2015) 373:287. 10.1056/NEJMc1506004 26176399

[173] Hell M Bernhofer C Stalzer P Kern J Claassen E. Probiotics in *Clostridium difficile* infection: reviewing the need for a multistrain probiotic. Benef Microbes. (2013) 4:39–51. 10.3920/BM2012.0049 23434948

[174] Ollech JE Shen NT Crawford CV Ringel Y. Use of probiotics in prevention and treatment of patients with *Clostridium difficile* infection. Best Prac Res Clin Gastroenterol. (2016) 30:111–8. 10.1016/j.bpg.2016.01.002 27048902

[175] Wu J Ding Y Wang J Wang F. *Staphylococcus aureus* induces TGF-β1 and bFGF expression through the activation of AP-1 and NF-κB transcription factors in bovine mammary epithelial cells. Microb Pathog. (2018) 117:276–84. 10.1016/j.micpath.2018.02.024 29452196

[176] Paynich ML Jones-Burrage SE Knight KL. Exopolysaccharide from *Bacillus subtilis* induces anti-inflammatory M2 macrophages that prevent T cell–mediated disease. J Immunol. (2017) 198:2689–98. 10.4049/jimmunol.1601641 28202619 PMC5360499

[177] Morgand M Leclercq A Maury M Bracq-Dieye H Thouvenot P Vales G. *Listeria monocytogenes*-associated respiratory infections: a study of 38 consecutive cases. Clin Microbiol Infect. (2018) 24:1339.e1–5. 10.1016/j.cmi.2018.03.003 29549058

[178] Jordan K McAuliffe O. *Listeria monocytogenes* in foods. Adv Food Nutr Res. (2018) 86:181–213. 10.1016/bs.afnr.2018.02.006 30077222

[179] Ghosh P Higgins DE. *Listeria monocytogenes* infection of the brain. J Visual Exp. (2018) 140:e58723. 10.3791/58723 PMC6235405 30346400

[180] Djokic J Popovic N Brdaric E Dinic M Terzić-Vidojević A Golic N. The influence of heat-killed *Enterococcus faecium* BGPAS1-3 on the tight junction protein expression and immune function in differentiated Caco-2 cells infected with *Listeria monocytogenes* ATCC 19111. Front Microbiol. (2019) 10:412. 10.3389/fmicb.2019.00412 30891021 PMC6411766

[181] Villena J Shimosato T Vizoso-Pinto MG Kitazawa H. Nutrition, immunity and viral infections. Front Nutr. (2020) 7:125. 10.3389/fnut.2020.00125 33015114 PMC7507487

[182] Saitoh T Yamamoto M Miyagishi M Taira K Nakanishi M Fujita T. A20 is a negative regulator of IFN regulatory factor 3 signaling. J Immunol. (2005) 174:1507–12. 10.4049/jimmunol.174.3.1507 15661910

[183] Ishizuka T Kanmani P Kobayashi H Miyazaki A Soma J Suda Y. Immunobiotic bifidobacteria strains modulate rotavirus immune response in porcine intestinal epitheliocytes via pattern recognition receptor signaling. PLoS ONE. (2016) 11:e152416. 10.1371/journal.pone.0152416 27023883 PMC4811565

[184] Chattha KS Vlasova AN Kandasamy S Rajashekara G Saif LJ. Divergent immunomodulating effects of probiotics on T cell responses to oral attenuated human rotavirus vaccine and virulent human rotavirus infection in a neonatal gnotobiotic piglet disease model. J Immunol. (2013) 191:2446–56. 10.4049/jimmunol.1300678 23918983 PMC4136549

[185] Gonzalez-Ochoa G Flores-Mendoza LK Icedo-Garcia R Gomez-Flores R Tamez-Guerra P. Modulation of rotavirus severe gastroenteritis by the combination of probiotics and prebiotics. Arch Microbiol. (2017) 199:953–61. 10.1007/s00203-017-1400-3 28634691 PMC5548957

[186] Olaya Galán N Ulloa Rubiano J Velez Reyes F Fernandez Duarte K Salas Cardenas S Gutierrez Fernandez M. *In vitro* antiviral activity of *Lactobacillus casei* and *Bifidobacterium adolescentis* against rotavirus infection monitored by NSP 4 protein production. J Appl Microbiol. (2016) 120:1041–51. 10.1111/jam.13069 26801008

[187] Zhang W Azevedo MS Gonzalez AM Saif LJ Van Nguyen T Wen K. Influence of probiotic Lactobacilli colonization on neonatal B cell responses in a gnotobiotic pig model of human rotavirus infection and disease. Vet Immunol Immunopathol. (2008) 122:175–81. 10.1016/j.vetimm.2007.10.003 18023882 PMC2268606

[188] Yamamoto Y Saruta J Takahashi T To M Shimizu T Hayashi T. Effect of ingesting yogurt fermented with *Lactobacillus delbrueckii* ssp. bulgaricus OLL1073R-1 on influenza virus-bound salivary IgA in elderly residents of nursing homes: a randomized controlled trial. Acta Odontol Scand. (2019) 77:517–24. 10.1080/00016357.2019.1609697 31094267

[189] Belkacem N Serafini N Wheeler R Derrien M Boucinha L Couesnon A. *Lactobacillus paracasei* feeding improves immune control of influenza infection in mice. PLoS ONE. (2017) 12:e0184976. 10.1371/journal.pone.0184976 28931041 PMC5607164

[190] Jung Y-J Lee Y-T Le Ngo V Cho Y-H Ko E-J Hong S-M. Heat-killed *Lactobacillus casei* confers broad protection against influenza A virus primary infection and develops heterosubtypic immunity against future secondary infection. Sci Rep. (2017) 7:1–12. 10.1038/s41598-017-17487-8 29234060 PMC5727132

[191] Culley FJ. Natural killer cells in infection and inflammation of the lung. Immunology. (2009) 128:151–63. 10.1111/j.1365-2567.2009.03167.x 19740372 PMC2767305

[192] Ritz BW Nogusa S Ackerman EA Gardner EM. Supplementation with active hexose correlated compound increases the innate immune response of young mice to primary influenza infection. J Nutr. (2006) 136:2868–73. 10.1093/jn/136.11.2868 17056815

[193] Kikuchi Y Kunitoh-Asari A Hayakawa K Imai S Kasuya K Abe K. Oral administration of *Lactobacillus plantarum* strain AYA enhances IgA secretion and provides survival protection against influenza virus infection in mice. PLoS ONE. (2014) 9:e86416. 10.1371/journal.pone.0086416 24466081 PMC3899257

[194] Kawahara T Takahashi T Oishi K Tanaka H Masuda M Takahashi S. Consecutive oral administration of *Bifidobacterium longum* MM-2 improves the defense system against influenza virus infection by enhancing natural killer cell activity in a murine model. Microbiol Immunol. (2015) 59:1–12. 10.1111/1348-0421.12210 25400245

[195] Namba K Hatano M Yaeshima T Takase M Suzuki K. Effects of *Bifidobacterium longum* BB536 administration on influenza infection, influenza vaccine antibody titer, and cell-mediated immunity in the elderly. Biosci Biotechnol Biochem. (2010) 74:939–45. 10.1271/bbb.90749 20460726

[196] Iwabuchi N Xiao J-Z Yaeshima T Iwatsuki K. Oral administration of *Bifidobacterium longum* ameliorates influenza virus infection in mice. Biol Pharm Bull. (2011) 34:1352–5. 10.1248/bpb.34.1352 21804232

[197] Maeda N Nakamura R Hirose Y Murosaki S Yamamoto Y Kase T. Oral administration of heat-killed *Lactobacillus plantarum* L-137 enhances protection against influenza virus infection by stimulation of type I interferon production in mice. Int Immunopharmacol. (2009) 9:1122–5. 10.1016/j.intimp.2009.04.015 19410659

[198] Leyer GJ Li S Mubasher ME Reifer C Ouwehand AC. Probiotic effects on cold and influenza-like symptom incidence and duration in children. Pediatrics. (2009) 124:e172–9. 10.1542/peds.2008-2666 19651563

[199] Liu Y Liu Q Jiang Y Yang W Huang H Shi C. Surface-displayed porcine IFN-λ3 in *Lactobacillus plantarum* inhibits porcine enteric coronavirus infection of porcine intestinal epithelial cells. J Microbiol Biotechnol. (2019) 30:515–25. 10.4014/jmb.1909.09041 31838830 PMC9728374

[200] Yang W-T Li Q-Y Ata EB Jiang Y-L Huang H-B Shi C-W. Immune response characterization of mice immunized with *Lactobacillus plantarum* expressing spike antigen of transmissible gastroenteritis virus. Appl Microbiol Biotechnol. (2018) 102:8307–18. 10.1007/s00253-018-9238-4 30056514 PMC7080198

[201] Jiang X Hou X Tang L Jiang Y Ma G Li Y. A phase trial of the oral *Lactobacillus casei* vaccine polarizes Th2 cell immunity against transmissible gastroenteritis coronavirus infection. Appl Microbiol Biotechnol. (2016) 100:7457–69. 10.1007/s00253-016-7424-9 27020282 PMC7080089

[202] Seo BJ Mun MR Kim C-J Lee I Kim H Park Y-H. Putative probiotic *Lactobacillus* spp. from porcine gastrointestinal tract inhibit transmissible gastroenteritis coronavirus and enteric bacterial pathogens. Trop Anim Health Prod. (2010) 42:1855–60. 10.1007/s11250-010-9648-5 20623187 PMC7089342

[203] Sirichokchatchawan W Temeeyasen G Nilubol D Prapasarakul N. Protective effects of cell-free supernatant and live lactic acid bacteria isolated from Thai pigs against a pandemic strain of porcine epidemic diarrhea virus. Probiotics Antimicrob Proteins. (2018) 10:383–90. 10.1007/s12602-017-9281-y 28434154 PMC7091344

[204] Corano Scheri G Fard SN Schietroma I Mastrangelo A Pinacchio C Giustini N. Modulation of tryptophan/serotonin pathway by probiotic supplementation in human immunodeficiency virus–positive patients: preliminary results of a new study approach. Int J Tryptophan Res. (2017) 10:1178646917710668. 10.1177/1178646917710668 28607543 PMC5457170

[205] Hummelen R Changalucha J Butamanya NL Cook A Habbema JDF Reid G. *Lactobacillus rhamnosus* GR-1 and *L. reuteri* RC-14 to prevent or cure bacterial vaginosis among women with HIV. Int J Gynecol Obstetrics. (2010) 111:245–8. 10.1016/j.ijgo.2010.07.008 20801446

[206] Cunningham-Rundles S Ahrné S Johann-Liang R Abuav R Dunn-Navarra A-M Grassey C. Effect of probiotic bacteria on microbial host defense, growth, and immune function in human immunodeficiency virus type-1 infection. Nutrients. (2011) 3:1042–70. 10.3390/nu3121042 22292110 PMC3260491

[207] Brun P Scarpa M Marchiori C Sarasin G Caputi V Porzionato A. *Saccharomyces boulardii* CNCM I-745 supplementation reduces gastrointestinal dysfunction in an animal model of IBS. PLoS ONE. (2017) 12:e0181863. 10.1371/journal.pone.0181863 28732069 PMC5521842

[208] Palma E Recine N Domenici L Giorgini M Pierangeli A Panici PB. Long-term *Lactobacillus rhamnosus* BMX 54 application to restore a balanced vaginal ecosystem: a promising solution against HPV-infection. BMC Infect Dis. (2018) 18:1–7. 10.1186/s12879-017-2938-z 29304768 PMC5756375

[209] Abdolalipour E Mahooti M Salehzadeh A Torabi A Mohebbi SR Gorji A. Evaluation of the antitumor immune responses of probiotic *Bifidobacterium bifidum* in human papillomavirus-induced tumor model. Microb Pathog. (2020) 145:104207. 10.1016/j.micpath.2020.104207 32325236

[210] Ou Y-C Fu H-C Tseng C-W Wu C-H Tsai C-C Lin H. The influence of probiotics on genital high-risk human papilloma virus clearance and quality of cervical smear: a randomized placebo-controlled trial. BMC Women's Health. (2019) 19:1–7. 10.1186/s12905-019-0798-y 31340789 PMC6657108

[211] Khani S Motamedifar M Golmoghaddam H Hosseini HM Hashemizadeh Z. *In vitro* study of the effect of a probiotic bacterium *Lactobacillus rhamnosus* against herpes simplex virus type 1. Brazil J Infect Dis. (2012) 16:129–35. 10.1016/S1413-8670(12)70293-3 22552453 PMC7128665

[212] Oo KM AYELWIN A Kyaw YY Tun WM Fukada K Goshima A. Safety and long-term effect of the probiotic FK-23 in patients with hepatitis C virus infection. Biosci Microbiota Food Health. (2016) 2015–24. 10.12938/bmfh.2015-024 27508113 PMC4965516

[213] Lopez-Santamarina A Lamas A del Carmen Mondragón A Cardelle-Cobas A Regal P Rodriguez-Avila JA. Probiotic effects against virus infections: new weapons for an old war. Foods. (2021) 10:130. 10.3390/foods10010130 33435315 PMC7827890

[214] Zhang H Yeh C Jin Z Ding L Liu BY Zhang L. Prospective study of probiotic supplementation results in immune stimulation and improvement of upper respiratory infection rate. Synth Syst Biotechnol. (2018) 3:113–20. 10.1016/j.synbio.2018.03.001 29900424 PMC5995450

[215] Chong H-X Yusoff NAA Hor Y-Y Lew L-C Jaafar MH Choi S-B. *Lactobacillus plantarum* DR7 improved upper respiratory tract infections via enhancing immune and inflammatory parameters: a randomized, double-blind, placebo-controlled study. J Dairy Sci. (2019) 102:4783–97. 10.3168/jds.2018-16103 30954261

[216] Preidis GA Saulnier DM Blutt SE Mistretta T-A Riehle KP Major AM. Host response to probiotics determined by nutritional status of rotavirus-infected neonatal mice. J Pediatr Gastroenterol Nutr. (2012) 55:299. 10.1097/MPG.0b013e31824d2548 22343914 PMC4010314

[217] Freedman SB Xie J Nettel-Aguirre A Pang X-L Chui L Williamson-Urquhart S. A randomized trial evaluating virus-specific effects of a combination probiotic in children with acute gastroenteritis. Nat Commun. (2020) 11:1–9. 10.1038/s41467-020-16308-3 32439860 PMC7242434

[218] Pu F Guo Y Li M Zhu H Wang S Shen X. Yogurt supplemented with probiotics can protect the healthy elderly from respiratory infections: a randomized controlled open-label trial. Clin Interv Aging. (2017) 12:1223. 10.2147/CIA.S141518 28848330 PMC5557113

[219] Mitchell PS Patzina C Emerman M Haller O Malik HS Kochs G. Evolution-guided identification of antiviral specificity determinants in the broadly acting interferon-induced innate immunity factor MxA. Cell Host Microbe. (2012) 12:598–604. 10.1016/j.chom.2012.09.005 23084925 PMC3540999

[220] Liang SL Quirk D Zhou A. RNase L: its biological roles and regulation. IUBMB Life. (2006) 58:508–14. 10.1080/15216540600838232 17002978

[221] Morelli M Ogden KM Patton JT. Silencing the alarms: innate immune antagonism by rotavirus NSP1 and VP3. Virology. (2015) 479:75–84. 10.1016/j.virol.2015.01.006 25724417 PMC4940189

[222] Lee DK Park JE Kim MJ Seo JG Lee JH Ha NJ. Probiotic bacteria, *B. longum* and Lacidophilus inhibit infection by rotavirus *in vitro* and decrease the duration of diarrhea in pediatric patients. Clin Res Hepatol Gastroenterol. (2015) 39:237–44. 10.1016/j.clinre.2014.09.006 25459995

[223] Maragkoudakis PA Chingwaru W Gradisnik L Tsakalidou E Cencic A. Lactic acid bacteria efficiently protect human and animal intestinal epithelial and immune cells from enteric virus infection. Int J Food Microbiol. (2010) 141:S91–7. 10.1016/j.ijfoodmicro.2009.12.024 20106541 PMC7114074

[224] Riaz M Alam S Malik A Ali SM. Efficacy and safety of *Saccharomyces boulardii* in acute childhood diarrhea: a double blind randomised controlled trial. Indian J Pediatr. (2012) 79:478–82. 10.1007/s12098-011-0573-z 21997865

[225] Rigo-Adrover M Saldaña-Ruíz S Van Limpt K Knipping K Garssen J Knol J. A combination of scGOS/lcFOS with *Bifidobacterium breve* M-16V protects suckling rats from rotavirus gastroenteritis. Eur J Nutr. (2017) 56:1657–70. 10.1007/s00394-016-1213-1 27112962

[226] Varyukhina S Freitas M Bardin S Robillard E Tavan E Sapin C. Glycan-modifying bacteria-derived soluble factors from Bacteroides thetaiotaomicron and *Lactobacillus casei* inhibit rotavirus infection in human intestinal cells. Microbes Infect. (2012) 14:273–8. 10.1016/j.micinf.2011.10.007 22079149

[227] Kandasamy S Vlasova AN Fischer D Kumar A Chattha KS Rauf A. Differential effects of *Escherichia coli* Nissle and *Lactobacillus rhamnosus* strain GG on human rotavirus binding, infection, and B cell immunity. J Immunol. (2016) 196:1780–9. 10.4049/jimmunol.1501705 26800875 PMC4744595

[228] Ludert JE Feng N Yu JH Broome RL Hoshino Y Greenberg HB. Genetic mapping indicates that VP4 is the rotavirus cell attachment protein *in vitro* and *in vivo*. J Virol. (1996) 70:487–93. 10.1128/JVI.70.1.487-493.1996 8523562 PMC189837

[229] Azevedo M Zhang W Wen K Gonzalez A Saif L Yousef A. *Lactobacillus acidophilus* and *L. reuteri* modulate cytokine responses in gnotobiotic pigs infected with human rotavirus. Benef Microbes. (2012) 3:33. 10.3920/BM2011.0041 22348907 PMC3304463

[230] Lehtoranta L Pitkäranta A Korpela R. Probiotics in respiratory virus infections. Eur J Clin Microbiol Infect Dis. (2014) 33:1289–302. 10.1007/s10096-014-2086-y 24638909 PMC7088122

[231] Takeda S Takeshita M Kikuchi Y Dashnyam B Kawahara S Yoshida H. Efficacy of oral administration of heat-killed probiotics from Mongolian dairy products against influenza infection in mice: alleviation of influenza infection by its immunomodulatory activity through intestinal immunity. Int Immunopharmacol. (2011) 11:1976–83. 10.1016/j.intimp.2011.08.007 21871585

[232] He F Morita H Kubota A Ouwehand AC Hosoda M Hiramatsu M. Effect of orally administered non-viable Lactobacillus cells on murine humoral immune responses. Microbiol Immunol. (2005) 49:993–7. 10.1111/j.1348-0421.2005.tb03695.x 16301810

[233] Inoue R Nishio A Fukushima Y Ushida K. Oral treatment with probiotic *Lactobacillus johnsonii* NCC533 (La1) for a specific part of the weaning period prevents the development of atopic dermatitis induced after maturation in model mice, NC/Nga. Br J Dermatol. (2007) 156:499–509. 10.1111/j.1365-2133.2006.07695.x 17300240

[234] La Gruta NL Kedzierska K Stambas J Doherty PC. A question of self-preservation: immunopathology in influenza virus infection. Immunol Cell Biol. (2007) 85:85–92. 10.1038/sj.icb.7100026 17213831

[235] Yitbarek A Alkie T Taha-Abdelaziz K Astill J Rodriguez-Lecompte J Parkinson J. Gut microbiota modulates type I interferon and antibody-mediated immune responses in chickens infected with influenza virus subtype H9N2. Benef Microbes. (2018) 9:417–27. 10.3920/BM2017.0088 29380643

[236] Barjesteh N Shojadoost B Brisbin JT Emam M Hodgins DC Nagy É. Reduction of avian influenza virus shedding by administration of Toll-like receptor ligands to chickens. Vaccine. (2015) 33:4843–9. 10.1016/j.vaccine.2015.07.070 26238721

[237] Yan N Chen ZJ. Intrinsic antiviral immunity. Nat Immunol. (2012) 13:214. 10.1038/ni.2229 22344284 PMC3549670

[238] Morais AH Passos TS Maciel BL da Silva-Maia JK. Can probiotics and diet promote beneficial immune modulation and purine control in coronavirus infection? Nutrients. (2020) 12:1737. 10.3390/nu12061737 32532069 PMC7352643

[239] Olaimat AN Aolymat I Al-Holy M Ayyash M Ghoush MA Al-Nabulsi AA. The potential application of probiotics and prebiotics for the prevention and treatment of COVID-19. NPJ Sci Food. (2020) 4:1–7. 10.1038/s41538-020-00078-9 33083549 PMC7536434

[240] Sohrabi C Alsafi Z O'Neill N Khan M Kerwan A Al-Jabir A. World Health Organization declares global emergency: a review of the 2019 novel coronavirus (COVID-19). Int J Surg. (2020) 76:71–6. 10.1016/j.ijsu.2020.02.034 32112977 PMC7105032

[241] Zhang G Li B Yoo D Qin T Zhang X Jia Y. Animal coronaviruses and SARS-CoV-2. Transbound Emerg Dis. (2020) 1–14. 10.1111/tbed.13791 32799433 PMC7461065

[242] Chen Y Liu Q Guo D. Emerging coronaviruses: genome structure, replication, and pathogenesis. J Med Virol. (2020) 92:418–23. 10.1002/jmv.25681 31967327 PMC7167049

[243] Gohil K Samson R Dastager S Dharne M. Probiotics in the prophylaxis of COVID-19: something is better than nothing. 3 Biotech. (2021) 11:1–10. 10.1007/s13205-020-02554-1 33262924 PMC7690945

[244] Banerjee A Kulcsar K Misra V Frieman M Mossman K. Bats and coronaviruses. Viruses. (2019) 11:41. 10.3390/v11010041 PMC6356540 30634396

[245] Maxmen A. Slew of trials launch to test coronavirus treatments in China. Nature. (2020) 347–8. 10.1038/d41586-020-00444-3 32071447

[246] Hoffmann M Kleine-Weber H Schroeder S Krüger N Herrler T Erichsen S. SARS-CoV-2 cell entry depends on ACE2 and TMPRSS2 and is blocked by a clinically proven protease inhibitor. Cell. (2020) 181:271–80. e278. 10.1016/j.cell.2020.02.052 32142651 PMC7102627

[247] Zhang H Kang Z Gong H Xu D Wang J Li Z. The digestive system is a potential route of 2019-nCov infection: a bioinformatics analysis based on single-cell transcriptomes. bioRxiv. (2020). 10.1101/2020.01.30.927806

[248] Cascella M Rajnik M Cuomo A Dulebohn SC Di Napoli R. Features, Evaluation and Treatment Coronavirus (COVID-19). Statpearls (2020). 32150360

[249] Pan L Mu M Yang P Sun Y Wang R Yan J. Clinical characteristics of COVID-19 patients with digestive symptoms in Hubei, China: a descriptive, cross-sectional, multicenter study. Am J Gastroenterol. (2020) 115:766–73. 10.14309/ajg.0000000000000620 32287140 PMC7172492

[250] Grech V. Unknown unknowns–COVID-19 and potential global mortality. Early Hum Dev. (2020) 144:105026. 10.1016/j.earlhumdev.2020.105026 32247898 PMC7270771

[251] Infusino F Marazzato M Mancone M Fedele F Mastroianni CM Severino P. Diet supplementation, probiotics, and nutraceuticals in SARS-CoV-2 infection: a scoping review. Nutrients. (2020) 12:1718. 10.3390/nu12061718 32521760 PMC7352781

[252] Li G De Clercq E. Therapeutic Options for the 2019 Novel Coronavirus (2019-nCoV). Nature Publishing Group (2020). 10.1038/d41573-020-00016-0 32127666

[253] Chien M Anderson TK Jockusch S Tao C Li X Kumar S. Nucleotide analogues as inhibitors of SARS-CoV-2 polymerase, a key drug target for COVID-19. J Proteome Res. (2020) 19:4690–7. 10.1021/acs.jproteome.0c00392 32692185 PMC7640960

[254] Ju J Li X Kumar S Jockusch S Chien M Tao C. Nucleotide analogues as inhibitors of SARS-CoV polymerase. Pharmacol Res Perspect. (2020) 8:e00674. 10.1002/prp2.674 33124786 PMC7596664

[255] Kearney JE. Chloroquine as a potential treatment and prevention measure for the 2019 novel coronavirus: a review. Preprints. (2020) 2020030275. 10.20944/preprints202003.0275.v1

[256] Liu C Zhou Q Li Y Garner LV Watkins SP Carter LJ. Research and Development on Therapeutic Agents and Vaccines for COVID-19 and Related Human Coronavirus Diseases. ACS Publications (2020). 10.1021/acscentsci.0c00272 PMC10467574 32226821

[257] Monteil V Kwon H Prado P Hagelkrüys A Wimmer RA Stahl M. Inhibition of SARS-CoV-2 infections in engineered human tissues using clinical-grade soluble human ACE2. Cell. (2020) 181:905–13. e907. 10.1016/j.cell.2020.04.004 32333836 PMC7181998

[258] Popov D. Treatment of Covid-19 infection. a rationale for current and future pharmacological approach. EC Pulmonol Respir Med. (2020) 9.4:38–58.

[259] Zhang L Liu Y. Potential interventions for novel coronavirus in China: a systematic review. J Med Virol. (2020) 92:479–90. 10.1002/jmv.25707 32052466 PMC7166986

[260] Manna S Chowdhury T Chakraborty R Mandal SM. Probiotics-derived peptides and their immunomodulatory molecules can play a preventive role against viral diseases including COVID-19. Probiotics Antimicrob Proteins. (2020) 1–13. 10.1007/s12602-020-09727-7 33226581 PMC7680993

[261] Sundararaman A Ray M Ravindra P Halami PM. Role of probiotics to combat viral infections with emphasis on COVID-19. Appl Microbiol Biotechnol. (2020) 104:8089–104. 10.1007/s00253-020-10832-4 32813065 PMC7434852

[262] Di Pierro F. A possible probiotic (*S. salivarius* K12) approach to improve oral and lung microbiotas and raise defenses against SAR S-CoV-2. Minerva Med. (2020) 111:281–3. 10.23736/S0026-4806.20.06570-2 32255312

[263] Wang X Wang L Huang X Ma S Yu M Shi W. Oral delivery of probiotics expressing dendritic cell-targeting peptide fused with porcine epidemic diarrhea virus COE antigen: a promising vaccine strategy against PEDV. Viruses. (2017) 9:312. 10.3390/v9110312 29068402 PMC5707519

[264] Åkerström S Mousavi-Jazi M Klingström J Leijon M Lundkvist Å Mirazimi A. Nitric oxide inhibits the replication cycle of severe acute respiratory syndrome coronavirus. J Virol. (2005) 79:1966–9. 10.1128/JVI.79.3.1966-1969.2005 15650225 PMC544093

[265] Jung K Gurnani A Renukaradhya GJ Saif LJ. Nitric oxide is elicited and inhibits viral replication in pigs infected with porcine respiratory coronavirus but not porcine reproductive and respiratory syndrome virus. Vet Immunol Immunopathol. (2010) 136:335–9. 10.1016/j.vetimm.2010.03.022 20409593 PMC2902704

[266] Chai W Burwinkel M Wang Z Palissa C Esch B Twardziok S. Antiviral effects of a probiotic *Enterococcus faecium* strain against transmissible gastroenteritis coronavirus. Arch Virol. (2013) 158:799–807. 10.1007/s00705-012-1543-0 23188495 PMC7086644

[267] Liu F Li G Wen K Bui T Cao D Zhang Y. Porcine small intestinal epithelial cell line (IPEC-J2) of rotavirus infection as a new model for the study of innate immune responses to rotaviruses and probiotics. Viral Immunol. (2010) 23:135–49. 10.1089/vim.2009.0088 20373994 PMC2883522

[268] Park Yong HA Seon LI Kim Hong IK. Acid- and Bile-Tolerant Probiotic Enterococcus Faecium Probio-63 Which Can Suppress Growth Of Corona Virus and Porcine Circovirus Type 2 and Pharmaceutical or Food Composition Containing the Same. Republic of Korea KR100468522B (2004).

[269] Park Yong HA Seon LI Kim Hong IK. Acid Tolerant Probiotic Enterococcus Faecalis Probio-056 That Can Suppresses the Growth of Pathogenic Microorganisms and Ped Coronavirus to Stabilize the Intestinal Microflora, and to Treat or Prevent Symptoms Resulted From Abnormal Fermentation of Pathogenic Microorganisms. Republic of Korea KR20050023475 (2003).

[270] Liu Q Jiang Y Yang W Liu Y Shi C Liu J. Protective effects of a food-grade recombinant *Lactobacillus plantarum* with surface displayed AMA1 and EtMIC2 proteins of *Eimeria tenella* in broiler chickens. Microb Cell Fact. (2020) 19:28. 10.1186/s12934-020-1297-4 32046719 PMC7014946

[271] Awais MM Jamal MA Akhtar M Hameed MR Anwar MI Ullah MI. Immunomodulatory and ameliorative effects of Lactobacillus and Saccharomyces based probiotics on pathological effects of eimeriasis in broilers. Microb Pathog. (2019) 126:101–8. 10.1016/j.micpath.2018.10.038 30385394

[272] Pender C Kim S Potter T Ritzi M Young M Dalloul R. Effects of in ovo supplementation of probiotics on performance and immunocompetence of broiler chicks to an Eimeria challenge. Benef Microbes. (2016) 7:699–705. 10.3920/BM2016.0080 27726419

[273] Al-alousi TI Abdullah AH Jasim MM. Prophylactic role of lactic acid prepared from *Lactobacillus acidophilus* on cryptosporidiosis. Tikrit J Pure Sci. (2018) 23:38–42. Available online at: http://dx.doi.oc5130/tjps.23.2018.127

[274] Solano-Aguilar G Shea-Donohue T Madden KB Quinoñes A Beshah E Lakshman S. *Bifidobacterium animalis* subspecies lactis modulates the local immune response and glucose uptake in the small intestine of juvenile pigs infected with the parasitic nematode *Ascaris suum*. Gut Microbes. (2018) 9:422–36. 10.1080/19490976.2018.1460014 30024817 PMC6219643

[275] Jang S Lakshman S Molokin A Urban JF Jr Davis CD Solano-Aguilar G. *Lactobacillus rhamnosus* and flavanol-enriched cocoa powder altered the immune response to infection with the parasitic nematode *Ascaris suum* in a pig model. FASEB J. (2016) 30(1_Suppl):1176.1114.

[276] Thomas DJ Husmann RJ Villamar M Winship TR Buck RH Zuckermann FA. *Lactobacillus rhamnosus* HN001 attenuates allergy development in a pig model. PLoS ONE. (2011) 6:e16577. 10.1371/journal.pone 21386995 PMC3046142

[277] Goyal N Shukla G. Probiotic *Lactobacillus rhamnosus* GG modulates the mucosal immune response in Giardia intestinalis-infected BALB/c mice. Dig Dis Sci. (2013) 58:1218–25. 10.1007/s10620-012-2503-y 23263901

[278] Benyacoub J Perez P Rochat F Saudan K Reuteler G Antille N. *Enterococcus faecium* SF68 enhances the immune response to Giardia intestinalis in mice. J Nutr. (2005) 135:1171–6. 10.1093/jn/135.5.1171 15867299

[279] Ghanem KZ Abdel-Salam AM Magharby AS. Immunoprophylactic effect of probiotic yoghurt feeding on *Schistosoma mansoni*-infected mice. Pol J Food Nutr Sci. (2005) 14–55:123–6.

[280] Zowail ME Osman GY Mohamed AH El-Esawy HMI. Protective role of *Lactobacillus sporogenes* (probiotic) on chromosomal aberrations and DNA fragmentation in *Schistosoma mansoni* infected mice. Egypt J Exp Biol. (2012) 8:121–30.

[281] El Temsahy MM Ibrahim IR Mossallam SF Mahrous H Bary AA Salam SAA. Evaluation of newly isolated probiotics in the protection against experimental intestinal trichinellosis. Vet Parasitol. (2015) 214:303–14. 10.1016/j.vetpar.2015.08.029 26386829

[282] DvoroŽnáková E Bucková B Hurníková Z Revajová V Lauková A. Effect of probiotic bacteria on phagocytosis and respiratory burst activity of blood polymorphonuclear leukocytes (PMNL) in mice infected with *Trichinella spiralis*. Vet Parasitol. (2016) 231:69–76. 10.1016/j.vetpar.2016.07.004 27425573

[283] Martínez-Gómez F Fuentes-Castro BE Bautista-Garfias CR. The intraperitoneal inoculation of *Lactobacillus casei* in mice induces total protection against *Trichinella spiralis* infection at low challenge doses. Parasitol Res. (2011) 109:1609–17. 10.1007/s00436-011-2432-2 21541750

[284] McClemens J Kim JJ Wang H Mao Y-K Collins M Kunze W. *Lactobacillus rhamnosus* ingestion promotes innate host defense in an enteric parasitic infection. Clin Vaccine Immunol. (2013) 20:818–26. 10.1128/CVI.00047-13 23536695 PMC3675974

[285] de Avila LFDC De Leon PMM De Moura MQ Berne MEA Scaini CJ Leivas Leite FP. Modulation of IL-12 and IFN γ by probiotic supplementation promotes protection against *Toxocara canis* infection in mice. Parasite Immunol. (2016) 38:326–30. 10.1111/pim.12314 26971490

[286] Humen MA De Antoni GL Benyacoub J Costas ME Cardozo MI Kozubsky L. *Lactobacillus johnsonii* La1 antagonizes Giardia intestinalis *in vivo*. Infect Immun. (2005) 73:1265–9. 10.1128/IAI.73.2.1265-1269.2005 15664978 PMC547090

[287] Del Coco VF Sparo MD Sidoti A Santín M Basualdo JA Córdoba MA. Effects of *Enterococcus faecalis* CECT 7121 on *Cryptosporidium parvum* infection in mice. Parasitol Res. (2016) 115:3239–44. 10.1007/s00436-016-5087-1 27193238

[288] Khalifa EA. Probiotics as a promising treatment of experimental cryptosporidiosis in an immuno suppressed mouse model. Int J Curr Microbiol App Sci. (2016) 5:97–106. 10.20546/ijcmas.2016.503.014

[289] Mohammed S Jabur K Aja H. Effect of Yogurt and bifidobacterium on *Cryptosporidium parvum* infection in experiential infected mice. Ibn AL Haitham J Pure Appl Sci. (2017) 24:40–8.

[290] Oliveira BC Widmer G. Probiotic product enhances susceptibility of mice to cryptosporidiosis. Appl Environ Microbiol. (2018) 84:e01408–18. 10.1128/AEM.01408-18 30171003 PMC6193388

[291] Santos JdFM Vasconcelos J Souza JRd Coutinho EdM Montenegro SML Azevedo-Ximenes E. The effect of *Zymomonas mobilis* culture on experimental *Schistosoma mansoni* infection. Rev Soc Bras Med Trop. (2004) 37:502–4. 10.1590/S0037-86822004000600015 15765603

[292] Martínez-Gómez F Santiago-Rosales R Bautista-Garfias CR. Effect of *Lactobacillus casei* Shirota strain intraperitoneal administration in CD1 mice on the establishment of *Trichinella spiralis* adult worms and on IgA anti-*T*. spiralis production. Vet Parasitol. (2009) 162:171–5. 10.1016/j.vetpar.2009.02.010 19269100

[293] Chiodo PG Sparo MD Pezzani BC Minvielle MC Basualdo JA. *In vitro* and *in vivo* effects of *Enterococcus faecalis* CECT7121 on *Toxocara canis*. Memórias Inst Oswaldo Cruz. (2010) 105:615–20. 10.1590/S0074-02762010000500003 20835606

[294] Dea-Ayuela MA Rama-Iñiguez S Bolás-Fernandez F. Enhanced susceptibility to *Trichuris muris* infection of B10Br mice treated with the probiotic *Lactobacillus casei*. Int Immunopharmacol. (2008) 8:28–35. 10.1016/j.intimp.2007.10.003 18068097

[295] Ansari F Pourjafar H Tabrizi A Homayouni A. The effects of probiotics and prebiotics on mental disorders: a review on depression, anxiety, Alzheimer, and autism spectrum disorders. Curr Pharm Biotechnol. (2020) 21:555–65. 10.2174/1389201021666200107113812 31914909

[296] Kong X-J Liu J Li J Kwong K Koh M Sukijthamapan P. Probiotics and oxytocin nasal spray as neuro-social-behavioral interventions for patients with autism spectrum disorders: a pilot randomized controlled trial protocol. Pilot Feasibil Stud. (2020) 6:20. 10.1186/s40814-020-0557-8 32082606 PMC7017510

[297] Wieërs G Belkhir L Enaud R Leclercq S Philippart de Foy J-M Dequenne I. How probiotics affect the microbiota. Front Cell Infect Microbiol. (2020) 9:454. 10.3389/fcimb.2019.00454 32010640 PMC6974441

[298] Liu QF Kim H-M Lim S Chung M-J Lim C-Y Koo B-S. Effect of probiotic administration on gut microbiota and depressive behaviors in mice. DARU J Pharm Sci. (2020) 28:181–9. 10.1007/s40199-020-00329-w 32006344 PMC7214553

[299] Zhu S Jiang Y Xu K Cui M Ye W Zhao G. The progress of gut microbiome research related to brain disorders. J Neuroinflamm. (2020) 17:25. 10.1186/s12974-020-1705-z 31952509 PMC6969442

[300] Bravo JA Forsythe P Chew MV Escaravage E Savignac HM Dinan TG. Ingestion of *Lactobacillus* strain regulates emotional behavior and central GABA receptor expression in a mouse via the vagus nerve. Proc Natl Acad Sci USA. (2011) 108:16050–5. 10.1073/pnas.1102999108 21876150 PMC3179073

[301] Desbonnet L Garrett L Clarke G Bienenstock J Dinan TG. The probiotic Bifidobacteria infantis: an assessment of potential antidepressant properties in the rat. J Psychiatr Res. (2008) 43:164–74. 10.1016/j.jpsychires.2008.03.009 18456279

[302] Desbonnet L Garrett L Clarke G Kiely B Cryan JF Dinan TG. Effects of the probiotic *Bifidobacterium infantis* in the maternal separation model of depression. Neuroscience. (2010) 170:1179–88. 10.1016/j.neuroscience.2010.08.005 20696216

[303] Li N Wang Q Wang Y Sun A Lin Y Jin Y. Oral probiotics ameliorate the behavioral deficits induced by chronic mild stress in mice via the gut microbiota-inflammation axis. Front Behav Neurosci. (2018) 12:266. 10.3389/fnbeh.2018.00266 30459574 PMC6232506

[304] Saliganti V Kapila R Sharma R Kapila S. Feeding probiotic *Lactobacillus rhamnosus* (MTCC 5897) fermented milk to suckling mothers alleviates ovalbumin-induced allergic sensitisation in mice offspring. Br J Nutr. (2015) 114:1168–79. 10.1017/S000711451500286X 26330132

[305] George Kerry R Patra JK Gouda S Park Y Shin HS Das G. Benefaction of probiotics for human health: a review. J Food Drug Anal. (2018) 26:927–39. 10.1016/j.jfda.2018.01.002 29976412 PMC9303019

[306] Liu M-Y Yang Z-Y Dai W-K Huang J-Q Li Y-H Zhang J. Protective effect of *Bifidobacterium infantis* CGMCC313-2 on ovalbumin-induced airway asthma and β-lactoglobulin-induced intestinal food allergy mouse models. World J Gastroenterol. (2017) 23:2149. 10.3748/wjg.v23.i12.2149 28405142 PMC5374126

[307] Zhang B An J Shimada T Liu S Maeyama K. Oral administration of *Enterococcus faecalis* FK-23 suppresses Th17 cell development and attenuates allergic airway responses in mice. Int J Mol Med. (2012) 30:248–54. 10.3892/ijmm.2012.1010 22641478

[308] Kim TS Song J Lim HX Lee A Lee J-H. *Staphylococcus succinus* 14BME20 prevents allergic airway inflammation by induction of regulatory T cells via interleukin-10. Front Immunol. (2019) 10:1269. 10.3389/fimmu.2019.01269 31231389 PMC6559308

[309] Zhang J Su H Li Q Wu H Liu M Huang J. Oral administration of *Clostridium butyricum* CGMCC0313-1 inhibits β-lactoglobulin-induced intestinal anaphylaxis in a mouse model of food allergy. Gut Pathog. (2017) 9:11. 10.1186/s13099-017-0160-6 28250847 PMC5322677

[310] Li A-l Meng X-c Duan C-c Huo G-c Zheng Q-l Li D. Suppressive effects of oral administration of heat-killed *Lactobacillus acidophilus* on Th17 immune responses in a bovine β-lactoglobulin-sensitized mice model. Biol Pharm Bull. (2012) 36:202–7. 10.1248/bpb.b12-00437 23207873

[311] Yadav R Khan SH Mada SB Meena S Kapila R Kapila S. Consumption of probiotic *Lactobacillus fermentum* MTCC: 5898-fermented milk attenuates dyslipidemia, oxidative stress, and inflammation in male rats fed on cholesterol-enriched diet. Probiotics Antimicrob Proteins. (2019) 11:509–18. 10.1007/s12602-018-9429-4 29754388

[312] Yadav R Vij R Kapila S Khan SH Kumar N Meena S. Milk fermented with probiotic strains *Lactobacillus rhamnosus* MTCC: 5957 and *Lactobacillus rhamnosus* MTCC: 5897 ameliorates the diet-induced hypercholesterolemia in rats. Ann Microbiol. (2019) 69:483–94. 10.1007/s13213-018-1433-0

[313] Lewis-Mikhael A-M Davoodvandi A Jafarnejad S. Effect of *Lactobacillus plantarum* containing probiotics on blood pressure: a systematic review and meta-analysis. Pharmacol Res. (2020) 153:104663. 10.1016/j.phrs.2020.104663 31987993

[314] Liu DM Guo J Zeng XA Sun DW Brennan CS Zhou QX. The probiotic role of *Lactobacillus plantarum* in reducing risks associated with cardiovascular disease. Int J Food Sci Technol. (2017) 52:127–36. 10.1111/ijfs.13234

[315] Robles-Vera I Toral M de la Visitación N Sánchez M Gómez-Guzmán M Romero M. Probiotics prevent dysbiosis and the raise in blood pressure in genetic hypertension: role of short-chain fatty acids. Mol Nutr Food Res. (2020) 64:e1900616. 10.1002/mnfr.201900616 31953983

[316] Gan XT Ettinger G Huang CX Burton JP Haist JV Rajapurohitam V. Probiotic administration attenuates myocardial hypertrophy and heart failure after myocardial infarction in the rat. Circulation Heart Failure. (2014) 7:491–9. 10.1161/CIRCHEARTFAILURE.113.000978 24625365

[317] Lam V Su J Koprowski S Hsu A Tweddell JS Rafiee P. Intestinal microbiota determine severity of myocardial infarction in rats. FASEB J. (2012) 26:1727–35. 10.1096/fj.11-197921 22247331 PMC3316900

[318] Yadav R Dey DK Vij R Meena S Kapila R Kapila S. Evaluation of anti-diabetic attributes of *Lactobacillus rhamnosus* MTCC: 5957, *Lactobacillus rhamnosus* MTCC: 5897 and *Lactobacillus fermentum* MTCC: 5898 in streptozotocin induced diabetic rats. Microb Pathog. (2018) 125:454–62. 10.1016/j.micpath.2018.10.015 30316007

[319] Wang Y Dilidaxi D Wu Y Sailike J Sun X Nabi X-h. Composite probiotics alleviate type 2 diabetes by regulating intestinal microbiota and inducing GLP-1 secretion in db/db mice. Biomed Pharmacother. (2020) 125:109914. 10.1016/j.biopha.2020.109914 32035395

[320] Aponte M Murru N Shoukat M. Therapeutic, prophylactic, and functional use of probiotics: a current perspective. Front Microbiol. (2020) 11:2120. 10.3389/fmicb.2020.562048 33042069 PMC7516994

[321] Majewska K Kregielska-Narozna M Jakubowski H Szulińska M Bogdański P. The multispecies probiotic effectively reduces homocysteine concentration in obese women: a randomized double-blind placebo-controlled study. J Clin Med. (2020) 9:998. 10.3390/jcm9040998 32252416 PMC7230928

[322] Sanders ME Guarner F Guerrant R Holt PR Quigley EM Sartor RB. An update on the use and investigation of probiotics in health and disease. Gut. (2013) 62:787–96. 10.1136/gutjnl-2012-302504 23474420 PMC4351195

[323] Pei R Martin DA DiMarco DM Bolling BW. Evidence for the effects of yogurt on gut health and obesity. Crit Rev Food Sci Nutr. (2017) 57:1569–83. 10.1080/10408398.2014.883356 25875150

[324] Gérard P. Gut microbiota and obesity. Cell Mol Life Sci. (2016) 73:147–62. 10.1007/s00018-015-2061-5 26459447 PMC11108539

[325] Serban DE. Gastrointestinal cancers: influence of gut microbiota, probiotics and prebiotics. Cancer Lett. (2014) 345:258–70. 10.1016/j.canlet.2013.08.013 23981580

[326] Kumar M Nagpal R Verma V Kumar A Kaur N Hemalatha R. Probiotic metabolites as epigenetic targets in the prevention of colon cancer. Nutr Rev. (2013) 71:23–34. 10.1111/j.1753-4887.2012.00542.x 23282249

[327] Vipperla K O'Keefe SJ. The microbiota and its metabolites in colonic mucosal health and cancer risk. Nutr Clin Prac. (2012) 27:624–35. 10.1177/0884533612452012 22868282

[328] Tedelind S Westberg F Kjerrulf M Vidal A. Anti-inflammatory properties of the short-chain fatty acids acetate and propionate: a study with relevance to inflammatory bowel disease. World J Gastroenterol. (2007) 13:2826. 10.3748/wjg.v13.i20.2826 17569118 PMC4395634

[329] Urbanska AM Paul A Bhahena J Prakash S. Suppression of tumorigenesis: modulation of inflammatory cytokines by oral administration of microencapsulated probiotic yogurt formulation. Int J Inflam. (2010) 2011:891015. 10.4061/2010/894972 21188222 PMC3004002

[330] Zhu Q Gao R Wu W Qin H. The role of gut microbiota in the pathogenesis of colorectal cancer. Tumor Biol. (2013) 34:1285–300. 10.1007/s13277-013-0684-4 23397545

[331] dos Reis SA da Conceição LL Siqueira NP Rosa DD da Silva LL Maria do Carmo GP. Review of the mechanisms of probiotic actions in the prevention of colorectal cancer. Nutr Res. (2017) 37:1–19. 10.1016/j.nutres.2016.11.009 28215310

[332] Pala V Sieri S Berrino F Vineis P Sacerdote C Palli D. Yogurt consumption and risk of colorectal cancer in the Italian European prospective investigation into cancer and nutrition cohort. Int J Cancer. (2011) 129:2712–9. 10.1002/ijc.26193 21607947

[333] Saliganti V Kapila R Kapila S. Consumption of probiotic *Lactobacillus rhamnosus* (MTCC: 5897) containing fermented milk plays a key role in development of the immune system in newborn mice during the suckling–weaning transition. Microbiol Immunol. (2016) 60:261–7. 10.1111/1348-0421.12342 26589556

[334] Nomura A Matsubara A Goto S Takahata J Sawada K Ihara K. Relationship between gut microbiota composition and sensitization to inhaled allergens. Allergol Int. (2020) 69:437–42. 10.1016/j.alit.2019.12.010 31980373

[335] Yan F Li N Yue Y Wang C Zhao L Evivie SE. Screening for potential novel probiotics with dipeptidyl peptidase IV-inhibiting activity for type 2 diabetes attenuation *in vitro* and *in vivo*. Front Microbiol. (2020) 10:2855. 10.3389/fmicb.2019.02855 31998245 PMC6965065

[336] Zhang Z Liang X Lv Y Yi H Chen Y Bai L. Evaluation of probiotics for improving and regulation metabolism relevant to type 2 diabetes *in vitro*. J Funct Foods. (2020) 64:103664. 10.1016/j.jff.2019.103664

[337] Lee Y-S Jun H-S. Anti-diabetic actions of glucagon-like peptide-1 on pancreatic beta-cells. Metab Clin Exp. (2014) 63:9–19. 10.1016/j.metabol.2013.09.010 24140094

[338] Timper K Dalmas E Dror E Rütti S Thienel C Sauter NS. Glucose-dependent insulinotropic peptide stimulates glucagon-like peptide 1 production by pancreatic islets via interleukin 6, produced by α cells. Gastroenterology. (2016) 151:165–79. 10.1053/j.gastro.2016.03.003 26971825

[339] Wang L Li P Tang Z Yan X Feng B. Structural modulation of the gut microbiota and the relationship with body weight: compared evaluation of liraglutide and saxagliptin treatment. Sci Rep. (2016) 6:33251. 10.1038/srep33251 27633081 PMC5025740

[340] Tiderencel KA Hutcheon DA Ziegler J. Probiotics for the treatment of type 2 diabetes: a review of randomized controlled trials. Diabetes Metab Res Rev. (2020) 36:e3213. 10.1002/dmrr.3213 31465625

[341] Abasi S Keshtmand Z. The effect of probiotic bifidobacterium lactis and *Lactobacillus casei* on sperm maturation in streptozotocin-diabetic rats. ISMJ. (2020) 22:392–401. 10.29252/ismj.22.6.392

[342] Dardmeh F Alipour H Gazerani P van der Horst G Brandsborg E Nielsen HI. *Lactobacillus rhamnosus* PB01 (DSM 14870) supplementation affects markers of sperm kinematic parameters in a diet-induced obesity mice model. PLoS ONE. (2017) 12:e185964. 10.1371/journal.pone.0185964 29016685 PMC5634625

[343] Vílchez MC Santangeli S Maradonna F Gioacchini G Verdenelli C Gallego V. Effect of the probiotic *Lactobacillus rhamnosus* on the expression of genes involved in European eel spermatogenesis. Theriogenology. (2015) 84:1321–31. 10.1016/j.theriogenology.2015.07.011 26271165

[344] Inatomi T Otomaru K. Effect of dietary probiotics on the semen traits and antioxidative activity of male broiler breeders. Sci Rep. (2018) 8:1–6. 10.1038/s41598-018-24345-8 29651158 PMC5897344

[345] Jennifer B. *Probiotics and cardiovascular disease* (dissertation/master's thesis), Nevada, ND: University of North Dakota (2017).

[346] Thushara RM Gangadaran S Solati Z Moghadasian MH. Cardiovascular benefits of probiotics: a review of experimental and clinical studies. Food Funct. (2016) 7:632–42. 10.1039/C5FO01190F 26786971

[347] Tomaro-Duchesneau C Jones ML Shah D Jain P Saha S Prakash S. Cholesterol assimilation by Lactobacillus probiotic bacteria: an *in vitro* investigation. Biomed Res Int. (2014) 2014:380316. 10.1155/2014/380316 25295259 PMC4176637

[348] Vasquez EC Pereira T Peotta VA Baldo MP Campos-Toimil M. Probiotics as beneficial dietary supplements to prevent and treat cardiovascular diseases: uncovering their impact on oxidative stress. Oxid Med Cell Longev. (2019) 2019:3086270. 10.1155/2019/3086270 31205584 PMC6530239

[349] March DS Jones AW Bishop NC Burton JO. The efficacy of prebiotic, probiotic, and synbiotic supplementation in modulating gut-derived circulatory particles associated with cardiovascular disease in individuals receiving dialysis: a systematic review and meta-analysis of randomized controlled trials. J Renal Nutr. (2019) 30:347–59. 10.1053/j.jrn.2019.07.006 31607550

